# Supplementary material for: The first quantitative assessment of radiocarbon chronologies for initial pottery in Island Southeast Asia supports multi-directional Neolithic dispersal
Source: PLoS One. 2021 Jun 2;16(6):e0251407. doi: 10.1371/journal.pone.0251407 (PMC8171956; doi:10.1371/journal.pone.0251407)
Supplement: S1 File — (DOCX) [file pone.0251407.s001.docx]

The first quantitative assessment of radiocarbon chronologies for initial pottery in Island Southeast Asia supports multi-directional Neolithic dispersal

Ethan E. Cochrane^1¶^*, Timothy M. Rieth^2¶^, Darby Filimoehala^2^

^1^Anthropology, The University of Auckland, Auckland, New Zealand

^2^International Archaeological Research Institute Inc., Honolulu, USA

* Corresponding author

E-mail: e.cochrane@auckland.ac.nz (EC)

^¶^These authors contributed equally to the work.

**Descriptions and Oxcal codes for models included in the analysis**

The model descriptions, results, and code are presented in the same order as the results discussed in the main text. For many site deposits two or more models were created and the codes for these are presented below. It is important to note that most of the models are hindered by one or more issues: too few radiocarbon determinations, unclear stratigraphic information presented in publications, uncertain localized correction values, and the possibility of inbuilt age. Within the constraints of these limitations, we justify our preferred models with the caveat that additional dates and other information (e.g., correction values) should improve the chronological calculations. The results of our non-preferred models do not significantly change our conclusions.

**Batanes Islands**

Two single-phase models were created for site deposits on Batan [1-5] and Itbayat [6] Islands (S1 Fig). Determinations from these site deposits and three other site deposits or excavation areas (S3 Table) that produced individual radiocarbon ages (Mahatao Town Septic Tank [3, 4] and Tayid [2]) were included in single-phase models for each respective island.

Two island-scale models were created for Batan Island: one model includes determinations from unidentified charcoal and residue on sherds, while another model excludes the determinations from the sherd residue. The second model evaluates the possibility that sherd residue determinations are older than stratigraphically associated charcoal ages and are therefore potentially problematic [7]. For those sites that produced radiocarbon determinations from stratified deposits, multi-phase models were created.

Dating samples were unidentified wood charcoal, residue on sherds, and one sample of “resin” coating a sherd. Three Batanes radiocarbon determinations were excluded from the models: ANU-11709 (unidentified charcoal; 200±360 BP) is anomalously young and must be an intrusive specimen [2: 71, Table 5.1]; OZH-776 (resin; 5790±150 BP) was identified as being anomalously old by Bellwood and Dizon [2:70] and there is a question about what comprises the resin and how suitable it is for dating of the associated ceramic; and Gif-6575 (unidentified charcoal; 2310±80 BP) from the Basco site on Batan Island [8:13] was excluded because it cannot be clearly associated with a ceramic deposit.

**Sunget Site, Batan Island**

A single-phase model including determinations from the main and top terrace excavations results in a date for the appearance of ceramics at Sunget sometime between *4330-2890 cal BP (95.4%) or 3490-3000 cal BP (68.2%)*. The model contains five radiocarbon determinations, three of which were obtained from unidentified charcoal (ANU-11693, Wk-15649, and ANU-11707) with the other two from charred residue on ceramic sherds (Wk-14640 and ANU-11817).

Plot()

{

Outlier_Model("General",T(5),U(0,2.3),"t");

Outlier_Model("Charcoal",Exp(1,-10,0),U(0,2.3),"t");

Sequence("Batan Sunget")

{

Boundary("start");

Phase("Sunget")

{

R_Date("Wk-14640",2915,49)

{

Outlier("General",0.05);

};

R_Date("ANU-11817",2910,190)

{

Outlier("General",0.05);

};

R_Date("ANU-11693",2630,30)

{

Outlier("Charcoal",1);

};

R_Date("Wk-15649",2383,35)

{

Outlier("Charcoal",1);

};

R_Date("ANU-11707",2000,140)

{

Outlier("Charcoal",1);

};

};

Boundary("end");

};

};

**Batan Island**

The five determinations from the Sunget models were combined with seven determinations from the Naidi (unidentified charcoal), Payaman (unidentified charcoal), Mahatao (unidentified charcoal), and Tayid (residue on potsherd) sites in a single-phase, island-scale model for Batan. At this geographic scale, the appearance of pottery is calculated to have occurred sometime between *3640-2890 cal BP (95.4%) or 3310-2990 cal BP (68.2%)*. This date is significantly younger and more precise than the site-scale date due to the increased number of determinations included in the model. The dated Batan Island pottery assemblages include red-slip and circle-stamp surface treatments [9: Figure 6.13].

Plot()

{

Outlier_Model("General",T(5),U(0,2.3),"t");

Outlier_Model("Charcoal", Exp(1,-10,0), U(0,2.3), "t");

Sequence("Batan Island")

{

Boundary("start");

Phase("Batan")

{

R_Date("Wk-14640",2915,49)

{

Outlier("General",0.05);

};

R_Date("ANU-11817",2910,190)

{

Outlier("General",0.05);

};

R_Date("ANU-11693",2630,30)

{

Outlier("Charcoal",1);

};

R_Date("ANU-11695

",2620,30)

{

Outlier("Charcoal",1);

};

R_Date("Wk-15649",2383,35)

{

Outlier("Charcoal",1);

};

R_Date("ANU-11708",2240,140)

{

Outlier("Charcoal",1);

};

R_Date("ANU-11710",2090,60)

{

Outlier("Charcoal",1);

};

R_Date("ANU-11707",2000,140)

{

Outlier("Charcoal",1);

};

R_Date("Wk-13092",1988,47)

{

Outlier("Charcoal",1);

};

R_Date("ANU-12069",1842,215)

{

Outlier("General",0.05);

};

R_Date("ANU-11694",1590,210)

{

Outlier("Charcoal",1);

};

R_Date("ANU-12068",1486,185)

{

Outlier("Charcoal",1);

};

};

Boundary("end");

};

};

**Batan Island without residue determinations**

The three determinations from unidentified charcoal from the Sunget model were combined with six determinations from the Naidi, Payaman, and Mahatao sites in a single-phase, island-scale model for Batan; this model excludes three determinations obtained from residue on sherds from Sunget (Wk-14640 and ANU-11817) and Tayid (ANU-12069). The appearance of pottery is calculated to have occurred sometime between *3380-2590 cal BP (95.4%) or 2970-2730 cal BP (68.2%)*. This date is younger than the island-scale model that includes the sherd residue determinations (*3640-2890 cal BP [95.4%]*). It is apparent that the determinations from sherd residue return older calibrated dates, which affect the modeled results.

Plot()

{

Outlier_Model("Charcoal", Exp(1,-10,0), U(0,2.3), "t");

Sequence("Batan Island")

{

Boundary("start");

Phase("Batan")

{

R_Date("ANU-11693",2630,30)

{

Outlier(1);

};

R_Date("ANU-11695",2620,30)

{

Outlier(1);

};

R_Date("Wk-15649",2383,35)

{

Outlier(1);

};

R_Date("ANU-11708",2240,140)

{

Outlier(1);

};

R_Date("ANU-11710",2090,60)

{

Outlier(1);

};

R_Date("ANU-11707",2000,140)

{

Outlier(1);

};

R_Date("Wk-13092",1988,47)

{

Outlier(1);

};

R_Date("ANU-11694",1590,210)

{

Outlier(1);

};

R_Date("ANU-12068",1486,185)

{

Outlier(1);

};

};

Boundary("end");

};

};

**Anaro Hilltop, Itbayat Island**

A single-phase model for the Anaro Hilltop site consists of determinations from Area 2A, Area 3, Area 3A, and Area 3B (nine determinations, all obtained from charred residue on ceramic sherds); determinations from the different excavation areas were aggregated to increase the model’s sample size, which should positively affect accuracy and precision. The site-scale model dates the onset of ceramics sometime between *3470-2760 cal BP (95.4%) or 3070-2790 cal BP (68.2%)*. As noted above for Batan Island, dating charred residue may require a correction value or otherwise produce inaccurate dates, so these results should be evaluated against future determinations derived from other sample materials. The pottery associated with the Anaro Hilltop date includes circle-stamp surface treatment [9].

Plot()

{

Outlier_Model("General",T(5),U(0,2.3),"t");

Sequence("Itbayat_Anaro_Hilltop")

{

Boundary("start");

Phase("Anaro_Hilltop")

{

R_Date("OZH-774",2770,50)

{

Outlier("General",0.05);

};

R_Date("OZJ-693",2475,45)

{

Outlier("General",0.05);

};

R_Date("OZJ-692",2095,45)

{

Outlier("General",0.05);

};

R_Date("OZJ-695",2080,45)

{

Outlier("General",0.05);

};

R_Date("Wk-14643",1876,41)

{

Outlier("General",0.05);

};

R_Date("OZJ-697",1510,60)

{

Outlier("General",0.05);

};

R_Date("OZJ-696",1375,35)

{

Outlier("General",0.05);

};

R_Date("Wk-14645",1360,39)

{

Outlier("General",0.05);

};

R_Date("OZJ-694",1280,45)

{

Outlier("General",0.05);

};

};

Boundary("end");

};

};

**Itbayat Island**

The nine determinations from the Anaro Hilltop model were combined with two determinations (residue on ceramic sherds) from Torongan Cave in a single-phase, island-scale model for Itbayat Island. At this scale, the appearance of pottery is calculated to have occurred sometime between *5200-3990 cal BP (95.4%) or 4620-4150 cal BP (68.2%)*. The two Torongan Cave determinations seem to be driving the date range and this model is potentially problematic for the same reason described for Anaro Hilltop (i.e., the unknown effects of “charred residue” determinations).

Plot()

{

Outlier_Model("General",T(5),U(0,2.3),"t");

Sequence("Itbayat_Island")

{

Boundary("start");

Phase("Itbayat")

{

R_Date("OZH-771",3860,70)

{

Outlier("General",0.05);

};

R_Date("Wk-14642",3320,40)

{

Outlier("General",0.05);

};

R_Date("OZH-774",2770,50)

{

Outlier("General",0.05);

};

R_Date("OZJ-693",2475,45)

{

Outlier("General",0.05);

};

R_Date("OZJ-692",2095,45)

{

Outlier("General",0.05);

};

R_Date("OZJ-695",2080,45)

{

Outlier("General",0.05);

};

R_Date("Wk-14643",1876,41)

{

Outlier("General",0.05);

};

R_Date("OZJ-697",1510,60)

{

Outlier("General",0.05);

};

R_Date("OZJ-696",1375,35)

{

Outlier("General",0.05);

};

R_Date("Wk-14645",1360,39)

{

Outlier("General",0.05);

};

R_Date("OZJ-694",1280,45)

{

Outlier("General",0.05);

};

};

Boundary("end");

};

};

**Philippines**

Ceramic-bearing archaeological deposits on the Philippine islands of Luzon, Masbate, and Negros have reported radiocarbon determinations, though the majority of this corpus is from excavations on Luzon. Eight single- or multi-phase models were created for site deposits from Nagsabaran [5, 10-12] on Luzon Island (S2 Fig). Determinations from this site, Andarayan [13], Irigayen [14], Callao Cave [15], and Dalan Serkot [15] were included in a single-phase model for the island.

Philippine dating samples are unidentified wood charcoal, unidentified animal bone, unidentified shell, freshwater shell (*Batissa childreni*), pig (*Sus scrofa/verrucosus*), rice husk and stem fragments, and unreported material [10, 11, 13:5, 14:30, 15-18, 19:691, 20:78, 21]. Isotopic and quality control data were obtained from the Waikato Radiocarbon Dating Laboratory for an unidentified animal bone (Wk-19712, 2504±35) and a pig tooth (Wk-23997 [mistakenly reported as Wk-23397, F. Petchey pers. comm.], 3940±40 BP) [19:691, 20:78]. Though data for each sample raises possible concerns (see S3 Table), the isotopic data indicate calibration using the atmospheric curve so these determinations were included in our analysis. Isotopic and quality control data are not available for other determinations obtained from unidentified animal bone (NUTA2-7938, 3810±30 BP; NUTA2-7939, 3485±30 BP; NUTA2-7940, 3665±35 BP) [5:159, Table 7.1, 14:30] and these determinations were excluded since it is unknown whether a mixed atmospheric and marine calibration is warranted. An unidentified shell (Gak-7048, 3680±110 BP) [22:80] is also excluded as it is unreported whether the sample is from a terrestrial, freshwater, or marine invertebrate. In addition, two determinations obtained from contexts where the associations with ceramics are ambiguous (Gak-17967, 3390±100 BP; Gak-17968, 3810±200 BP) [23:132, 24:67] were excluded.

The Nagsabaran site-scale and Luzon Island models were run with freshwater shell-derived determinations using IntCal20. Hung et al. [11] evaluated the possibility of a freshwater reservoir effect by dating multiple modern specimens, none of which exhibit significant inputs of older carbon from limestone substrate or the marine reservoir. The modeling results for Nagsabaran, however, raise the possibility that this may not be the case for the millennia-old archaeological shell specimens as these may been subjected to different environmental carbon inputs compared to modern specimens (see [25] for an example from Micronesia). We discuss the modelling results in light of site formation processes in the next section.

Individual determinations on unidentified charcoal from the Edjeck Site, Negros Island [18] and Cave 2 on Masbate Island [21] were not modeled since they represent the sole dates for each island. The two determinations from the Bagumbayan Site, Masbate Island [16] are on unreported material and are excluded.

**Nagsabaran Site, Luzon Island**

Nagsabaran is an important site for research on the Neolithic in ISEA as multiple projects have been undertaken at the site, several publications produced, and a large number of dates presented. Our chronological modeling of Nagsabaran site deposits required a more involved procedure than for other sites. A corpus of 33 radiocarbon determinations for archaeological contexts has been published with provenience information of varying quality. Broadly, the determinations are associated with the lower silt layers (n = 17) and onset of ceramic deposition or the stratigraphically superior shell midden (n = 16). Hung et al. [11] identify 11 determinations as too old (silt: GX-26705, 6610±290; Wk-19713, 4450±39; shell midden: ANU-13018, 7380±40; ANU-13017, 3420±30) or too young (silt: NTU-3798, 2670±40; ANU-13014, 2660±30; GX-26704-AMS, 2620±40; ANU-13013, 2540±30; Wk-17756, 2528±31; GX-26711-AMS, 2520±50; Wk-18059, 1946±30) for respective contexts. The range of CRAs for the silt layers is 6610±290 (GX-26705) to 1946±30 (Wk-18059) and for the shell midden is 7380±40 (ANU-13018) to 1470±50 (GX-26797). Considering published sources, there are several reasons to think that both redeposition of charcoal and vertical movement of dating samples may account for both the wide range of CRAs, and radiocarbon determinations inverted relative to depth and stratigraphy: the site is less than 500 m from the broad Cagayan river and immediately adjacent to a creek; the silt layers are alluvium and the upper deposits are a coarse shell midden with multiple pit features, making secondary alluvial deposition and vertical movement, respectively, more likely [as noted by 11]; none of the charcoal samples from the lower depths of the silt layers are from archaeological features; and there are “a small number of identifiable tree root holes, these also being a major source of disturbance” (but the extent of tree root disturbance across the site is unclear from Hung et al. [11:3]).

According to Hung et al. [11] the silt layers have large post-holes immediately below the shell midden layer that penetrate deep into the silt. These post-holes represent “a settlement of houses raised on stilts that was in existence when the shell midden began” [11:2] (unfortunately, no such post-holes are visible in the four published stratigraphic sections we have found [5, 26]). For the silt layers themselves, there are “no signs [i.e., archaeological features] of actual human occupation”, except for the red-slipped pottery, “with charcoal only surviving where protected beneath sherds or postholes” [11:2] (although it appears that a post-hole or some feature is associated with earlier silt layer deposition in Pits 11 and 12 as depicted in [26:Figure 1.5]). Given that Hung et al. [11] note that post holes are associated with the start, or just prior to the start, of the shell midden, charcoal from these post-holes in the silt layers should not be associated with the onset of ceramic deposition in the lower depths of the silt layers. Unfortunately, no publications include contextual association of dating samples indicating whether they derive from post-holes or under sherds or are associated with root activity [cf. 12: Table 1]. Only depths are presented.

Given these issues and the lack of clear contextual information for dating samples, the deposition or movement of these samples could be, and in some instances clearly is, independent of the pottery and material culture recovered from the same contexts. We created multiple chronological models to explore these issues in an objective manner.

To evaluate the two major depositional groups—silts and stratigraphically superior shell midden—single phase models were created for each. Though the upper shell midden does not relate to the initial appearance of pottery at the site, its potential inclusion in a stratigraphically structured two-phase model warranted this initial evaluation. Initial iterations included all determinations; none of the clearly anomalous determinations were excluded. A second iteration of each model excludes the oldest determination from each phase (silt: GX-26705, 6610±290; shell midden: ANU-13018, 7380±40); exclusion of these determinations is defensible based on regional archaeological data and consideration of the suite of determinations for Nagsabaran.

Each of the models was resolved with acceptable convergence values (that is, the model structure and data allow for the resolution of representative results within the range of possible solutions), though the results vary significantly. The start date for lower silt model inclusive of all determinations is *8630-6420 cal BP (95.4%)* or *7860-6890 cal BP (68.2%)*, while the start date for the model that excludes the oldest determination is *5700-4550 cal BP (95.4%)* or *5350-4910 cal BP (68.2%)*. The start date for the upper shell midden, which based on the law of superposition must post-date the lower silts, is *9380-8030 cal BP (95.4%)* or *8590-8070 cal BP (68.2%)* for the all-inclusive model and *4200-3570 cal BP (95.4%)* or *3850-3600 cal BP (68.2%)* for the model excluding the oldest determination.

Based on these results, two-phase models were constructed with a lower silt phase and a shell midden phase in sequential order. One model includes all of the determinations with a second model excluding the oldest determination from each phase. The all-inclusive model is largely for heuristic purposes, since it is apparent that the oldest determination from each phase is anomalous based on the foregoing reasons and the single-phase model results. A third model iteration excludes the oldest determination from the silt layers and the five oldest determinations from the shell midden. The latter are derived from *B. childreni* freshwater shells and were flagged as outliers in the previous model iterations (O: 100/5). That the shell dates are the five oldest from the shell midden layer, and all flagged as outliers, calls into question Hung et al.’s [11] analysis of the modern *B. childreni* dating data and their suggestion that there is no need to apply a correction value to archaeological shell specimens that are potentially millennia older. A fourth model is based on Hung et al.’s [14] subjective, we would argue, determination of outliers, including only those determinations they accept.

The all-inclusive two-phase model provides a start date of *8570-5870 cal BP (95.4%)* or *7820-6730 cal BP (68.2%)*. The start date for the model excluding the oldest determination from each phase is *5620-4290 cal BP (95.4%)* or *5360-4410 (68.2%)*. The start date for the model excluding the oldest determination from the silt layer phase and the five oldest determinations from the shell midden is *5440-4270 cal BP (95.4%)* or *5100-4390 cal BP (68.2%)*. The start date based on Hung et al.’s preferred suite of determinations is *4980-4160 cal BP (95.4%)* or *4590-4290 cal BP (68.2%)*.

Our model iterations reveal the protean nature of the Nagsabaran suite of determinations: different sampling of the corpus provides unique results. The underlying issue is a lack of primary association between some of the dating samples and material culture, which is exacerbated by an inability to objectively determine which radiocarbon ages are problematic, in most cases (the oldest *B. childreni* determinations from the shell midden are an exception). In consideration of this challenge, we prefer the model that excludes the oldest silt layer determination and the five oldest shell midden determinations.

Early Nagsabaran ceramics comprise red-slip, impressed, circle-stamp, and dentate surface treatments [17, 27]

Nagsabaran, Single-Phase, Lower Silt All-inclusive

Options()

{

kIterations=3000;

};

Plot()

{

Outlier_Model("General",T(5),U(0,2.3),"t");

Outlier_Model("Charcoal", Exp(1,-10,0), U(0,2.3), "t");

Sequence("Luzon_Nagsabaran")

{

Boundary("start");

Phase("Lower silts")

{

R_Date("GX-26705",6610,290)

{

Outlier("Charcoal",1);

};

R_Date("Wk-19713",4450,39)

{

Outlier("Charcoal",1);

};

R_Date("Wk-23997",3940,40)

{

Outlier("General",0.05);

};

R_Date("Beta-436818",3760,30)

{

Outlier("Charcoal",1);

};

R_Date("ANU-13016",3510,30)

{

Outlier("Charcoal",1);

};

R_Date("NTU-3799",3450,40)

{

Outlier("General",0.05);

};

R_Date("GX-28381",3390,130)

{

Outlier("Charcoal",1);

};

R_Date("GX-28379",3050,70)

{

Outlier("Charcoal",1);

};

R_Date("NTU-3798",2670,40)

{

Outlier("Charcoal",1);

};

R_Date("ANU-13014",2660,30)

{

Outlier("Charcoal",1);

};

R_Date("GX-26704-AMS",2620,40)

{

Outlier("Charcoal",1);

};

R_Date("Beta-437271",2550,30)

{

Outlier("General",0.05);

};

R_Date("ANU-13013",2540,30)

{

Outlier("Charcoal",1);

};

R_Date("Wk-17756",2528,31)

{

Outlier("Charcoal",1);

};

R_Date("GX-26711-AMS",2520,50)

{

Outlier("Charcoal",1);

};

R_Date("Wk-19712",2504,35)

{

Outlier("General",0.05);

};

R_Date("Wk-18059",1946,30)

{

Outlier("Charcoal",1);

};

};

Boundary("end lower silt");

};

};

Nagsabaran, Single-Phase, Lower Silt Excluding Oldest Determination

Plot()

{

Outlier_Model("General",T(5),U(0,2.3),"t");

Outlier_Model("Charcoal", Exp(1,-10,0), U(0,2.3), "t");

Sequence("Luzon_Nagsabaran")

{

Boundary("start");

Phase("Lower silts")

{

R_Date("Wk-19713",4450,39)

{

Outlier("Charcoal",1);

};

R_Date("Wk-23997",3940,40)

{

Outlier("General",0.05);

};

R_Date("Beta-436818",3760,30)

{

Outlier("Charcoal",1);

};

R_Date("ANU-13016",3510,30)

{

Outlier("Charcoal",1);

};

R_Date("NTU-3799",3450,40)

{

Outlier("General",0.05);

};

R_Date("GX-28381",3390,130)

{

Outlier("Charcoal",1);

};

R_Date("GX-28379",3050,70)

{

Outlier("Charcoal",1);

};

R_Date("NTU-3798",2670,40)

{

Outlier("Charcoal",1);

};

R_Date("ANU-13014",2660,30)

{

Outlier("Charcoal",1);

};

R_Date("GX-26704-AMS",2620,40)

{

Outlier("Charcoal",1);

};

R_Date("Beta-437271",2550,30)

{

Outlier("General",0.05);

};

R_Date("ANU-13013",2540,30)

{

Outlier("Charcoal",1);

};

R_Date("Wk-17756",2528,31)

{

Outlier("Charcoal",1);

};

R_Date("GX-26711-AMS",2520,50)

{

Outlier("Charcoal",1);

};

R_Date("Wk-19712",2504,35)

{

Outlier("General",0.05);

};

R_Date("Wk-18059",1946,30)

{

Outlier("Charcoal",1);

};

};

Boundary("end lower silt");

};

};

Nagsabaran, Single-Phase, Shell Midden All-inclusive

Plot()

{

Outlier_Model("General",T(5),U(0,2.3),"t");

Outlier_Model("Charcoal", Exp(1,-10,0), U(0,2.3), "t");

Sequence("Luzon_Nagsabaran")

{

Boundary("start shell midden");

Phase("shell midden")

{

R_Date("ANU-13018",7380,40)

{

Outlier("General",0.05);

};

R_Date("ANU-13017",3420,30)

{

Outlier("General",0.05);

};

R_Date("ANU-13024",2680,30)

{

Outlier("General",0.05);

};

R_Date("ANU-13020",2620,30)

{

Outlier("General",0.05);

};

R_Date("ANU-13019",2560,30)

{

Outlier("General",0.05);

};

R_Date("GX-26801",2260,270)

{

Outlier("Charcoal",1);

};

R_Date("GX-26802",2240,270)

{

Outlier("Charcoal",1);

};

R_Date("GX-26806",2150,150)

{

Outlier("Charcoal",1);

};

R_Date("GX-26705",2120,220)

{

Outlier("Charcoal",1);

};

R_Date("GX-26799",1960,90)

{

Outlier("Charcoal",1);

};

R_Date("GX-26699",1920,80)

{

Outlier("Charcoal",1);

};

R_Date("GX-26698",1830,70)

{

Outlier("Charcoal",1);

};

R_Date("GX-26702-AMS",1820,40)

{

Outlier("Charcoal",1);

};

R_Date("GX-26800",1760,110)

{

Outlier("Charcoal",1);

};

R_Date("GX-26798",1670,60)

{

Outlier("Charcoal",1);

};

R_Date("GX-26797",1470,50)

{

Outlier("Charcoal",1);

};

};

Boundary("end");

};

};

Nagsabaran, Single-Phase, Shell Midden Excluding Oldest Determination

Options()

{

kiterations=100;

};

Plot()

{

Outlier_Model("General",T(5),U(0,2.3),"t");

Outlier_Model("Charcoal", Exp(1,-10,0), U(0,2.3), "t");

Sequence("Luzon_Nagsabaran")

{

Boundary("start shell midden");

Phase("shell midden")

{

R_Date("ANU-13017",3420,30)

{

Outlier("General",0.05);

};

R_Date("ANU-13024",2680,30)

{

Outlier("General",0.05);

};

R_Date("ANU-13020",2620,30)

{

Outlier("General",0.05);

};

R_Date("ANU-13019",2560,30)

{

Outlier("General",0.05);

};

R_Date("GX-26801",2260,270)

{

Outlier("Charcoal",1);

};

R_Date("GX-26802",2240,270)

{

Outlier("Charcoal",1);

};

R_Date("GX-26806",2150,150)

{

Outlier("Charcoal",1);

};

R_Date("GX-26705",2120,220)

{

Outlier("Charcoal",1);

};

R_Date("GX-26799",1960,90)

{

Outlier("Charcoal",1);

};

R_Date("GX-26699",1920,80)

{

Outlier("Charcoal",1);

};

R_Date("GX-26698",1830,70)

{

Outlier("Charcoal",1);

};

R_Date("GX-26702-AMS",1820,40)

{

Outlier("Charcoal",1);

};

R_Date("GX-26800",1760,110)

{

Outlier("Charcoal",1);

};

R_Date("GX-26798",1670,60)

{

Outlier("Charcoal",1);

};

R_Date("GX-26797",1470,50)

{

Outlier("Charcoal",1);

};

};

Boundary("end");

};

};

Nagsabaran, Two-Phase, All-inclusive

Options()

{

kIterations=300;

};

Plot()

{

Outlier_Model("General",T(5),U(0,2.3),"t");

Outlier_Model("Charcoal", Exp(1,-10,0), U(0,2.3), "t");

Sequence("Luzon_Nagsabaran")

{

Boundary("start");

Phase("Lower silts")

{

R_Date("GX-26705",6610,290)

{

Outlier("Charcoal",1);

};

R_Date("Wk-19713",4450,39)

{

Outlier("Charcoal",1);

};

R_Date("Wk-23997",3940,40)

{

Outlier("General",0.05);

};

R_Date("Beta-436818",3760,30)

{

Outlier("Charcoal",1);

};

R_Date("ANU-13016",3510,30)

{

Outlier("Charcoal",1);

};

R_Date("NTU-3799",3450,40)

{

Outlier("General",0.05);

};

R_Date("GX-28381",3390,130)

{

Outlier("Charcoal",1);

};

R_Date("GX-28379",3050,70)

{

Outlier("Charcoal",1);

};

R_Date("NTU-3798",2670,40)

{

Outlier("Charcoal",1);

};

R_Date("ANU-13014",2660,30)

{

Outlier("Charcoal",1);

};

R_Date("GX-26704-AMS",2620,40)

{

Outlier("Charcoal",1);

};

R_Date("Beta-437271",2550,30)

{

Outlier("General",0.05);

};

R_Date("ANU-13013",2540,30)

{

Outlier("Charcoal",1);

};

R_Date("Wk-17756",2528,31)

{

Outlier("Charcoal",1);

};

R_Date("GX-26711-AMS",2520,50)

{

Outlier("Charcoal",1);

};

R_Date("Wk-19712",2504,35)

{

Outlier("General",0.05);

};

R_Date("Wk-18059",1946,30)

{

Outlier("Charcoal",1);

};

};

Boundary("end lower silts");

Boundary("start shell midden");

Phase("shell midden")

{

R_Date("ANU-13018",7380,40)

{

Outlier("General",0.05);

};

R_Date("ANU-13017",3420,30)

{

Outlier("General",0.05);

};

R_Date("ANU-13024",2680,30)

{

Outlier("General",0.05);

};

R_Date("ANU-13020",2620,30)

{

Outlier("General",0.05);

};

R_Date("ANU-13019",2560,30)

{

Outlier("General",0.05);

};

R_Date("GX-26801",2260,270)

{

Outlier("Charcoal",1);

};

R_Date("GX-26802",2240,270)

{

Outlier("Charcoal",1);

};

R_Date("GX-26806",2150,150)

{

Outlier("Charcoal",1);

};

R_Date("GX-26705",2120,220)

{

Outlier("Charcoal",1);

};

R_Date("GX-26799",1960,90)

{

Outlier("Charcoal",1);

};

R_Date("GX-26699",1920,80)

{

Outlier("Charcoal",1);

};

R_Date("GX-26698",1830,70)

{

Outlier("Charcoal",1);

};

R_Date("GX-26702-AMS",1820,40)

{

Outlier("Charcoal",1);

};

R_Date("GX-26800",1760,110)

{

Outlier("Charcoal",1);

};

R_Date("GX-26798",1670,60)

{

Outlier("Charcoal",1);

};

R_Date("GX-26797",1470,50)

{

Outlier("Charcoal",1);

};

};

Boundary("end");

};

};

Nagsabaran, Two-Phase, Two Oldest Determinations Excluded

Options()

{

kIterations=300;

};

Plot()

{

Outlier_Model("General",T(5),U(0,2.3),"t");

Outlier_Model("Charcoal", Exp(1,-10,0), U(0,2.3), "t");

Sequence("Luzon_Nagsabaran")

{

Boundary("start");

Phase("Lower silt")

{

R_Date("Wk-19713",4450,39)

{

Outlier("Charcoal",1);

};

R_Date("Wk-23997",3940,40)

{

Outlier("General",0.05);

};

R_Date("Beta-436818",3760,30)

{

Outlier("Charcoal",1);

};

R_Date("ANU-13016",3510,30)

{

Outlier("Charcoal",1);

};

R_Date("NTU-3799",3450,40)

{

Outlier("General",0.05);

};

R_Date("GX-28381",3390,130)

{

Outlier("Charcoal",1);

};

R_Date("GX-28379",3050,70)

{

Outlier("Charcoal",1);

};

R_Date("NTU-3798",2670,40)

{

Outlier("Charcoal",1);

};

R_Date("ANU-13014",2660,30)

{

Outlier("Charcoal",1);

};

R_Date("GX-26704-AMS",2620,40)

{

Outlier("Charcoal",1);

};

R_Date("Beta-437271",2550,30)

{

Outlier("General",0.05);

};

R_Date("ANU-13013",2540,30)

{

Outlier("Charcoal",1);

};

R_Date("Wk-17756",2528,31)

{

Outlier("Charcoal",1);

};

R_Date("GX-26711-AMS",2520,50)

{

Outlier("Charcoal",1);

};

R_Date("Wk-19712",2504,35)

{

Outlier("General",0.05);

};

R_Date("Wk-18059",1946,30)

{

Outlier("Charcoal",1);

};

};

Boundary("end lower silt");

Boundary("start shell midden");

Phase("shell midden")

{

R_Date("ANU-13017",3420,30)

{

Outlier("General",0.05);

};

R_Date("ANU-13024",2680,30)

{

Outlier("General",0.05);

};

R_Date("ANU-13020",2620,30)

{

Outlier("General",0.05);

};

R_Date("ANU-13019",2560,30)

{

Outlier("General",0.05);

};

R_Date("GX-26801",2260,270)

{

Outlier("Charcoal",1);

};

R_Date("GX-26802",2240,270)

{

Outlier("Charcoal",1);

};

R_Date("GX-26806",2150,150)

{

Outlier("Charcoal",1);

};

R_Date("GX-26705",2120,220)

{

Outlier("Charcoal",1);

};

R_Date("GX-26799",1960,90)

{

Outlier("Charcoal",1);

};

R_Date("GX-26699",1920,80)

{

Outlier("Charcoal",1);

};

R_Date("GX-26698",1830,70)

{

Outlier("Charcoal",1);

};

R_Date("GX-26702-AMS",1820,40)

{

Outlier("Charcoal",1);

};

R_Date("GX-26800",1760,110)

{

Outlier("Charcoal",1);

};

R_Date("GX-26798",1670,60)

{

Outlier("Charcoal",1);

};

R_Date("GX-26797",1470,50)

{

Outlier("Charcoal",1);

};

};

Boundary("end");

};

};

Nagsabaran, Two-Phase, Six Oldest Determinations Excluded [Preferred Model]

Options()

{

kIterations=300;

};

Plot()

{

Outlier_Model("General",T(5),U(0,2.3),"t");

Outlier_Model("Charcoal", Exp(1,-10,0), U(0,2.3), "t");

Sequence("Luzon_Nagsabaran")

{

Boundary("start");

Phase("Lower silt")

{

R_Date("Wk-19713",4450,39)

{

Outlier("Charcoal",1);

};

R_Date("Wk-23997",3940,40)

{

Outlier("General",0.05);

};

R_Date("Beta-436818",3760,30)

{

Outlier("Charcoal",1);

};

R_Date("ANU-13016",3510,30)

{

Outlier("Charcoal",1);

};

R_Date("NTU-3799",3450,40)

{

Outlier("General",0.05);

};

R_Date("GX-28381",3390,130)

{

Outlier("Charcoal",1);

};

R_Date("GX-28379",3050,70)

{

Outlier("Charcoal",1);

};

R_Date("NTU-3798",2670,40)

{

Outlier("Charcoal",1);

};

R_Date("ANU-13014",2660,30)

{

Outlier("Charcoal",1);

};

R_Date("GX-26704-AMS",2620,40)

{

Outlier("Charcoal",1);

};

R_Date("Beta-437271",2550,30)

{

Outlier("General",0.05);

};

R_Date("ANU-13013",2540,30)

{

Outlier("Charcoal",1);

};

R_Date("Wk-17756",2528,31)

{

Outlier("Charcoal",1);

};

R_Date("GX-26711-AMS",2520,50)

{

Outlier("Charcoal",1);

};

R_Date("Wk-19712",2504,35)

{

Outlier("General",0.05);

};

R_Date("Wk-18059",1946,30)

{

Outlier("Charcoal",1);

};

};

Boundary("end lower silt");

Boundary("start shell midden");

Phase("shell midden")

{

R_Date("GX-26801",2260,270)

{

Outlier("Charcoal",1);

};

R_Date("GX-26802",2240,270)

{

Outlier("Charcoal",1);

};

R_Date("GX-26806",2150,150)

{

Outlier("Charcoal",1);

};

R_Date("GX-26705",2120,220)

{

Outlier("Charcoal",1);

};

R_Date("GX-26799",1960,90)

{

Outlier("Charcoal",1);

};

R_Date("GX-26699",1920,80)

{

Outlier("Charcoal",1);

};

R_Date("GX-26698",1830,70)

{

Outlier("Charcoal",1);

};

R_Date("GX-26702-AMS",1820,40)

{

Outlier("Charcoal",1);

};

R_Date("GX-26800",1760,110)

{

Outlier("Charcoal",1);

};

R_Date("GX-26798",1670,60)

{

Outlier("Charcoal",1);

};

R_Date("GX-26797",1470,50)

{

Outlier("Charcoal",1);

};

};

Boundary("end");

};

};

Nagsabaran, Two-Phase, Hung et al. (2011) Preferred Determinations

Options()

{

kIterations=100;

};

Plot()

{

Outlier_Model("General",T(5),U(0,2.3),"t");

Outlier_Model("Charcoal", Exp(1,-10,0), U(0,2.3), "t");

Sequence("Luzon_Nagsabaran")

{

Boundary("start");

Phase("Lower silts")

{

R_Date("Wk-23997",3940,40)

{

Outlier("General",0.05);

};

R_Date("Beta-436818",3760,30)

{

Outlier("Charcoal",1);

};

R_Date("ANU-13016",3510,30)

{

Outlier("Charcoal",1);

};

R_Date("NTU-3799",3450,40)

{

Outlier("General",0.05);

};

R_Date("GX-28381",3390,130)

{

Outlier("Charcoal",1);

};

R_Date("GX-28379",3050,70)

{

Outlier("Charcoal",1);

};

R_Date("Beta-437271",2550,30)

{

Outlier("General",0.05);

};

R_Date("GX-26711-AMS",2520,50)

{

Outlier("Charcoal",1);

};

R_Date("Wk-19712",2504,35)

{

Outlier("General",0.05);

};

};

Boundary("end lower silts");

Boundary("start shell midden");

Phase("shell midden")

{

R_Date("ANU-13024",2680,30)

{

Outlier("General",0.05);

};

R_Date("ANU-13020",2620,30)

{

Outlier("General",0.05);

};

R_Date("ANU-13019",2560,30)

{

Outlier("General",0.05);

};

R_Date("GX-26801",2260,270)

{

Outlier("Charcoal",1);

};

R_Date("GX-26802",2240,270)

{

Outlier("Charcoal",1);

};

R_Date("GX-26806",2150,150)

{

Outlier("Charcoal",1);

};

R_Date("GX-26705",2120,220)

{

Outlier("Charcoal",1);

};

R_Date("GX-26799",1960,90)

{

Outlier("Charcoal",1);

};

R_Date("GX-26699",1920,80)

{

Outlier("Charcoal",1);

};

R_Date("GX-26698",1830,70)

{

Outlier("Charcoal",1);

};

R_Date("GX-26702-AMS",1820,40)

{

Outlier("Charcoal",1);

};

R_Date("GX-26800",1760,110)

{

Outlier("Charcoal",1);

};

R_Date("GX-26798",1670,60)

{

Outlier("Charcoal",1);

};

R_Date("GX-26797",1470,50)

{

Outlier("Charcoal",1);

};

};

Boundary("end");

};

};

**Luzon Island**

The 16 determinations from the silt layers phase of the preferred Nagsabaran model were combined with eight ages from Andarayan, Irigayen, Callao, and Dalan Serkot in a single-phase island-wide model for Luzon. The model has radiocarbon determinations derived from unidentified charcoal, rice grain charcoal, a pig tooth, an animal bone, and freshwater shell. At this scale, the appearance of pottery occurred no earlier than *5430-4290 cal BP (95.4%)* or *5280-4370 cal BP (68.2%)*.

Plot()

{

Outlier_Model("General",T(5),U(0,2.3),"t");

Outlier_Model("Charcoal", Exp(1,-10,0), U(0,2.3), "t");

Sequence("Luzon")

{

Boundary("start");

Phase("Luzon")

{

Phase("Nagsabaran")

{

R_Date("Wk-19713",4450,39)

{

Outlier("Charcoal",1);

};

R_Date("Wk-23997",3940,40)

{

Outlier("General",0.05);

};

R_Date("Beta-436818",3760,30)

{

Outlier("Charcoal",1);

};

R_Date("ANU-13016",3510,30)

{

Outlier("Charcoal",1);

};

R_Date("NTU-3799",3450,40)

{

Outlier("General",0.05);

};

R_Date("GX-28381",3390,130)

{

Outlier("Charcoal",1);

};

R_Date("GX-28379",3050,70)

{

Outlier("Charcoal",1);

};

R_Date("NTU-3798",2670,40)

{

Outlier("Charcoal",1);

};

R_Date("ANU-13014",2660,30)

{

Outlier("Charcoal",1);

};

R_Date("GX-26704-AMS",2620,40)

{

Outlier("Charcoal",1);

};

R_Date("Beta-437271",2550,30)

{

Outlier("General",0.05);

};

R_Date("ANU-13013",2540,30)

{

Outlier("Charcoal",1);

};

R_Date("Wk-17756",2528,31)

{

Outlier("Charcoal",1);

};

R_Date("GX-26711-AMS",2520,50)

{

Outlier("Charcoal",1);

};

R_Date("Wk-19712",2504,35)

{

Outlier("General",0.05);

};

R_Date("Wk-18059",1946,30)

{

Outlier("Charcoal",1);

};

};

Phase("Dalan Serkot")

{

R_Date("Wk-15648",3530,34)

{

Outlier("Charcoal",1);

};

};

Phase("Callao Cave")

{

R_Date("Wk-17010",3335,34)

{

Outlier("Charcoal",1);

};

};

Phase("Irigayen")

{

R_Date("NUTA2-917",3185,25)

{

Outlier("Charcoal",1);

};

R_Date("NUTA2-913",3165,25)

{

Outlier("Charcoal",1);

};

R_Date("NUTA2-914",3025,20)

{

Outlier("Charcoal",1);

};

R_Date("NUTA2-912",2925,20)

{

Outlier("Charcoal",1);

};

};

Phase("Andarayan")

{

R_Date("Unreported",3400,125)

{

Outlier("General",0.05);

};

R_Date("SFU-86",3240,160)

{

Outlier("Charcoal",1);

};

};

};

Boundary("end");

};

};

**Greater Sunda Islands**

Six single- or multi-phase models were created for site deposits and islands within the Greater Sunda Islands: Borneo[28-31] and Sulawesi [32-36]. Determinations from these sites and four other site deposits or excavation areas that produced individual radiocarbon ages (Braholo Cave [37], Keplek Cave [37], Mansiri [38], and Minango Sipakko, Test Pit 2 [36]) were included in single-phase models for each respective island. Two determinations from Topogaro 2 Cave in eastern Sulawesi [39] were not used as it is unclear which determination, or if both, are most clearly associated with the ceramics.

In consideration of the large size of Borneo and the likelihood that the initial appearance of ceramics varied spatially through time, a single-phase model was also run for the northern portion of the island (other areas of the island are only represented by individual sites) (S3 Fig).

Dating samples were unidentified wood charcoal, freshwater shell, human bone, and a rice husk. For the one site that has a determination from freshwater shell, models were created with and without this age. Lacking reservoir correction values, this was done to evaluate the effect that the shell-derived determinations have on dates. The age obtained from the human bone specimen at Niah Cave (unreported laboratory number, 4990±90 BP) [40] is excluded since isotopic data is not reported and therefore it is unknown whether a mixed atmospheric and marine calibration is warranted. A single determination from Jambu Hilir (Wk-22009, 2922±45 BP) was not used in the models as pottery appears in contexts up to 90 cm below the dated sample [41]. It is not clear, therefore, that the dated sample is associated with earliest ceramic deposition.

**MAD1 Site, Borneo**

Two multi-phase models were created for the MAD1 site. One contains five radiocarbon determinations obtained from unidentified charcoal, which are organized in a contiguous sequence based on excavation level. The oldest determinations are from a pre-ceramic level (Level 12), with Level 11a being the deepest ceramic deposit, which is capped by another ceramic-bearing deposit (Layer 10). The boundary between the phases for Levels 12 and 11a (“transition Layer 12/Layer 11a”) is the event of interest. The other model has the same structure but includes a single freshwater shell determination (ANU-2943, 2700±70 BP) in the post-initial ceramic phase.

The first model dates that the initial appearance of ceramics at the MAD1 site occurred sometime between *8580-2710 cal BP (95.4%) or 8530-2730 cal BP (68.2%)*. The second model dates that this event occurred sometime between *8570-2730 cal BP (95.4%) or 8490-2750 cal BP (68.2%)*. The difference between these dates is minor. However, due to the uncertainty introduced by the freshwater shell determination, our preferred iteration is the charcoal-only model.

MAD1 Site without shell determination [Preferred model]

Plot()

{

Outlier_Model("Charcoal", Exp(1,-10,0), U(0,2.3), "t");

Sequence("Sabah MAD1")

{

Boundary("start");

Phase("Layer 12, preceramic")

{

R_Date("ANU-2397",7920,370)

{

Outlier(1);

};

R_Date("ANU-2398",7390,270)

{

Outlier(1);

};

};

Boundary("transition Layer 12/Layer 11a");

Phase("Layer 11a")

{

R_Date("ANU-2396",2650,80)

{

Outlier(1);

};

};

Boundary("transition Layer 11a/Layer 10");

Phase("Layer 10")

{

R_Date("ANU-2945",2020,90)

{

Outlier(1);

};

R_Date("ANU-2395",1590,150)

{

Outlier(1);

};

};

Boundary("end");

};

};

MAD1 Site with shell determination

Options()

{

kIterations=100;

};

Plot()

{

Outlier_Model("General",T(5),U(0,2.3),"t");

Outlier_Model("Charcoal", Exp(1,-10,0), U(0,2.3), "t");

Sequence("Sabah MAD1")

{

Boundary("start");

Phase("Layer 12, preceramic")

{

R_Date("ANU-2397",7920,370)

{

Outlier("Charcoal",1);

};

R_Date("ANU-2398",7390,270)

{

Outlier("Charcoal",1);

};

};

Boundary("transition Layer 12/Layer 11a");

Phase("Layer 11a")

{

R_Date("ANU-2396",2650,80)

{

Outlier("Charcoal",1);

};

};

Boundary("transition Layer 11a/Layer 10");

Phase("Layer 10")

{

R_Date("ANU-2945",2020,90)

{

Outlier("Charcoal",1);

};

R_Date("ANU-2395",1590,150)

{

Outlier("Charcoal",1);

};

R_Date("ANU-2943",2700,70)

{

Outlier("General",0.05);

};

};

Boundary("end");

};

};

**Borneo Island**

The charcoal and rice husk determinations from the basal ceramic-bearing deposits at MAD1, Liang Abu, Bukit Tengkorak, and Gua Sireh were combined in a single-phase island-wide model for Borneo. The freshwater shell determination from MAD1 was excluded since it did not significantly affect the site-scale model but also still introduces a degree of uncertainty regarding the need for localized correction factors. This model was run 12 times, seven of which had one model parameter with a convergence value <95%; the date presented here is from a model run that had sufficient convergence values. At this geographic scale, the appearance of pottery occurred sometime between *6680-3820 cal BP (95.4%) or 5230-4170 cal BP (68.2%)*. These dates are associated with red-slip pottery, along with cord-mark, incised, impressed, and circle-stamp surface treatments at some sites [28-31].

Options()

{

kIterations=3000;

};

Plot()

{

Outlier_Model("General",T(5),U(0,2.3),"t");

Outlier_Model("Charcoal", Exp(1,-10,0), U(0,2.3), "t");

Sequence("Borneo_Island")

{

Boundary("start");

Phase("Borneo")

{

R_Date("ANU-7049",3990,230)

{

Outlier("Charcoal"1);

};

R_Date("CAMS-725",3850,260)

{

Outlier("General",0.05);

};

R_Date("ANU-5769",2700,110)

{

Outlier("Charcoal"1);

};

R_Date("ANU-2396",2650,80)

{

Outlier("Charcoal"1);

};

R_Date("ANU-5770",2330,170)

{

Outlier("Charcoal"1);

};

R_Date("UBA-20839",1672,21)

{

Outlier("Charcoal"1);

};

R_Date("UBA-20840",1524,22)

{

Outlier("Charcoal"1);

};

};

Boundary("end");

};

};

**Northern Borneo**

Since there may have been spatial variation in the timing of the adoption of pottery across Borneo, a single-phase model was created for northern Borneo that consists of the determinations from Liang Abu, Bukit Tengkorak, and MAD1. For this portion of the island, the appearance of pottery occurred sometime between *4560-2460 cal BP (95.4%) or 3360-2730 cal BP (68.2%)*.

Plot()

{

Outlier_Model("Charcoal", Exp(1,-10,0), U(0,2.3), "t");

Sequence("Borneo_Island")

{

Boundary("start");

Phase("North Borneo")

{

R_Date("ANU-5769",2700,110)

{

Outlier(1);

};

R_Date("ANU-2396",2650,80)

{

Outlier(1);

};

R_Date("ANU-5770",2330,170)

{

Outlier(1);

};

R_Date("UBA-20839",1672,21)

{

Outlier(1);

};

R_Date("UBA-20840",1524,22)

{

Outlier(1);

};

};

Boundary("end");

};

};

**Minango Sipakko Site, Sulawesi Island**

One model provided results for the Minango Sipakko site on Sulawesi Island. A single-phase, site-scale model was created with a single determination from Unit M1 and five determinations from Layer 2, the basal ceramic deposit within Units M3, M4, and M5 (these latter three excavation units were contiguous forming a larger trench excavation). An initial model with the oldest radiocarbon determination from Unit M5 (P3G-05, 4950±180 BP, 260-270 cm bs) had low convergence values for some model parameters, so this determination was excluded. All determinations are from unidentified charcoal.

The single-phase model for the site as a whole produces a date for the onset of ceramics sometime between *4600-3510 cal BP (95.4%) or 4120-3640 cal BP (68.2%)*.

Plot()

{

Outlier_Model("Charcoal", Exp(1,-10,0), U(0,2.3), "t");

Sequence("Sulawesi Minanga Sipakko")

{

Boundary("start");

Phase("Minanga Sipakko")

{

R_Date("P3G-05",3690,160)

{

Outlier(1);

};

R_Date("Wk-14651",3446,54)

{

Outlier(1);

};

R_Date("Wk-17981",3343,46)

{

Outlier(1);

};

R_Date("Wk-14652",3082,50)

{

Outlier(1);

};

R_Date("Wk-14654",2996,41)

{

Outlier(1);

};

R_Date("Wk-14653",2881,46)

{

Outlier(1);

};

};

Boundary("end");

};

};

**Sulawesi Island**

The six determinations from Minango Sipakko were combined with two determinations from Malawa and an individual age from Mansiri in a single-phase, island-wide model for Sulawesi. Freshwater and estuarine shell-derived radiocarbon determinations from Kamassi were excluded due to the potential that a correction value is required. At this geographic scale, the appearance of pottery occurred sometime between *4550-3590 cal BP (95.4%) or 4180-3740 cal BP (68.2%)*. The early Sulawesi ceramic deposits contain red-slip, impressed, incised, or circle-stamped sherds [32, 34, 36, 42]

Plot()

{

Outlier_Model("Charcoal", Exp(1,-10,0), U(0,2.3), "t");

Sequence("Sulawesi Island")

{

Boundary("start");

Phase("Sulawesi")

{

R_Date("P3G-05",3690,160)

{

Outlier(1);

};

R_Date("P3G-06",3580,130)

{

Outlier(1);

};

R_Date("Wk-14651",3446,54)

{

Outlier(1);

};

R_Date("Wk-17981",3343,46)

{

Outlier(1);

};

R_Date("Wk-14652",3082,50)

{

Outlier(1);

};

R_Date("Wk-14654",2996,41)

{

Outlier(1);

};

R_Date("Wk-14653",2881,46)

{

Outlier(1);

};

R_Date("P3G-06",2710,170)

{

Outlier(1);

};

R_Date("Wk-44610",2469,20)

{

Outlier(1);

};

};

Boundary("end");

};

};

**Lesser Sunda Islands**

A single-phase model was created for the Pain Haka site deposit on Flores Island [43] in the Lesser Sunda Islands (S3 Fig). Dating samples were unidentified charcoal and human bone. Human bone determinations were calibrated using either IntCal20 or a mixed marine-atmospheric curve.

**Pain Haka Site, Flores**

The single-phase model for Pain Haka, Flores Island is also the island-scale model. Dating samples were unidentified charcoal and human bone from burials associated with ceramic vessels. The contexts for the charcoal, with one exception (Wk-36712), are unreported, but presumably originate from the archaeological deposit(s) associated with the burials. Galipaud et al. ([43, supplementary material]) note that the nitrogen and carbon stable isotope ratios of the human bone samples possibly indicate the consumption of C_4_ plants or low trophic level marine foods. Because of this ambiguity in past diet, they calibrate the bone-derived determinations with the northern hemisphere atmospheric curve. In consideration of the isotopic data, we estimated the percent marine dietary component for the six human bone-derived determinations based on δ^13^C‰ values [44, 45] and used a mixed marine-atmospheric calibration; the marine percentages are assigned ±10% error. No marine offset was used since no ΔR value has been determined for the site locale, Flores Island, or general area; we acknowledge that this may introduce a degree of inaccuracy.

The initial appearance of ceramics at the Pain Haka site on Flores Island occurred sometime between *3200-2600 cal BP (95.4%) or 3010-2750 cal BP (68.2%)*. The Pain Haka ceramics include red-slip, incised, and applique surface treatments [43].

Plot()

{

Outlier_Model("General",T(5),U(0,2.3),"t");

Outlier_Model("Charcoal", Exp(1,-10,0), U(0,2.3), "t");

Sequence("Pain Haka")

{

Boundary("start");

Phase("Pain Haka")

{

R_Date("Wk-36712",2784,25)

{

Outlier("Charcoal",1);

};

R_Date("Wk-28997",2725,25)

{

Outlier("Charcoal",1);

};

R_Date("Wk-28996",2535,25)

{

Outlier("Charcoal",1);

};

R_Date("Wk-28995",2509,25)

{

Outlier("Charcoal",1);

};

Curve("IntCal20","intcal20.14c");

Curve("Marine20","marine20.14c");

Mix_Curve("Mixed","IntCal20","Marine20",53,10);

R_Date("Wk-36556",2831,25)

{

Outlier("General",0.05);

};

Curve("IntCal20","intcal20.14c");

Curve("Marine20","marine20.14c");

Mix_Curve("Mixed","IntCal20","Marine20",80,10);

R_Date("Wk-36558",2588,25)

{

Outlier("General",0.05);

};

Curve("IntCal20","intcal20.14c");

Curve("Marine20","marine20.14c");

Mix_Curve("Mixed","IntCal20","Marine20",75,10);

R_Date("Wk-36557",2570,25)

{

Outlier("General",0.05);

};

Curve("IntCal20","intcal20.14c");

Curve("Marine20","marine20.14c");

Mix_Curve("Mixed","IntCal20","Marine20",46,10);

R_Date("Wk-36559",2548,25)

{

Outlier("General",0.05);

};

Curve("IntCal20","intcal20.14c");

Curve("Marine20","marine20.14c");

Mix_Curve("Mixed","IntCal20","Marine20",59,10);

R_Date("Wk-41599",2532,20)

{

Outlier("General",0.05);

};

Curve("IntCal20","intcal20.14c");

Curve("Marine20","marine20.14c");

Mix_Curve("Mixed","IntCal20","Marine20",64,10);

R_Date("Wk-36560",2246,25)

{

Outlier("General",0.05);

};

};

Boundary("end");

};

};

**Molucca Islands**

A single multi-phase model was created for site deposits at PA1, Pulau Ay [46] (S3 Fig). The site model is also the island-scale model. Um Kapat Papo on Gebe Island and Uattamdi on Kayoa Island [47] were excluded from analysis as these sites have too few radiocarbon determinations. Tanjung Pinang on Morotai and Siti Nafisah on Halmahera are excluded from analysis as it is unclear which radiocarbon determinations relate to the pottery-bearing portions of the archaeological deposits [47:237-242, 253-254]. Two determinations from the LTM site in the Talaud Islands [48] are also excluded from modeling because they are from different excavation units and are not stratigraphically correlated. All of these determinations, while not used in our analyses, appear in S3 Table.

**PA1, Pulau Ay**

The initial appearance of ceramics at the PA1 site on Pulau Ay occurred sometime between *3740-3020 cal BP (95.4%) or 3510-3210 cal BP (68.2%)*. The two-phase model contains seven radiocarbon determinations obtained from unidentified charcoal. Two radiocarbon determinations are from the basal ceramic-bearing deposit (Layer 6) with five determinations from two overlying strata (Layers 3 and 4), which are separated from the basal deposit by a culturally sterile pumice layer. All determinations are from unidentified charcoal. The assemblage contains red-slip and incised sherds, with circle-stamp appearing in more recent deposits [46].

Plot()

{

Outlier_Model("Charcoal", Exp(1,-10,0), U(0,2.3), "t");

Sequence("Pulau Ay PA1")

{

Boundary("start");

Phase("Layer 6")

{

R_Date("240739",3190,40)

{

Outlier(1);

};

R_Date("Beta-235453",3010,40)

{

Outlier(1);

};

};

Boundary("end Layer 6");

Boundary("start post-Layer 6 strata");

Phase("Layers 3 & 4")

{

R_Date("Beta-240738",3010,40)

{

Outlier(1);

};

R_Date("Beta-304478",2940,40)

{

Outlier(1);

};

R_Date("Beta-302405",2880,30)

{

Outlier(1);

};

R_Date("Beta-302404",2780,30)

{

Outlier(1);

};

R_Date("Beta-235454",2460,40)

{

Outlier(1);

};

};

Boundary("end post-Layer 6 strata");

};

};

**Mussau Islands**

Two single-phase models were created for site deposits on Eloaua (S4 Fig), and determinations from Eloaua sites ECA and ECB were combined in a single-phase, island-scale model for that island. Dating samples were identified and unidentified wood, charcoal, and bark; coconut (*Cocos nucifera*) endocarp; and identified marine shell. The early ceramics from the Mussau islands comprise red-slip, incised, circle-stamp, and dentate surface treatments [49, 50].

**ECA Site, Eloaua Island**

The initial appearance of ceramics at ECA occurred sometime between *3460-3010 cal BP (95.4%) or 3330-3080 cal BP (68.2%)*. The single-phase model contains 20 radiocarbon determinations obtained from unidentified charcoal (n = 6), identified wood or charcoal (n = 3), coconut endocarp (n =1) and marine shell of various taxa (n = 10).

Options()

{

kIterations=100;

};

Plot()

{

Outlier_Model("General",T(5),U(0,2.3),"t");

Outlier_Model("Charcoal", Exp(1,-10,0), U(0,2.3), "t");

Sequence("ECA")

{

Boundary("start ECA");

Phase("Mussau")

{

R_Date("ANU-5080", 3260, 90)

{

Outlier(1);

};

R_Date("Beta-30684", 3100, 110)

{

Outlier(1);

};

R_Date("Beta-20452", 3050, 70)

{

Outlier(1);

};

R_Date("Beta-30682", 2970, 50)

{

Outlier(1);

};

R_Date("ANU-5790", 2950, 80)

{

Outlier(1);

};

R_Date("Beta-20451", 2950, 70)

{

Outlier("General",0.05);

};

R_Date("ANU-5791", 2930, 80)

{

Outlier(1);

};

R_Date("Beta-30681", 2860, 60)

{

Outlier(1);

};

R_Date("Beta-30686", 2850, 70)

{

Outlier(1);

};

Curve("Marine20","marine20.14c");

Delta_R("LocalMarine", -434,179);

R_Date("Beta-30680", 3320, 80)

{

Outlier("General",0.05);

};

R_Date("ANU-5084", 3190, 80)

{

Outlier("General",0.05);

};

R_Date("Beta-30678", 3190, 80)

{

Outlier("General",0.05);

};

R_Date("Beta-30677", 3170, 70)

{

Outlier("General",0.05);

};

R_Date("Beta-30683", 3140, 80)

{

Outlier("General",0.05);

};

R_Date("ANU-5085", 3130, 80)

{

Outlier("General",0.05);

};

R_Date("Beta-30674", 3110, 70)

{

Outlier("General",0.05);

};

R_Date("Beta-30675", 3110, 80)

{

Outlier("General",0.05);

};

R_Date("Beta-30679", 3080, 70)

{

Outlier("General",0.05);

};

R_Date("ANU-5081", 3010, 80)

{

Outlier("General",0.05);

};

R_Date("ANU-5082", 2950, 80)

{

Outlier("General",0.05);

};

};

Boundary("end ECA");

};

};

**Eloaua Island**

The 20 determinations from the ECA model were combined with three determinations from ECB in a single-phase, island-wide model for Eloaua. At this geographic scale, the appearance of pottery occurred sometime between *3470-3010 cal BP (95.4%) or 3350-3070 cal BP (68.2%)*.

Options()

{

kIterations=100;

};

Plot()

{

Outlier_Model("General",T(5),U(0,2.3),"t");

Outlier_Model("Charcoal", Exp(1,-10,0), U(0,2.3), "t");

Sequence("Eloaua")

{

Boundary("start Eloaua");

Phase("Eloaua")

{

R_Date("ANU-5080", 3260, 90)

{

Outlier(1);

};

R_Date("Beta-20453",3200,70)

{

Outlier(1);

};

R_Date("Beta-30684", 3100, 110)

{

Outlier(1);

};

R_Date("Beta-20452", 3050, 70)

{

Outlier(1);

};

R_Date("Beta-30682", 2970, 50)

{

Outlier(1);

};

R_Date("ANU-5790", 2950, 80)

{

Outlier(1);

};

R_Date("Beta-20451", 2950, 70)

{

Outlier("General",0.05);

};

R_Date("ANU-5791", 2930, 80)

{

Outlier(1);

};

R_Date("Beta-30681", 2860, 60)

{

Outlier(1);

};

R_Date("Beta-30686", 2850, 70)

{

Outlier(1);

};

Curve("Marine20","Marine20.14c");

Delta_R("LocalMarine", -434,179);

R_Date("Beta-30680", 3320, 80)

{

Outlier("General",0.05);

};

R_Date("ANU-5084", 3190, 80)

{

Outlier("General",0.05);

};

R_Date("Beta-30678", 3190, 80)

{

Outlier("General",0.05);

};

R_Date("Beta-30677", 3170, 70)

{

Outlier("General",0.05);

};

R_Date("ANU-5087",3150,80)

{

Outlier("General",0.05);

};

R_Date("Beta-30683", 3140, 80)

{

Outlier("General",0.05);

};

R_Date("ANU-5085", 3130, 80)

{

Outlier("General",0.05);

};

R_Date("ANU-5086",3120,80)

{

Outlier("General",0.05);

};

R_Date("Beta-30674", 3110, 70)

{

Outlier("General",0.05);

};

R_Date("Beta-30675", 3110, 80)

{

Outlier("General",0.05);

};

R_Date("Beta-30679", 3080, 70)

{

Outlier("General",0.05);

};

R_Date("ANU-5081", 3010, 80)

{

Outlier("General",0.05);

};

R_Date("ANU-5082", 2950, 80)

{

Outlier("General",0.05);

};

};

Boundary("end Eloaua");

};

};

**References**

1. Bellwood P, Dizon E. The Batanes Archaeological Project and the "Out of Taiwan" Hypothesis for Austronesian Dispersal. Journal of Austronesian Studies. 2005;1(1):1-33.

2. Bellwood P, Dizon E. The Chronology of Batanes Prehistory. In: Bellwood P, Dizon E, editors. 4000 Years of Migration and Cultural Exchange. Canberra: The Australian National University; 2013. p. 68-75.

3. Bellwood P, Anderson A, Dizon E. Archaeological Excavations on Batan Island. In: Bellwood P, Dizon E, editors. 4000 Years of Migration and Cultural Exchange. terra australis. 40. Canberra: The Australian National University; 2013. p. 31-45.

4. Bellwood P, Stevenson J, Anderson A, Dizon E. Archaeological and palaeoenvironmental research in Batanes and Ilocos Norte Provinces, northern Philippines. Bulletin of the Indo-Pacific Prehistory Association. 2003;23:141-62.

5. Hung H-c. Migration and Cultural Interaction in Southern Coastal China, Taiwan and the Northern Philippines, 3000 BC to AD 100: The Early History of the Austronesian speaking Populations [Thesis]: The Australian National University; 2008.

6. Bellwood P, Dizon E, Mijares A. Archaeological Excavations on Itbayat and Siayan Islands. In: Bellwood P, Dizon E, editors. 4000 Years of Migration and Cultural Exchange: The Archaeology of the Batanes Islands, Northern Philippines2013. p. 9-29.

7. Casanova E, Knowles TDJ, Bayliss A, Dunne J, Barański MZ, Denaire A, et al. Accurate compound-specific 14C dating of archaeological pottery vessels. Nature. 2020;580(7804):506-10. doi: 10.1038/s41586-020-2178-z.

8. Richard M, Maury R, Bellon H, Stephan J, Boirat J, Caldero A. Geology of Mt Iraya and Batan Island, northern Philippines. Philippine Journal of Volcanology. 1986;3(1):1-27.

9. Bellwood P, Dizon E, De Leon A. The Batanes Pottery Sequence, 2500 BC to Recent. In: Bellwood P, Dizon E, editors. 4000 Years of Migration and Cultural Exchange: The Archaeology of the Batanes Islands, Northern Philippines. Terra Australis. 40. Canberra: The Australia National University; 2013. p. 77-113.

10. Amano N. The faunal remains of Nagsabaran in Cagayan, Northern Philippines: Subsistence strategies in the Late Holocene [Thesis]: University of the Philippines; 2011.

11. Hung H-C, Carson MT, Bellwood P, Campos FZ, Piper PJ, Dizon E, et al. The first settlement of Remote Oceania: the Philippines to the Marianas: supplementary information on the radiocarbon dating of the Nagsabaran site. Antiquity. 2011;85:909-26.

12. Carson MT, Hung H-c. Learning from Paleo-Landscapes: Defining the Land-Use Systems of the Ancient Malayo-Polynesian Homeland. Current Anthropology. 2018;59:790-813. doi: 10.1086/700757.

13. Snow BE, Shutler R, Nelson DE, Vogel JS, Southon JR. Evidence of Early Rice Cultivation in the Philippines. Philippine Quarterly of Culture and Society. 1986;14(1):3-11.

14. Ogawa H. Typological Crhonology of Pottery Assemblages from Lal-lo Shell Middens in Northern Luzon, Philippines. Journal of Southeast Asian Archaeology. 2005;25:1-29.

15. Mijares ASB. The Archaeology of Peñablanca Cave Sits, Northern Luzon, Philippines. Journal of Austronesian Studies. 2005;1(2):65-93.

16. Bay-Petersen J. Excavations at Bagumbayan, Masbate, central Philippines: an economic analysis. Asian Perspectives. 1982;25(2):67-98.

17. Hung H-C, Carson MT, Bellwood P, Campos FZ, Piper PJ, Dizon E, et al. The first settlement of Remote Oceania: the Philippines to the Marianas. Antiquity. 2011;85:909-26.

18. Hutterer K, L. Test Excavations at the Edjek Site (TV161A) Tanjay, Negors Oriental. In: Hutterer K, L, Macdonald W, K, editors. Houses Built on Scattered Poles: Prehistory and Ecology in Negros, Oriental, Philippines. Cebu City: University of San Carlos; 1982. p. 209-26.

19. Piper PJ, Campos FZ, Hung H-c. A study of the animal bone recovered from Pits 9 and 10 at the site of Nagsabaran in northern Luzon, Philippines. Hukay. 2009;14.

20. Piper PJ, Hung H-c, Campos FZ, Bellwood P, Santiago R. A 4000 year-old introduction of domestic pigs into the Philippine Archipelago: implications for understanding routes of human migration through Island Southeast Asia and Wallacea. Antiquity. 2009;83(321):687-95.

21. Solheim WG. The Batungan cave sites, Masbate, Philippines. Asian and Pacific Archaeology Series. 1968;2:21-62.

22. Thiel B. Excavations at the Lal-lo shellmiddens, northeast Luzon, Philippines. Asian Perspectives. 1986;27(1):71-94.

23. Tanaka K, Orogo AB. The archaeological excavation at the Pamittan site, Barangay Lanna, Solana, Cagayan Province, Philippines. 環境情報研究. 2000;(8):113-41.

24. Spriggs M. Chronology of the Neolithic transition in Island Southeast Asia and the Western Pacific: a view from 2003. The Review of Archaeology. 2003;24(2):57-80.

25. Petchey F, Clark G, Lindeman I, O'Day P, Southon J, Dabell K, et al. Forgotten news: Shellfish isotopic insight into changing sea-level and associated impact on the first settlers of the Mariana Archipelago. Quaternary Geochronology. 2018;48:180-94. doi: https://doi.org/10.1016/j.quageo.2018.10.002.

26. Amano N. The Faunal remains of Nagsabaran in Cagayan, Northern Philippines: Subsistence Strategies in the Late Holocene. Dilman: University of the Philippines; 2011.

27. Carson MT, Hung H-c, Summerhayes GR, Bellwood P. The Pottery Trail From Southeast Asia to Remote Oceania. The Journal of Island and Coastal Archaeology. 2013;8(1):17-36. doi: 10.1080/15564894.2012.726941.

28. Bellwood P, editor. Archaeological Research in South-Eastern Sabah. Kota Kinabalu, Indonesia: Sabah Museum & Archives; 1988.

29. Bellwood P. Archaeological Investigations at Bukit Tengkorak and Segarong, Southeastern Sabah. IPPA Bulletin. 1989;9:122-62.

30. Datan I, Bellwood P. Recent Research at Gua Sireh (Serian) and Lubang Angin (Gunung Mulu National Park), Sarawak. Indo-Pacific Prehistory Association Bulletin. 1991;10:386-405.

31. Plutniak S, Oktaviana AA, Sugiyanto B, Chazine JM, Ricaut FX. New ceramic data from East Kalimantan: Pottery chronology and the cord-marked and red-slipped sherds of Liang Abu’s Layer 2. Journal of Pacific Archaeology. 2014;5(1):90-9.

32. Anggraeni. The Austronesian Migration Hypothesis as Seen from Prehistoric Settlements on the Karama River, Mamuju, West Sulawesi [Thesis]: The Australian National University; 2012.

33. Anggraeni, Simanjuntak T, Bellwood P, Piper P. Neolithic foundations in the Karama valley, West Sulawesi, Indonesia. Antiquity. 2014;88(341):740-56. Epub 2014/08/26. doi: 10.1017/S0003598X00050663.

34. Mahmud I. The Neolithic Site of Mallawa. In: Simanjuntak T, editor. Austronesian in Sulawesi. Yogyakarta, Indonesia: Center for Prehistoric and Austronesian Studies; 2008. p. 119-28.

35. Simanjuntak T, editor. Austronesian in Sulawesi. Indonesia: Center for Prehistoric and Austronesian Studies; 2008.

36. Simanjuntak T, Morwood MJ, Intan FS, Machmud I, Grant K, Somba N, et al. Minanga Sipakko and the Neolithic of the Karama River. In: Simanjuntak T, editor. Austronesian in Sulawesi. Indonesia: Center for Prehistoric and Austronesian Studies; 2008. p. 57-76.

37. Simanjuntak T. New insight on the prehistoric chronology of Gunung Sewu, Java, Indonesia. Modern quaternary research in Southeast Asia. 2004;18:9-30.

38. Azis N, Reepmeyer C, Clark G, Sriwigati, Tanudirjo DA. Mansiri in North Sulawesi: A new dentate-stamped pottery site in Island Southeast Asia. In: O'Connor S, Bulbeck D, Meyer J, editors. The Archaeology of Sulawesi: Current Research on the Pleistocene to the Historic Period. Acton, Australia: The Australian National University; 2018. p. 191-205.

39. Ono R, Octavianus Sofian H, Aziz N, Sriwigati, Oktaviana AA, Alamsyah N, et al. Traces of Early Austronesian Expansion to East Indonesia? New Discovery of Dentate-Stamped and Lime-Infilled Pottery from Central Sulawesi. The Journal of Island and Coastal Archaeology. 2019;14(1):123-9. doi: 10.1080/15564894.2018.1481897.

40. Brooks ST, Heglar R, Brooks RH. Radiocarbon Dating and Palaeoserology of a Selected Burial Series from the Great Cave of Niah, Sarawak, Malaysia. Asian Perspectives. 1977;20(1):21-31.

41. Anggraeni, Sunarningsih. The Prehistoric Settlement at Jambu Hilir, South Kalimantan Province, Indonesia. IPPA Bulletin. 2008;28:120-6.

42. Azis N, Reepmeyer C, Clark G, Sriwigati, Tanudirjo D. Mansiri in North Sulawesi: A new dentate-stamped pottery site in Island Southeast Asia. In: S OC, D B, J M, editors. The Archaeology of Sulawesi: Current Research on the Pleistocene to the Historic Period. Canberra: Australian National University; 2018. p. 191-205.

43. Galipaud J-C, Kinaston R, Halcrow S, Foster A, Harris N, Simanjuntak T, et al. The Pain Haka burial ground on Flores: Indonesian evidence for a shared Neolithic belief system in Southeast Asia. Antiquity. 2016;90(354):1505-21. Epub 2016/11/21. doi: 10.15184/aqy.2016.185.

44. Petchey FJ, Spriggs M, Bedford S, Valentin F, Buckley H. Radiocarbon dating of burials from the Teouma Lapita cemetery, Efate, Vanuatu. Journal of Archaeological Science. 2014;50(0):227-42. doi: http://dx.doi.org/10.1016/j.jas.2014.07.002.

45. Petchey F, Green R. Use of Three Isotopes to Calibrate Human Bone Radiocarbon Determinations from Kainapirina (SAC), Watom Island, Papua New Guinea. Radiocarbon. 2005;47(2):181-92. Epub 2016/07/18. doi: 10.1017/S0033822200019688.

46. Lape P, Peterson E, Tanudirjo D, Shiung C-C, Lee G-A, Field J, et al. New Data from an Open Neolithic Site in Eastern Indonesia. Asian Perspectives. 2018;57(2):222-43.

47. Bellwood P, Nitihaminoto G, Irwin G, Gunadi, Waluyo A, Tanudirjo D. 35,000 years of prehistory in the northern Moluccas. In: Bartstra G-J, editor. Bird's Head Approaches; Irian Jaya Studies - A Programme for Interdisciplinary Research. Rotterdam: A. A. Balkema; 1988. p. 233-75.

48. Bellwood P. Archaeological Research in Minahasa and the Talaud Islands, Northeastern Indonesia. Asian Perspectives. 1976;19(2):240-88.

49. Summerhayes G, Matisoo-Smith E, Mandui H, Allen J, Specht J, Hogg N, et al. Tamuarawai (EQS): An Early Lapita Site on Emirau, New Ireland, PNG. Journal of Pacific Archaeology. 2010;1(1):62-75.

50. Kirch PV, editor. Lapita and its Transformations in Near Oceania: Archaeological Investigations in the Mussau Islands, Papua New Guinea, 1985-88, Volume I, Introduction, Stratigraphy, Chronology. Berkeley: University of California; 2001.

51. Bellwood P, Dizon E. Archaeological Excavations at Savidug, Sabtang Island. In: Bellwood P, Dizon E, editors. 4000 Years of Migration and Cultural Exchange. terra australis. 40. Canberra: The Australian National University; 2013. p. 47-65.

52. Snow B, Shutler R, Nelson D, Vogel J, Southon J. Evidence of Early Rice Cultivation in the Philippines. Philippine Quarterly of Culture and Society. 1986;14(1):3-11.

53. O’Connor S, Barham A, Spriggs M, Veth P, Aplin K, St Pierre E. Cave Archaeology and Sampling Issues in the Tropics: A Case Study from Lene Hara Cave, a 42,000 Year Old Occupation Site in East Timor, Island Southeast Asia. Australian Archaeology. 2010;71(1):29-40. doi: 10.1080/03122417.2010.11689382.

54. Simanjuntak T. The Western Route Migration: A Second Probable Neolithic Diffusion to Indonesia. In: Piper PJ, Matsumae H, Bulbeck D, editors. New Perspectives in Southeast Asian and Pacific Prehistory. Canberra: Australian National University Press; 2017. p. 201-11.

55. Spriggs M. Archaeology and the Austronesian expansion: where are we now? . Antiquity. 2011;85(328):510-28.

56. Chia S. The Prehistory of Bukit Tengkorak, Sabah, Malaysia. Journal of Southeast Asian Archaeology. 2001;21:146-59.

57. Bellwood P, Waluyo A, Gunadi, Nitihaminoto G, irwin G. Archaeological Research in the Northern Moluccas; Interim Resultss, 1991 Field Season. Bulletin of the Indo-Pacific Prehistory Association. 1993;13:19-33.

58. Tanudirjo DA. Islands in between: prehistory of the northeastern Indonesian archipelago [Thesis]: The Australian National University; 2001.

59. Denham TP, Ramsey CB, Specht J. Dating the appearance of Lapita pottery in the Bismarck Archipelago and its dispersal to Remote Oceania. Archaeology in Oceania. 2012;47(1):39-46. PubMed PMID: 73820639.

60. Kirch PV. A Radiocarbon Chronology for the Mussau Islands. In: Kirch PV, editor. Lapita and its Transformations in Near Oceania: Archaeological Investigations in the Mussau Islands, Papua New Guinea, 1985-88, Volume I, Introduction, Stratigraphy, Chronology. Berkeley: University of California; 2001. p. 196-222.

61. Petchey F, Ulm S. Marine Reservoir Variation in the Bismarck Region: an Evaluation of the Spatial and Temporal Change in ΔR Over the Last 3000 Years. Radiocarbon. 2012;54(1):45-58. doi: DOI 10.2458/azu_js_rc.v54i1.13050. PubMed PMID: WOS:000307006800006.

**Descriptions and Oxcal codes for models excluded from the analysis**

Models were created for multiple site deposits that do not have a sufficient number of radiocarbon determinations (≥4) to warrant inclusion in our analysis. These models are presented here, however, for exploratory purposes and to facilitate future analyses if additional radiocarbon determinations are reported.

**Naidi Site, Batan Island**

The single-phase model contains three radiocarbon determinations obtained from unidentified charcoal. Such a small number of determinations, two of which have large error ranges, results in a date with poor precision.

Plot()

{

Outlier_Model("Charcoal",Exp(1,-10,0),U(0,2.3),"t");

Sequence("Batan Naidi")

{

Boundary("start");

Phase("Naidi")

{

R_Date("ANU-11695",2620,30)

{

Outlier(1);

};

R_Date("ANU-11708",2240,140)

{

Outlier(1);

};

R_Date("ANU-11694",1590,210)

{

Outlier(1);

};

};

Boundary("end");

};

};

**Payaman Site, Batan Island**

The single-phase model contains two radiocarbon determinations obtained from unidentified charcoal. Such a small number of determinations, one of which has a large error range, results in a date with poor precision.

Plot()

{

Outlier_Model("Charcoal",Exp(1,-10,0),U(0,2.3),"t");

Sequence("Batan Payaman")

{

Boundary("start");

Phase("Payaman")

{

R_Date("Wk-13092",1988,47)

{

Outlier(1);

};

R_Date("ANU-12068",1486,185)

{

Outlier(1);

};

};

Boundary("end");

};

};

**Torongan Cave, Itbayat Island**

The single-phase model contains two radiocarbon determinations obtained from charred residue on ceramic sherds. Such a small number of determinations results in a date with poor precision. Red-slipped pottery is associated with these determinations at the base of the cultural layer [6: Table 2.1]

Plot()

{

Outlier_Model("General",T(5),U(0,2.3),"t");

Sequence("Itbayat_Torongan_Cave")

{

Boundary("start");

Phase("Torongan")

{

R_Date("OZH-771",3860,70)

{

Outlier("General",0.05);

};

R_Date("Wk-14642",3320,40)

{

Outlier("General",0.05);

};

};

Boundary("end");

};

};

**Savidug Dune, Sabtang Island**

The Savidug Dune site is the sole dated deposit for Sabtang Island, and thus the site and island models are the same. Bellwood and Dizon’s [51] presentation of the contexts for the radiocarbon determinations contains discrepancies in places (compare text on pages 55 and 57 with Table 5.1 and Figure 4.13). Regardless of the discrepancies in the layer designations and depths for the samples, they appear to relate to the basal ceramic deposit. The determinations are from unidentified charcoal (Wk-19711, Wk-21808, and Wk-21809) and charred residue on a ceramic sherd (Wk-21810). The Savidug determinations are associated with red-slip, circle-stamp, and applique pottery [see e.g., 9: Figure 6.16]

Plot()

{

Outlier_Model("General",T(5),U(0,2.3),"t");

Outlier_Model("Charcoal", Exp(1,-10,0), U(0,2.3), "t");

Sequence("Sabtang_Savidug")

{

Boundary("start");

Phase("Basal ceramic layer")

{

R_Date("Wk-21810",2870,30)

{

Outlier("General",0.05);

};

R_Date("Wk-19711",2828,37)

{

Outlier("Charcoal",1);

};

R_Date("Wk-21809",2416,30)

{

Outlier("Charcoal",1);

};

R_Date("Wk-21808",2146,30)

{

Outlier("Charcoal",1);

};

};

Boundary("end");

};

};

**Andarayan Site, Luzon Island**

The single-phase model contains two radiocarbon determinations obtained from unidentified charcoal (SFU-86) and rice husk and stem (unreported laboratory number). Such a small number of determinations, both of which have large error ranges, results in a date with poor precision. Red-slip pottery is associated with these determinations [52].

Plot()

{

Outlier_Model("General",T(5),U(0,2.3),"t");

Outlier_Model("Charcoal", Exp(1,-10,0), U(0,2.3), "t");

Sequence("Luzon_Andarayan")

{

Boundary("start");

Phase("Andarayan")

{

R_Date("Unreported",3400,125)

{

Outlier("General",0.05);

};

R_Date("SFU-86",3240,160)

{

Outlier("Charcoal",1);

};

};

Boundary("end");

};

};

**Irigayen Site, Luzon Island**

The single-phase model contains four radiocarbon determinations obtained from unidentified charcoal. Such a small number of determinations results in a date with poor precision. This assemblage includes red-slip pottery with a small number of punctate sherds [14].

Plot()

{

Outlier_Model("Charcoal", Exp(1,-10,0), U(0,2.3), "t");

Sequence("Luzon_Irigayen")

{

Boundary("start");

Phase("Irigayen")

{

R_Date("NUTA2-917",3185,25)

{

Outlier(1);

};

R_Date("NUTA2-913",3165,25)

{

Outlier(1);

};

R_Date("NUTA2-914",3025,20)

{

Outlier(1);

};

R_Date("NUTA2-912",2925,20)

{

Outlier(1);

};

};

Boundary("end");

};

};

**Liang Abu Site, Borneo**

The single-phase model contains two radiocarbon determinations obtained from unidentified charcoal. Such a small number of determinations results in a date with poor precision.

Plot()

{

Outlier_Model("Charcoal", Exp(1,-10,0), U(0,2.3), "t");

Sequence("Borneo_Liang Abu")

{

Boundary("start");

Phase("Liang Abu")

{

R_Date("UBA-20839",1672,21)

{

Outlier(1);

};

R_Date("UBA-20840",1524,22)

{

Outlier(1);

};

};

Boundary("end");

};

};

**Bukit Tengkorak Shelter Site, Borneo**

The single-phase model contains two radiocarbon determinations obtained from unidentified charcoal. Such a small number of determinations, both of which have large error estimates, results in a date with poor precision. The radiocarbon determinations from Trench G17 fronting the shelter were not modeled as the basal spit containing ceramics contained no determination. Only one determination was obtained from the penultimate spit containing ceramics.

Plot()

{

Outlier_Model("Charcoal", Exp(1,-10,0), U(0,2.3), "t");

Sequence("Bukit Tengkorak Shelter")

{

Boundary("start");

Phase("Bukit Tengkorak Shelter")

{

R_Date("ANU-5769",2700,110)

{

Outlier(1);

};

R_Date("ANU-5770",2330,170)

{

Outlier(1);

};

};

Boundary("end");

};

};

**Gua Sireh Site, Borneo**

The single-phase model contains two radiocarbon determinations obtained from unidentified charcoal and rice husk embedded in a ceramic sherd. There is a third radiocarbon determination, ANU-7047, but it is unclear if the sample is associated with earliest pottery deposition or might instead provide a TAQ for initial pottery deposition. As such, it was not included in our models. Such a small number of determinations, both of which have large error estimates, results in a date with poor precision.

Plot()

{

Outlier_Model("General",T(5),U(0,2.3),"t");

Outlier_Model("Charcoal", Exp(1,-10,0), U(0,2.3), "t");

Sequence("Gua Sireh")

{

Boundary("start");

Phase("Gua Sireh")

{

R_Date("CAMS-725",3850,260)

{

Outlier("General",0.05);

};

R_Date("ANU-7049",3990,230)

{

Outlier("Charcoal",1);

};

};

Boundary("end");

};

};

**Java Island**

Single radiocarbon determinations from Braholo and Keplek Caves [37] were combined in a single-phase island-wide model for Java. Both determinations are from unidentified charcoal and both have large error estimates. These determinations are associated with pottery described as earthenware [37].

Plot()

{

Outlier_Model("Charcoal", Exp(1,-10,0), U(0,2.3), "t");

Sequence("Java")

{

Boundary("start");

Phase("Java")

{

R_Date("P3G-1998",3260,110)

{

Outlier(1);

};

R_Date("P3G-1999",3050,100)

{

Outlier(1);

};

};

Boundary("end");

};

};

**Kamassi, Sulawesi Island**

A chronology of Kamassi site deposits on Sulawesi must rely on four radiocarbon determinations, all from freshwater (*Melanoides* sp.) and estuarine shells (*Geloina* sp.). Thus the Kamassi site results were not used in our analysis, but the radiocarbon data is included in S3 Table.

**Malawa Site, Sulawesi Island**

The single-phase model contains two radiocarbon determinations obtained from unidentified charcoal. Such a small number of determinations, both of which have large error values, results in a date with poor precision.

Plot()

{

Outlier_Model("Charcoal", Exp(1,-10,0), U(0,2.3), "t");

Sequence("Sulawesi Malawa")

{

Boundary("start");

Phase("Malawa")

{

R_Date("P3G-06",3580,130)

{

Outlier(1);

};

R_Date("P3G-06",2710,170)

{

Outlier(1);

};

};

Boundary("end");

};

};

**Lena Hara Site, Timor**

Two determinations from Square F, spit 16 (61-68 cm bs) were included in a single-phase model for Lena Hara Cave, Timor. One determination is from unidentified charcoal adhering to the exterior surface of a sherd and the other determination is from a marine gastropod (*Trochus niloticus*). The radiocarbon determinations are associated with red-slipped and plain pottery [53].

Plot()

{

Outlier_Model("General",T(5),U(0,2.3),"t");

Outlier_Model("Charcoal", Exp(1,-10,0), U(0,2.3), "t");

Sequence("Lena Hara Cave")

{

Boundary("start");

Phase("Square F, Spit 16")

{

R_Date("ANU-12029",3200,240)

{

Outlier("Charcoal",1);

};

Curve("Marine20","Marine20.14c");

Delta_R("LocalMarine", 0,0); [this should be revised based on a localized correction value]

R_Date("ANU-12041,3850,70)

{

Outlier("General",0.05);

};

};

Boundary("end");

};

};

**Um Kapat Papo, Gebe Island**

The single-phase model contains two radiocarbon determinations obtained from marine shell. Such a small number of determinations results in a date with poor precision. The lack of a localized ΔR value is also a confounding factor.

Plot()

{

Outlier_Model("General",T(5),U(0,2.3),"t");

Sequence("Um Kapat Papo")

{

Boundary("start");

Phase("Layer 3")

{

Curve("Marine20","Marine20.14c");

Delta_R("LocalMarine", 0,0);

R_Date("ANU-9317",4830,70)

{

Outlier("General",0.05);

};

R_Date("ANU-9316",2030,60)

{

Outlier("General",0.05);

};

};

Boundary("end");

};

};

**Uattamdi, Kayoa Island**

The two-phase model contains two radiocarbon determinations obtained from marine shell. Such a small number of determinations results in a date with poor precision and phases with single determinations are inappropriate. The lack of a localized ΔR value is also a confounding factor.

Plot()

{

Outlier_Model("General",T(5),U(0,2.3),"t");

Sequence("Uattamdi")

{

Boundary("start Layer E");

Phase("Layer E")

{

Curve("Marine20","Marine20.14c");

Delta_R("LocalMarine",0,0);

R_Date("ANU-3530", 3530,70)

{

Outlier("General",0.05);

};

};

Boundary("transition Layer E/Layer D");

Phase("Layer D")

{

Curve("Marine20","Marine20.14c");

Delta_R("LocalMarine",0,0);

R_Date("ANU-7776",3440,110)

{

Outlier("General",0.05);

};

R_Date("ANU-9323",3260,70)

{

Outlier("General",0.05);

};

};

Boundary("end");

};

};

**ECB Site, Eloaua Island**

The single-phase model for the ECB site on Eloaua Island contains one radiocarbon determination obtained from unidentified charcoal and two radiocarbon determinations from marine shell. Such a small number of determinations results in a date with poor precision.

Plot()

{

Outlier_Model("General",T(5),U(0,2.3),"t");

Outlier_Model("Charcoal",Exp(1,-10,0),U(0,2.3),"t");

Sequence("ECB")

{

Boundary("start");

Phase("ECB")

{

R_Date("Beta-20453",3200,70)

{

Outlier(1);

};

Curve("Marine20","Marine20.14c");

Delta_R("LocalMarine", -293,92);

R_Date("ANU-5086",3120,80)

{

Outlier("General",0.05);

};

R_Date("ANU-5087",3150,80)

{

Outlier("General",0.05);

};

};

Boundary("end");

};

};

**EQS Site, Emirau Island**

The single-phase model contains two radiocarbon determinations obtained from unidentified charcoal. Such a small number of determinations results in a date with poor precision.

Plot()

{

Outlier_Model("Charcoal", Exp(1,-10,0), U(0,2.3), "t");

Sequence("EQS")

{

Boundary("start");

Phase("EQS")

{

R_Date("Wk-21349",3044,31)

{

Outlier(1);

};

R_Date("Wk-21345",2917,31)

{

Outlier(1);

};

};

Boundary("end");

};

};

**Supporting Information Figures and Tables**


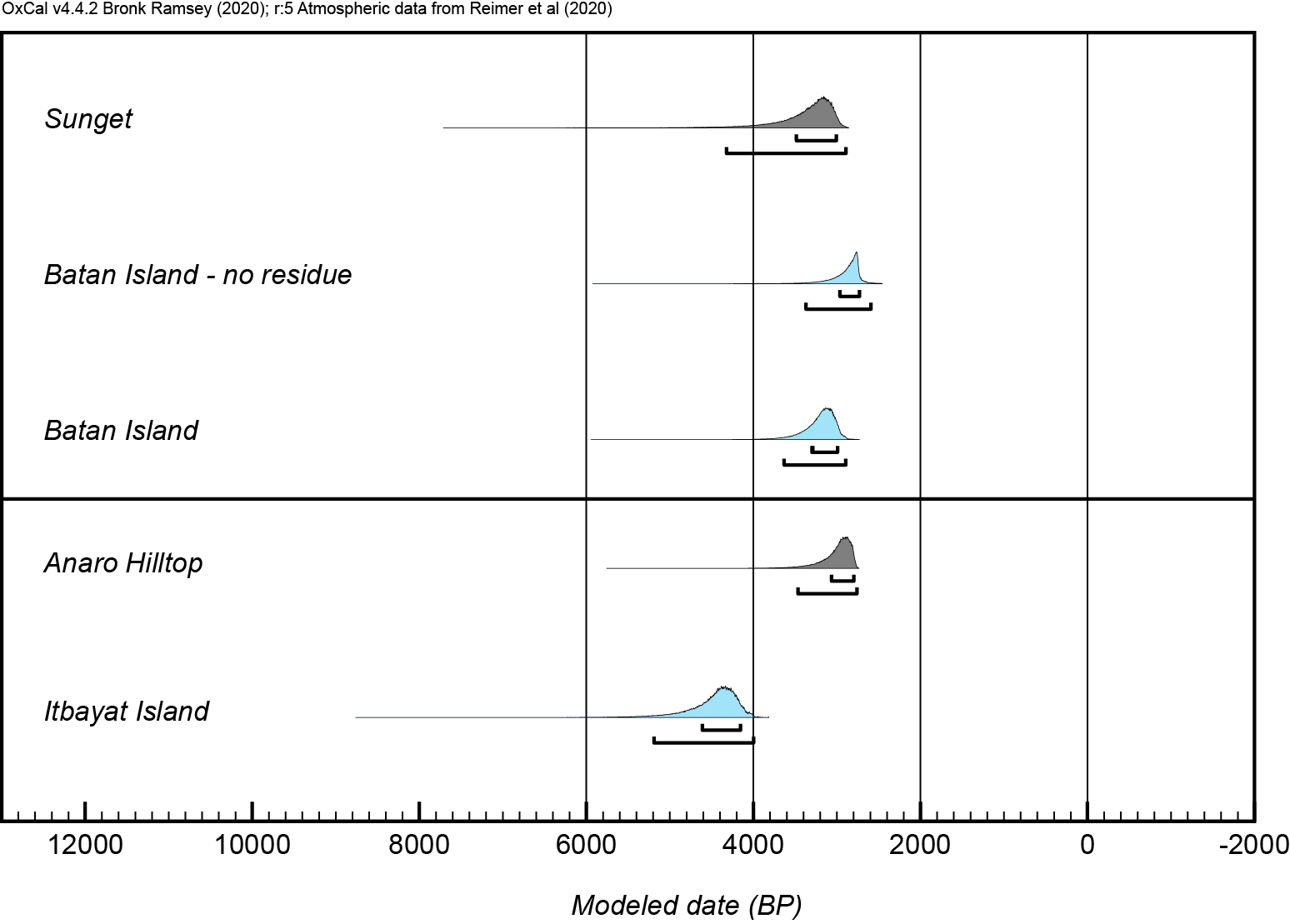


S1 Fig. Modeled Date Range Distributions for the Appearance of Pottery in the Batanes Islands. Island-scale date ranges are light blue and site-scale date ranges are gray. Lines beneath each distribution delimit 95.4% and 68.2% HPD.


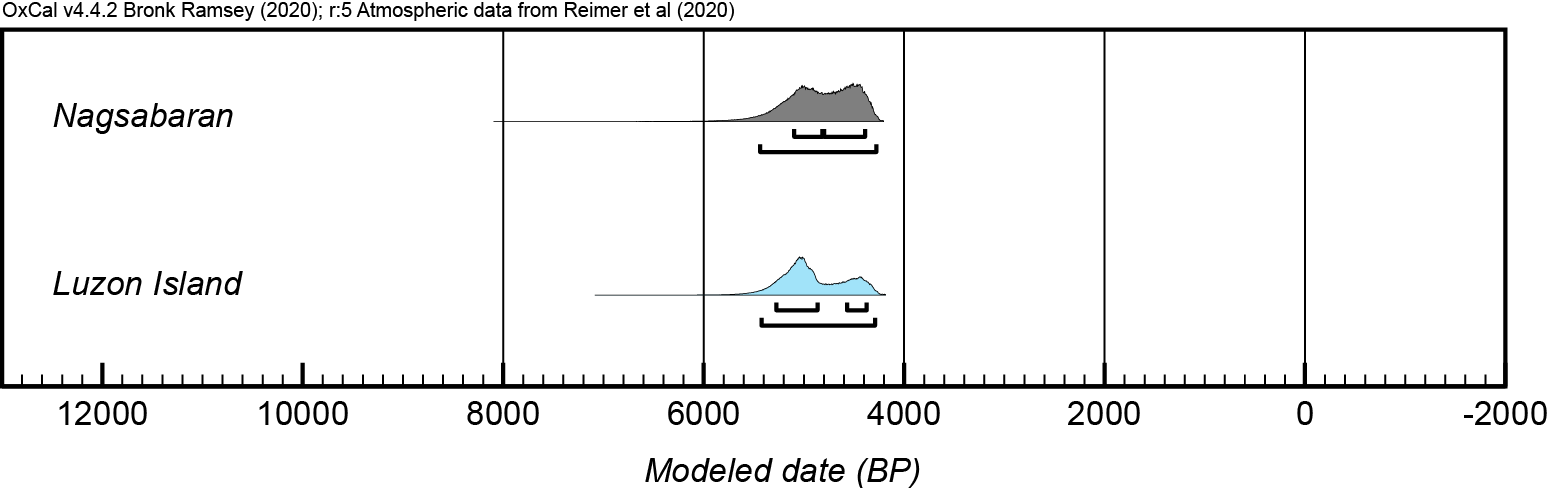


**S2 Fig.** **Modeled Date Range Distributions for the Appearance of Pottery on Luzon Island, northern Philippines.** Island-scale date range is light blue and site-scale date range is gray. The Nagsabaran HPD is from our preferred model (two phase, excluding the oldest silt layer determination and the five oldest shell midden determinations). Lines beneath each distribution delimit 95.4% and 68.2% HPD.


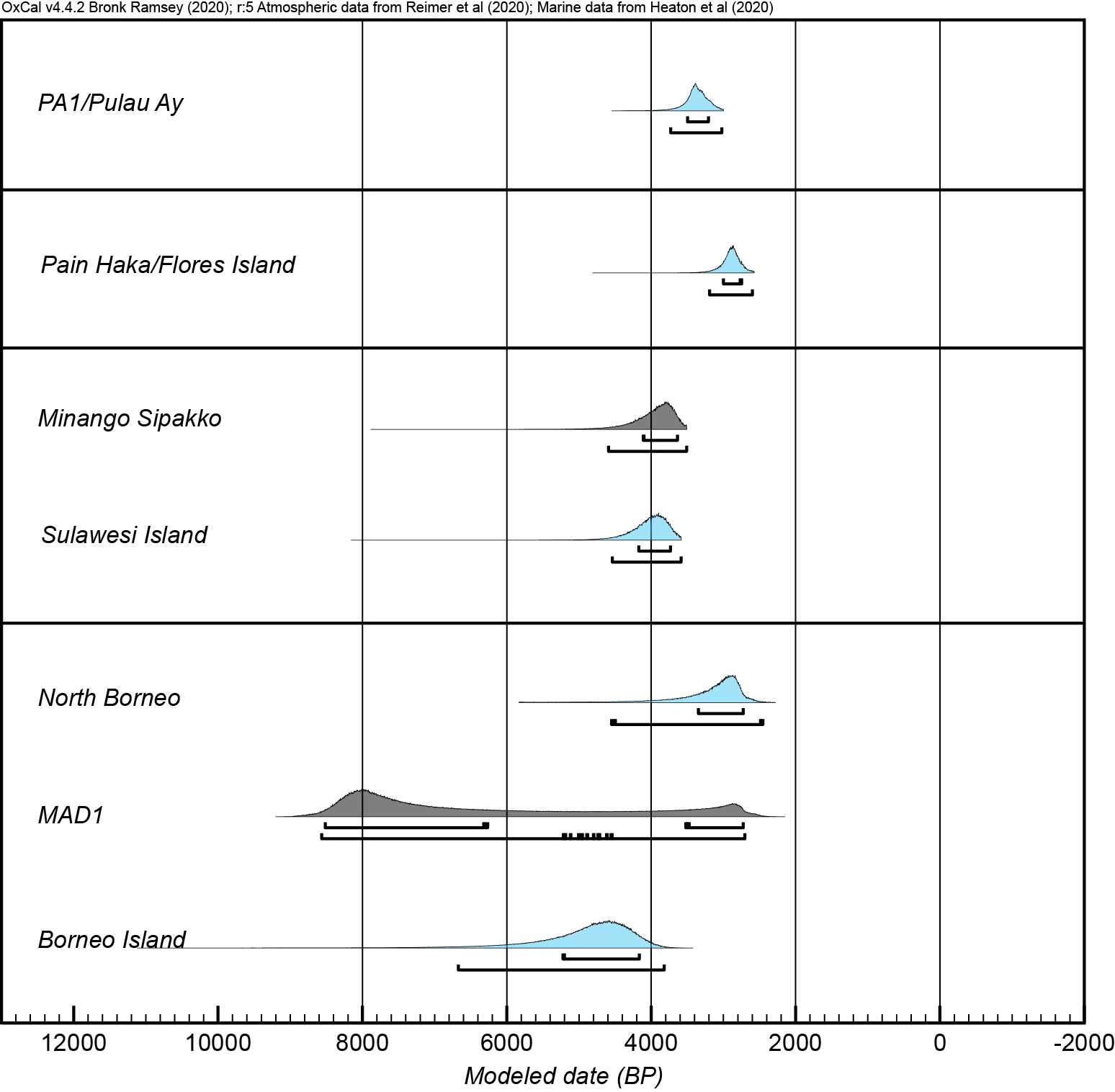


**S3 Fig.** **Modeled Date Range Distributions for the Appearance of Pottery on Borneo and Sulawesi (Greater Sunda Islands), and Flores (Lesser Sunda Islands), and Pulau Ay (Molucca Islands).** Island-scale date range is light blue and site-scale date range is gray. Lines beneath each distribution delimit 95.4% and 68.2% HPD.


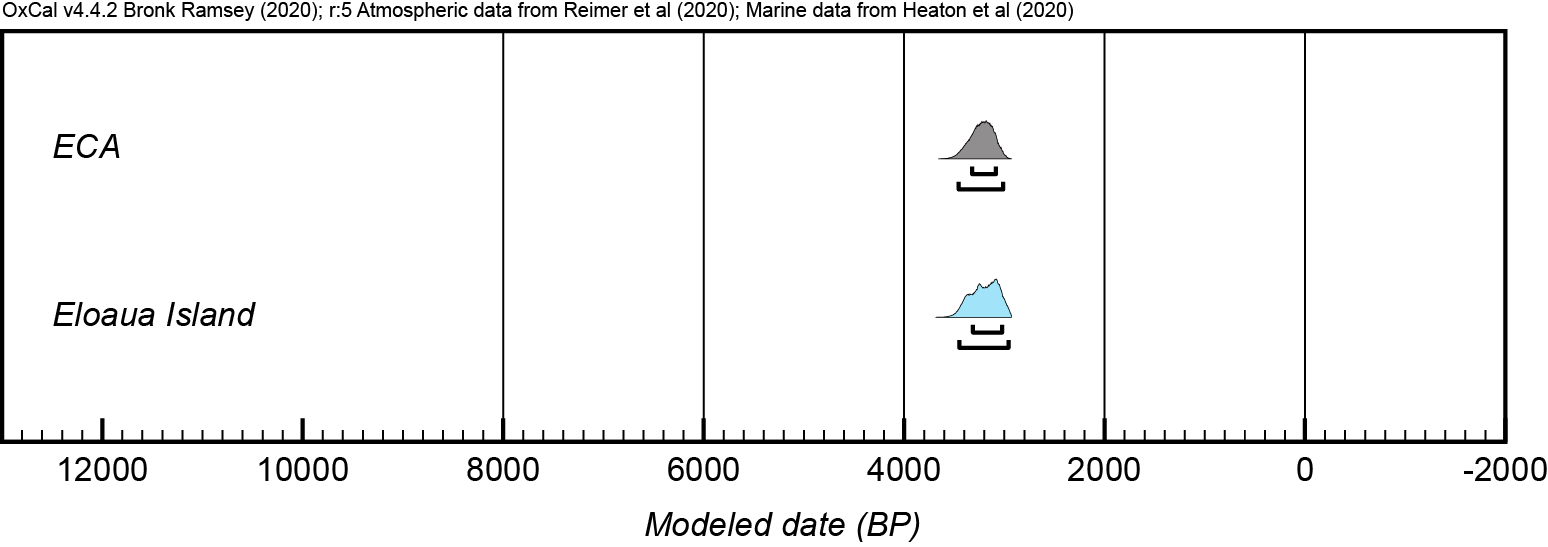


**S4 Fig.** Modeled Date Range Distributions for the Appearance of Pottery in the Mussau islands. Lines beneath each distribution delimit 95.4% and 68.2% HPD.

S1 Table. Order analysis of the island-scale models for the initial deposition of pottery. Probability that t1 is older than t2; >0.5 then older, <0.5 then younger

| **Probability *t*_1_ < *t*_2_** |  |  |  |  |  |  |  |  |  |  |
| --- | --- | --- | --- | --- | --- | --- | --- | --- | --- | --- |
| ***t*_1_** | ***t*_2_** | | | | | | | | | |
|  | Luzon | Borneo | Itbayat | Sulawesi | Pulau Ay | Eloaua | Batan | N. Borneo | Flores | Batan (no residue) |
| Luzon | 0 | 0.5554 | 0.8355 | 0.9761 | 0.9998 | 1 | 0.9996 | 0.9715 | 1 | 0.9996 |
| Borneo | 0.4446 | 0 | 0.732 | 0.9374 | 0.9987 | 1 | 0.9991 | 0.9671 | 1 | 0.9992 |
| Itbayat | 0.16448 | 0.26796 | 0 | 0.896 | 0.998 | 1 | 0.9986 | 0.9515 | 0.9999 | 0.9988 |
| Sulawesi | 0.023937 | 0.06258 | 0.10402 | 0 | 0.9831 | 1 | 0.9905 | 0.9121 | 0.9994 | 0.9957 |
| Pulau Ay | 0.000241 | 0.001297 | 0.002033 | 0.016861 | 0 | 0.762 | 0.788 | 0.743 | 0.9807 | 0.9582 |
| Eloaua | 0 | 0.000002 | 0 | 0.000015 | 0.23805 | 0 | 0.5935 | 0.6551 | 0.9557 | 0.9256 |
| Batan | 0.000365 | 0.000916 | 0.001406 | 0.00952 | 0.21201 | 0.4065 | 0 | 0.6156 | 0.9244 | 0.9004 |
| N. Borneo | 0.028527 | 0.03287 | 0.04848 | 0.08788 | 0.25697 | 0.3449 | 0.3844 | 0 | 0.72 | 0.745 |
| Flores | 0.000005 | 0.000032 | 0.000054 | 0.00062 | 0.019303 | 0.04431 | 0.07555 | 0.27997 | 0 | 0.5758 |
| Batan (no residue) | 0.000443 | 0.000759 | 0.001172 | 0.004259 | 0.04176 | 0.0744 | 0.09957 | 0.255 | 0.4242 | 0 |

S2 Table. Order analysis of the site-scale models for the initial deposition of pottery. Probability that t1 is older than t2; >0.5 then older, <0.5 then younger.

| **Probability *t*_1_ < *t*_2_** | | | | | | | | |
| --- | --- | --- | --- | --- | --- | --- | --- | --- |
| ***t*_1_** | ***t*_2_** | | | | | | | |
|  | **MAD1** | **Nagsabaran** | **Minango Sipakko** | **PA1** | **Sunget** | **ECA** | **Anaro Hilltop** | **Pain Haka** |
| **MAD1** | 0 | 0.7259 | 0.8061 | 0.8727 | 0.8726 | 0.8946 | 0.9304 | 0.9496 |
| **Nagsabaran** | 0.27415 | 0 | 0.9644 | 0.9997 | 0.9763 | 1 | 0.999 | 1 |
| **Minango Sipakko** | 0.1939 | 0.03564 | 0 | 0.9748 | 0.8975 | 0.9999 | 0.9896 | 0.999 |
| **PA1** | 0.12733 | 0.000318 | 0.025235 | 0 | 0.5859 | 0.7912 | 0.9179 | 0.9805 |
| **Sunget** | 0.12735 | 0.023694 | 0.10247 | 0.4141 | 0 | 0.618 | 0.8714 | 0.9563 |
| **ECA** | 0.10543 | 0 | 0.000128 | 0.20881 | 0.382 | 0 | 0.8495 | 0.9544 |
| **Anaro Hilltop** | 0.06956 | 0.001024 | 0.010357 | 0.08205 | 0.12859 | 0.15055 | 0 | 0.6706 |
| **Pain Haka** | 0.05041 | 0.000011 | 0.001014 | 0.019497 | 0.04365 | 0.04558 | 0.3294 | 0 |

**S3 Table.** **Radiocarbon sample data**.

| **Island Group** | **Island/region** | **Provenience** | **Laboratory number** | **Sample details*** | **Radiocarbon age (BP)** | **Error** | **δ^13^C (‰)** | **Dating Method**† | **Notes** | **Ceramic**  **Surface Modifications** | **Reference** |
| --- | --- | --- | --- | --- | --- | --- | --- | --- | --- | --- | --- |
| Batanes | Batan | Mahatao Town Septic Tank, Auger M3, cultural palaeosol at 250 cm bs below volcanic ash (with sherds) | ANU-11710 | UC | 2090 | 60 | - | A | Sample recovered from augered deposit with sherds and charcoal within an identifiable stratum. | red slip | [3:45, 4:149] |
| Batanes | Batan | Naidi (Basco-Songsong Road), 0-10 cm, charcoal in north road section | ANU-11695 | UC | 2620 | 30 | -25 | C | "Excluding the presumably out of context ANU 11709, the remaining dates give a calibrated age range of between 835 BC to AD 900 - in actuality a much wider range that can be accommodated by such a shallow archaeological deposit. The main concentration of dates points to the late first millennium BC for the Naidi assemblage" [4:149]. "The closest parallels to these Naidi Phase assemblages discussed so far lie in the lower cultural layer at Savidug Dune Site and at Anaro, but the absence of stamped circles clearly places the Naidi pottery later than the Phase 2 material from these two sites." [2:111]. | red slip | [2:71, Table 5.1, 4] |
| Batanes | Batan | Naidi (Basco-Songsong Road), 0-10 cm, charcoal in south road section | ANU-11694 | UC | 1590 | 210 | -24.9 | C | See ANU-11695. | red slip | [2:71, Table 5.1, 4] |
| Batanes | Batan | Naidi (Basco-Songsong Road), Pit A1, 0-10 cm (within layer) | ANU-11709 | UC | 200 | 360 | - | C | This date is anomalous. The paleosol is capped by volcanic ash that was deposited between 1500-1000 BP. | red slip | [4:145, Table 1] |
| Batanes | Batan | Naidi (Basco-Songsong Road), Pit A2, 0-10 cm | ANU-11708 | UC | 2240 | 140 | -26.7 | C | See ANU-11695. | red slip | [2:71, Table 5.1, 4] |
| Batanes | Batan | Payaman, north square, Layer III, 10-25 cm within layer | Wk-13092 | UC | 1988 | 47 | -26.9 | C |  | red slip | [2:71, Table 5.1, 4:150] |
| Batanes | Batan | Payaman, south square, Layer III, 20-25 cm within layer | ANU-12068 | UC | 1486 | 185 | - | C | Based on profile photograph, sample-depth should be very near the base of the unit. | red slip | [2:71, Table 5.1, 4:150] |
| Batanes | Batan | Sunget Main Terrace, Layer V, 15-20 cm (within layer) | OZH-776 | Resin coating on sherd exterior | 5790 | 150 | - | A | Author suggests date might not date sherds and be from "fossil resin" on sherd. | red slip, circle-stamped | [2:70, Table 5.1, 4:150] |
| Batanes | Batan | Sunget Main Terrace, Layer V, 15-20 cm (within layer) | Wk-14640 | Residue on sherd | 2915 | 49 | -26 | A |  | red slip, circle-stamped | [1:14, 2:71, Table 5.1] |
| Batanes | Batan | Sunget Main Terrace, Layer V, 20-30 cm (within layer) | ANU-11817 | Residue on sherd | 2910 | 190 | - | A |  | red slip, circle-stamped | [2:71, Table 5.1,1:14] |
| Batanes | Batan | Sunget Main Terrace, Layer V, 30-35 cm (within layer, scattered charcoal fragments) | Wk-15649 | UC | 2383 | 35 | -25.9 | A | Author suggests charcoal dates from more recent use of site, possibly from digging sticks and charcoal. | red slip, circle-stamped | [2:71, Table 5.1,1:14] |
| Batanes | Batan | Sunget Top Terrace, Layer V, 20-30 cm, charcoal concentration (possible hearth) | ANU-11693 | UC | 2630 | 30 | -27.0 | C | Author suggests may date from later activity at site. | red slip, circle-stamped | [2:71, Table 5.1,1:14] |
| Batanes | Batan | Sunget Top Terrace, Squares A/D, Layer V, 20-30 cm (within layer) | ANU-11707 | UC | 2000 | 140 | -27.6 | A | Author suggests charcoal dates from more recent use of site, possibly from digging sticks and charcoal. | red slip, circle-stamped | [2:71, Table 5.1, 5:131] |
| Batanes | Batan | Tayid, beneath main ash deposit | ANU-12069 | Residue on sherd | 1842 | 215 | - | A |  | red slip | [2:70, Table 5.1] |
| Batanes | Batan | North of Basco, B10 outcrop | Gif-6575 | UC | 2310 | 80 | - |  | Cannot be clearly used for determining ceramic chronology; included in dataset for information. | unstated | [8:13] |
| Batanes | Itbayat | Anaro Hilltop Site, Area 2A, 15-20 cm | Wk-14643 | Residue on sherd | 1876 | 41 | -26 | A | Bellwood & Dizon [2: Table 5.3] indicate Anaro ceramic period is 1200 BC - 600 AD | circle-stamped | [2:70, Table 5.1] |
| Batanes | Itbayat | Anaro Hilltop Site, Area 3, 90-95 cm | Wk-14645 | Residue on sherd | 1360 | 39 | -23.6 | A | See Wk-14643. | circle-stamped | [2:70, Table 5.1] |
| Batanes | Itbayat | Anaro Hilltop Site, Area 3, 95-105 cm | OZH-774 | Residue on sherd | 2770 | 50 | -23.6 | A | See Wk-14643. | circle-stamped | [2:70, Table 5.1] |
| Batanes | Itbayat | Anaro Hilltop Site, Area 3A (inside rockshelter), 100-110 cm | OZJ-693 | Residue on sherd | 2475 | 45 | -24 | C | See Wk-14643. | circle-stamped | [2:70, Table 5.1] |
| Batanes | Itbayat | Anaro Hilltop Site, Area 3A (inside rockshelter), 80-90 cm | OZJ-692 | Residue on sherd | 2095 | 45 | -25.2 | C | See Wk-14643. | circle-stamped | [2:70, Table 5.1] |
| Batanes | Itbayat | Anaro Hilltop Site, Area 3B, 70-75 cm | OZJ-695 | Residue on sherd | 2080 | 45 | -26.3 | C | See Wk-14643. | circle-stamped | [2:70, Table 5.1] |
| Batanes | Itbayat | Anaro Hilltop Site, Area 3B, 85-90 cm | OZJ-696 | Residue on sherd | 1375 | 45 | -25.9 | C | Bellwood et al. [9:83] argue that these young dates are the result of the movement of younger material traveling downwards into the profile. | circle-stamped | [2:70, Table 5.1] |
| Batanes | Itbayat | Anaro Hilltop Site, Area 3B, 90-95 cm | OZJ-697 | Residue on sherd | 1510 | 60 | -24.4 | C | See OZJ-696. | circle-stamped | [2:70, Table 5.1] |
| Batanes | Itbayat | Anaro Hilltop Site, Area 3B, 65-70 cm | OZJ-694 | Residue on sherd | 1280 | 45 | -20.7 | C | See Wk-14643. | circle-stamped | [2:70, Table 5.1] |
| Batanes | Itbayat | Torongan Cave, Squares A-D, 55-60 cm (base of cultural layer) | OZH-771 | Residue on sherd | 3860 | 70 | - |  | Shell samples from same deposit not included in Cochrane et al. analysis as could be washed into cave from above [6:12-14]. | red-slip | [3:chapters 3, 5] |
| Batanes | Itbayat | Torongan Cave, Squares A-D, 55-60 cm (base of cultural layer) | Wk-14642 | Residue on sherd | 3320 | 40 | -25.3 |  | Shell samples from same deposit not included in Cochrane et al. analysis as could be washed into cave from above [6:12-14]. | red-slip | [3:chapters 3, 5] |
| Batanes | Sabtang | Savidug Dune Site, Trench R7-9, Layer 5, 210 cm bs | Wk-21808 | UC | 2146 | 30 | -26.9 |  |  | red-slip | [3:chapters 3, 5] |
| Batanes | Sabtang | Savidug Dune Site, Trench R7-9, Layer 4, 135 cm bs, near jade ornament | Wk-21809 | UC | 2416 | 30 | -24.6 |  |  | red-slip | [3:chapters 3, 5] |
| Batanes | Sabtang | Savidug Dune Site, Trench R7-9, Layer 5, 220 cm bs | Wk-21810 | Residue on sherd | 2870 | 30 | -25.8 |  |  | red-slip | [3:chapters 3, 5] |
| Batanes | Sabtang | Savidug Dune Site, unit 5, Layer 5, 180 cm bs | Wk-19711 | UC | 2828 | 37 | -26.7 |  | Exact association between charcoal and sherds unclear. Bellwood & Dizon [9:100] state sherds “were stratified just above the radiocarbon date of c.1000 BC (Wk-19711, Table 5.1)”. Date comes from Layer 5, but sherds, apparently, in Layer 4. | red-slip, circle-stamped | [3:chapters 3, 5] |
| Northern Philippines | Luzon | Nagsabaran, “cultural layer beneath shell midden” | Beta-437271 | “Rice grain charcoal” | 2550 | 30 | - |  | No detailed contextual or provenience information given. No information on methods of identification for rice-grain charcoal. Deposit with dated material is “just prior” to the shell midden layer. | unstated | [12:799, Table 1] |
| Northern Philippines | Luzon | Nagsabaran, test pit dug in 2016, “Near the base of site stratigraphy” | Beta-436818 | UC | 3760 | 30 | - |  | No detailed contextual or provenience information given. Not clear if deposit associated with determination is stratigraphically inferior or contemporaneous with the lower silt layer and red-slipped pottery. | unstated | [12:799, Table 1] |
| Northern Philippines | Luzon | Nagsabaran, TP 1, 80 cm bs, shell midden | GX-26797 | UC | 1470 | 50 | - |  | The shell midden layer is stratigraphically superior to the silty clay alluvial deposits containing red-slipped pottery. | unstated | [11:Table 2] |
| Northern Philippines | Luzon | Nagsabaran, TP 1, 110 cm bs, shell midden | GX-26798 | UC | 1670 | 60 | - |  | The shell midden layer is stratigraphically superior to the silty clay alluvial deposits containing red-slipped pottery. | unstated | [11:Table 2] |
| Northern Philippines | Luzon | Nagsabaran, TP 1, 120 cm bs, shell midden | GX-26705 | UC | 2120 | 220 | - |  | The shell midden layer is stratigraphically superior to the silty clay alluvial deposits containing red-slipped pottery; note: two separate samples were submitted and assigned the GX-26705 laboratory number. | unstated | [11:Table 2] |
| Northern Philippines | Luzon | Nagsabaran, TP 1, 140 cm bs, shell midden | GX-26698 | UC | 1830 | 70 | - |  | The shell midden layer is stratigraphically superior to the silty clay alluvial deposits containing red-slipped pottery. | unstated | [11: Table 2] |
| Northern Philippines | Luzon | Nagsabaran, TP 1, 150 cm bs, shell midden | GX-26799 | UC | 1960 | 90 | - |  | The shell midden layer is stratigraphically superior to the silty clay alluvial deposits containing red-slipped pottery. | unstated | [11: Table 2] |
| Northern Philippines | Luzon | Nagsabaran, TP 1, 180 cmbs, shell midden | GX-26699 | UC | 1920 | 80 | - |  | The shell midden layer is stratigraphically superior to the silty clay alluvial deposits containing red-slipped pottery | unstated | [11: Table 2] |
| Northern Philippines | Luzon | Nagsabaran, TP, 1, 180 cmbs, shell midden | GX-26800 | UC | 1760 | 110 | - |  | The shell midden layer is stratigraphically superior to the silty clay alluvial deposits containing red-slipped pottery | unstated | [11: Table 2] |
| Northern Philippines | Luzon | Nagsabaran, TP 1, 230 cmbs, shell midden | GX-26801 | UC | 2260 | 270 | - |  | The shell midden layer is stratigraphically superior to the silty clay alluvial deposits containing red-slipped pottery | unstated | [11: Table 2] |
| Northern Philippines | Luzon | Nagsabaran, TP 1, 240 cmbs, shell midden | GX-26802 | UC | 2240 | 270 | - |  | The shell midden layer is stratigraphically superior to the silty clay alluvial deposits containing red-slipped pottery | unstated | [11: Table 2] |
| Northern Philippines | Luzon | Nagsabaran, TP 1, 250 cmbs, shel midden | GX-26702-AMS | UC | 1820 | 40 | - | A | The shell midden layer is stratigraphically superior to the silty clay alluvial deposits containing red-slipped pottery | unstated | [11: Table 2] |
| Northern Philippines | Luzon | Nagsabaran, TP 1, 310 cmbs, lower silt layer | NTU-3799 | *Batissa childreni* | 3450 | 40 | - | A | The silty clay alluvial deposits containing red-slipped pottery | Red-slip | [11: Table 2] |
| Northern Philippines | Luzon | Nagsabaran, TP 2, 140 cmbs, lower silty layer | GX-26704-AMS | UC | 2620 | 40 | - | A | The silty clay alluvial deposits containing red-slipped pottery. Hung et al. [11] argue that determinations is too recent and thus must be out of context. | red-slip | [11: Table 2] |
| Northern Philippines | Luzon | Nagsabaran, TP 2, 150 cmbs, lower silty layer | GX-26705 | UC | 6610 | 290 | - |  | The silty clay alluvial deposits containing red-slipped pottery. Hung et al. [11] argue that determinations is too old and thus must be out of context; note: two separate samples were submitted and assigned the GX-26705 laboratory number. | red-slip | [11: Table 2] |
| Northern Philippines | Luzon | Nagsabaran, TP 4, 150 cmbs, shell midden | GX-26806 | UC | 2150 | 150 | - |  | The shell midden layer is stratigraphically superior to the silty clay alluvial deposits containing red-slipped pottery; | unstated | [11: Table 2] |
| Northern Philippines | Luzon | Nagsabaran, TP 4, 210 cmbs, lower silty layer | GX-26711-AMS | UC | 2520 | 50 | - | A | Hung et al. [11, 17] argue that determination is too recent and thus must be out of context. | red-slip | [11: Table 2] |
| Northern Philippines | Luzon | Nagsabaran, TP7, 160 cm | NTU-3798 | UC | 2670 | 40 | - |  | Hung et al. [11] argue that determination is too recent and thus must be out of context. | red-slip, incised, impressed, dentate | [10:Table 1, 11:Table 2] |
| Northern Philippines | Luzon | Nagsabaran, TP7, 160 cm | GX-28379 | UC | 3050 | 70 | - |  | See NTU-3798. | red-slip, incised, impressed, dentate | [10:Table 1, 11:Table 2] |
| Northern Philippines | Luzon | Nagsabaran, TP7, 190 cm | GX-28381 | UC | 3390 | 130 | - |  | See NTU-3798. | red-slip, incised, impressed, dentate | [10:Table 1, 11:Table 2] |
| Northern Philippines | Luzon | Nagsabaran, TP9, 150 cm | Wk-19712 | Animal bone | 2504 | 35 | - | C | Transition between basal and middle silt layer; WK-19712 is stratigraphically superior to WK-18059. Note that some publications do not distinguish different silt layers. Date from gelatin, though yield was low (<0.5%), likely *Sus species* based on stable isotope results (F. Petchey, personal communication, Nov. 8, 2020). | red-slip, incised, impressed, dentate | [5:159, Table 7.1] |
| Northern Philippines | Luzon | Nagsabaran, TP9, 150 cm | Wk-19713 | UC | 4450 | 39 | - | C | Transition between basal and middle silt layer; WK-19713 and 19712 should agree (same context); WK-19713 is stratigraphically superior to WK-17756 and WK-18059. Hung et al. [11] suggest the determination is too old and must be out of context. Note that some publications do not distinguish different silt layers. | red slip, incised, impressed, dentate | [5:159, Table 7.1] |
| Northern Philippines | Luzon | Nagsabaran, TP9, 160 cm | Wk-18059 | UC | 1946 | 30 | - | C | Upper margin of basal silt layer (based on profile). Hung et al. [11] argue that determinations is too recent and thus must be out of context. | red slip, incised, impressed, dentate | [5:159, Table 7.1] |
| Northern Philippines | Luzon | Nagsabaran, TP9, 180 cm | Wk-17756 | UC | 2528 | 31 | - | C | Lower margin of basal silt layer (based on profile). Hung et al. [11] argue that determinations is too recent and thus must be out of context. | red slip, incised, impressed | [5:159, Table 7.1] |
| Northern Philippines | Luzon | Nagsabaran, TP9, Level 14, 130-140 cm | Wk-23997 [mistakenly reported as Wk-23397] | *Sus scrofa/ verrucosus* lower fourth premolar (Cat. 2004-12-16) | 3940 | 40 | - | C | Piper et al. [19:78] state tooth originates from TP 9, level 14 (130-140 bmg [below modern ground level]; this sample is stratigraphically superior to all other samples in TP 9 yet has the oldest date in the sequence. Date from gelatin, though the yield was low (0.44%), δ^15^N = 5.72, δ^13^C = -22.97 (F. Petchey, personal communication, Nov. 8, 2020). | red slip, incised, impressed, circle-stamped, dentate | [19:78, 20:691] |
| Northern Philippines | Luzon | Nagsabaran, TP11, 170 cm | ANU-13016 | UC | 3510 | 30 | -26.5 |  | See NTU-3798. | red slip, incised, impressed | 10:Table 1, 11:Table 2] |
| Northern Philippines | Luzon | Nagsabaran, TP14, 80 cm bs, shell midden | ANU-13020 | *Batissa childreni* | 2620 | 30 | -12.5 |  | The shell midden layer is stratigraphically superior to the silty clay alluvial deposits containing red-slipped pottery. | Unstated | [11: Table 2] |
| Northern Philippines | Luzon | Nagsabaran, TP14, 120 cm bs, shell midden | ANU-13019 | *Batissa childreni* | 2560 | 30 | -8.9 |  | The shell midden layer is stratigraphically superior to the silty clay alluvial deposits containing red-slipped pottery. | unstated | [11: Table 2] |
| Northern Philippines | Luzon | Nagsabaran, TP14, 140 cm bs, shell midden | ANU-13018 | *Batissa childreni* | 7380 | 40 | -26.4 |  | The shell midden layer is stratigraphically superior to the silty clay alluvial deposits containing red-slipped pottery. | unstated | [11: Table 2] |
| Northern Philippines | Luzon | Nagsabaran, TP14, 180 cm bs, shell midden | ANU-13017 | *Batissa childreni* | 3420 | 30 | -10.4 |  | The shell midden layer is stratigraphically superior to the silty clay alluvial deposits containing red-slipped pottery. | unstated | [11: Table 2] |
| Northern Philippines | Luzon | Nagsabaran, TP14, 210 cm bs, shell midden | ANU-13024 | *Batissa childreni* | 2680 | 30 | -12.6 |  | The shell midden layer is stratigraphically superior to the silty clay alluvial deposits containing red-slipped pottery. | unstated | [11: Table 2] |
| Northern Philippines | Luzon | Nagsabaran, TP14, 240 cm | ANU-13014 | UC | 2660 | 30 | -27.4 |  | See NTU-3798. Hung et al. [11] argue that determinations is too recent and thus must be out of context. | red slip, incised, impressed, circle-stamped, dentate | [10:Table 1, 11 |
| Northern Philippines | Luzon | Nagsabaran, TP14, 240 cm | ANU-13013 | UC | 2540 | 30 | -31.6 |  | See NTU-3798. Hung et al. [11] argue that determinations is too recent and thus must be out of context. | red slip, incised, impressed, circle-stamped, dentate | [10:Table 1, 11 |
| Northern Philippines | Luzon | Andarayan Site | SFU-86 | UC | 3240 | 160 | - | C |  | red slip | [13:3, 52] |
| Northern Philippines | Luzon | Nagsabaran, Cagayan River | ANU-13021 | *Batissa children* | 98.47% modern |  | -12.7 |  |  | unstated | [11: Table 2] |
| Northern Philippines | Luzon | Nagsabaran, Zabaran Creek | ANU-13023 | *Batissa children* | 103.14% modern |  | -15.1 |  |  | unstated | [11: Table 2] |
| Northern Philippines | Luzon | Nagsabaran, Zabaran Creek | ANU-15410 | *Batissa children* | 104.83% modern |  | -13 |  |  | unstated | [11: Table 2] |
| Northern Philippines | Luzon | Nagsabaran, Zabaran Creek | ANU-15411 | *Batissa children* | 105.03% modern |  | -17 |  |  | unstated | [11: Table 2] |
| Northern Philippines | Luzon | Nagsabaran, Zabaran Creek | ANU-15412 | *Batissa children* | 103.02% modern |  | -14 |  |  | unstated | [11: Table 2] |
| Northern Philippines | Luzon | Andarayan Site |  | Rice husk and stem fragments | 3400 | 125 | -24 | A | Extracted from earthenware jar that contained rice husk and stem portion inclusions in the temper. | red slip | [13:5] |
| Northern Philippines | Luzon | Gaerlan Shell Midden | NUTA2-7938 | Animal bone | 3810 | 30 | - | A | Taxonomic identification of the dating sample and laboratory results are unreported (e.g., specific dating material, sample processing, isotopic results). | red slip | [5: Table 6.1, 14] |
| Northern Philippines | Luzon | Gaerlan Shell Midden | NUTA2-7939 | Animal bone | 3485 | 30 | - | A | Taxonomic identification of the dating sample and laboratory results are unreported (e.g., specific dating material, sample processing, isotopic results). | red slip | [5: Table 6.1, 14] |
| Northern Philippines | Luzon | Gaerlan Shell Midden | NUTA2-7940 | Animal bone | 3665 | 35 | - | A | Taxonomic identification of the dating sample and laboratory results are unreported (e.g., specific dating material, sample processing, isotopic results). | red slip | [5: Table 6.1, 14] |
| Northern Philippines | Luzon | Irigayen | NUTA2-914 | UC | 3025 | 20 | - | C |  | red slip, punctate | [5: Table 6.1, 14] |
| Northern Philippines | Luzon | Irigayen | NUTA2-912 | UC | 2925 | 20 | - | C |  | red slip, punctate | [5: Table 6.1, 14] |
| Northern Philippines | Luzon | Irigayen | NUTA2-913 | UC | 3165 | 25 | - | C |  | red slip, punctate | [5: Table 6.1, 14] |
| Northern Philippines | Luzon | Irigayen | NUTA2-917 | UC | 3185 | 25 | - | C |  | red slip, punctate | [5: Table 6.1, 14] |
| Northern Philippines | Luzon | Lal-lo Shell Midden Site, TU 12S/46E, Level 6, 85 cm | Gak-7048 | Shell (not indicated whether marine or terrestrial) | 3680 | 110 | - | C | Not used in models. | red slip, incised, impressed | [22:90, Table 3] |
| Northern Philippines | Luzon | Callao cave, Layer 4, 40 cm bs | Wk-17010 | UC | 3335 | 34 | - | A |  | red-slip, incised, impressed | [15:Table1] |
| Northern Philippines | Luzon | Dalan Serkot cave, 44 cm bs | Wk-15648 | UC | 3530 | 34 | - | A |  | red-slip, black with incising | [15:Table1] |
| Northern Philippines | Luzon | Dalan Serkot cave, 70 cm bs | Wk-14879 | UC | 6214 | 48 | - | A |  | preceramic | [15:Table1] |
| Northern Philippines | Luzon | Pamittan, Square N59E6, Layer II (lower level) | Gak-17967 | UC | 3390 | 100 | - | C | Association of charcoal and ceramics is unknown given farming field context in valley. | earthenware | [23:124, 132; 24:67] |
| Northern Philippines | Luzon | Pamittan, SquareN59E6, Layer III (71 cm bd) | Gak-17968 | UC | 3810 | 200 | - | C | Association of charcoal and ceramics is unknown given farming field context in valley. | earthenware | [23:124, 132; 24:67] |
| Central Philippines | Negros | Edjek site, Layer IV-V interface, 117-120 cmbs | Beta-1117 | UC | 3475 | 235 | - |  |  | incised, impressed, orange-slip | [18] |
| Central Philippines | Masbate | Bagumbayan Site, Layer IV (lowest spit) | ? | ? | 3510 | 90 | - | C | Dates from the Harwell Low Level Measurements Laboratory (Lab code is HAR). | plain | [16:73] |
| Central Philippines | Masbate | Bagumbayan Site, Layer V (top spit) | ? | ? | 3620 | 100 | - | C | Dates from the Harwell Low Level Measurements Laboratory (Lab code is HAR). | plain | [16:73] |
| Central Philippines | Masbate | Cave 2, 12-18 inch level | L-274 (Lamont laboratory) | UC | 2710 | 100 | - | C | Sherd assemblage also in excavation levels below this date; date is not earliest pottery deposit at site. | incised, impressed | [21:60] |
| Greater Sunda Islands | Borneo/Kalimantan | Liang Abu, Layer 2 | UBA-20839 | UC | 1672 | 21 | -32.5 |  | Site location from map in Simanjuntak [54]. | red-slip, cordmarked | [31] |
| Greater Sunda Islands | Borneo/Kalimantan | Liang Abu, Layer 2 | UBA-20840 | UC | 1524 | 22 | -41.2 |  | Site location from map in Simanjuntak [54]. | red-slip, cordmarked | [31] |
| Greater Sunda Islands | Borneo/Sabah | Bukit Tengkorak, Shelter 1, Square 032, Layer 3, 0-5 cm from top of layer | ANU-5769 | UC | 2700 | 110 | - |  |  | red slip, incised, circle-stamped | [29:Table 1] |
| Greater Sunda Islands | Borneo/Sabah | Bukit Tengkorak, Shelter 1, Square 032, Layer 3, 30-35 cm from top of layer | ANU-5770 | UC | 2330 | 170 | - |  |  | red slip, incised, circle-stamped | [29:Table 1] |
| Greater Sunda Islands | Borneo/Sabah | Bukit Tengkorak, Trench G17, Layer 8, Spit 26, 125-130 cm | Beta-83785 | UC | 5330 | 80 | - | C | Spriggs [55] states association with pottery contested, but reasoning for this is unclear. | red slip, incised, impressed | [56:Table 1] |
| Greater Sunda Islands | Borneo/Sabah | Bukit Tengkorak, Trench G17, Layer 5, Spit 20, 95-100 cm | Beta-83784 | UC | 2650 | 90 | - | C |  | red slip, incised, impressed | [56:Table 1] |
| Greater Sunda Islands | Borneo/Sabah | Bukit Tengkorak, Trench G17, Layer 4, Spit 15, 70-75 cm | Beta-83783 | UC | 2940 | 50 | - | A |  | red slip, incised, impressed | [56:Table 1] |
| Greater Sunda Islands | Borneo/Sabah | Bukit Tengkorak, Trench G17, Layer 3, Spit 11, 50-55 cm | Beta-74448 | *Anadara* sp. | 3190 | 60 | - | C |  | red slip, incised, impressed | [56:Table 1] |
| Greater Sunda Islands | Borneo/Sabah | MAD1, Layer 11a | ANU-2396 | UC | 2650 | 80 | - | C |  | red-slip, impressed | [28] |
| Greater Sunda Islands | Borneo/Sabah | MAD1, Layer 12 top | ANU-2398 | UC | 7390 | 270 | - | C |  | preceramic | [28] |
| Greater Sunda Islands | Borneo/Sabah | MAD1, Layer 12 top | ANU-2397 | UC | 7920 | 370 | - | C |  | preceramic | [28] |
| Greater Sunda Islands | Borneo/Sabah | MAD1, Layer 10 | ANU-2943 | Fresh-water shell | 2700 | 70 | - | C |  | post initial ceramic assemblage | [28] |
| Greater Sunda Islands | Borneo/Sabah | MAD1, Layer 10 base | ANU-2945 | UC | 2020 | 90 | - | C |  | post initial ceramic assemblage | [28] |
| Greater Sunda Islands | Borneo/Sabah | MAD1, Layer 10 | ANU-2395 | UC | 1590 | 150 | - | C |  | post initial ceramic assemblage | [28] |
| Greater Sunda Islands | Borneo/Sarawak | Gua Sireh, Trench F8S, 20-25 cmbs | CAMS-725 | rice husk in sherd | 3850 | 260 | - |  |  | impressed, incised, red-slip | [30:391. Table 1] |
| Greater Sunda Islands | Borneo/Sarawak | Gua Sireh, Trench G8N, Layer 4/6 (approx. 23 cmbs) | ANU-7049 | UC | 3990 | 230 | - |  |  | impressed, incised, red-slip | [30:391. Table 1] |
| Greater Sunda Islands | Borneo/Sarawak | Gua Sireh, Trench G8N, Layer 3 | ANU-7047 | UC | 3220 | 190 | - |  | Determination might be used as a terminus ante quem for estimating date range of initial pottery deposition in excavation trench, but not clear from published sources. | impressed, incised, red-slip | [30:391. Table 1] |
| Greater Sunda Islands | Borneo/Sarawak | Gua Sireh, Trench G8N, Layer 7/8 | ANU-7045 | *Brotia* sp. (freshwater shell) | 5290 | 80 | - |  | Fresh water shell dates from site seem to be effected by carbonate from limestone cave environment [30:388-389]. | impressed, incised, red-slip | [30:391. Table 1] |
| Greater Sunda Islands | Borneo/Sarawak | Gua Sireh, Trench G8N, Layer 9 (top) | ANU-7050 | *Brotia* sp. (freshwater shell) | 5610 | 80 | - |  | Fresh water shell dates from site seem to be effected by carbonate from limestone cave environment [30:388-389]. | impressed, incised, red-slip | [30:391. Table 1] |
| Greater Sunda Islands | Borneo/Sarawak | Niah cave, Burial 110 | Rikagaku Kenkyusho laboratory | human bone | 4990 | 90 | - |  | Sample considered “collagen rich”; taxonomic identification of the dating sample and laboratory results are unreported (e.g., specific dating material, sample processing, isotopic results). | unstated | [40:Table 3] |
| Greater Sunda Islands | Borneo/Kalimantan | Jambu Hilir, test pit w16, 59 cmbs, charcoal concentration beneath sherds | Wk-22009 | UC | 2922 | 45 | - |  | Sherd descriptions not linked to the dated layer; sherds continue to 90 cmbs and not clear why excavation stopped, so deposit might continue deeper. | unstated | [41:124] |
| Greater Sunda Islands | Sulawesi | Minanga Sipakko, Unit M1, 160-170 | ? | UC | 3500 |  | - | C |  | red slip, incised, impressed | [36:60-67] |
| Greater Sunda Islands | Sulawesi | Minanga Sipakko, Unit M1, 170-180 | Wk-17981 | UC | 3343 | 46 | - | A |  | red slip, incised, impressed | [36:60-67] |
| Greater Sunda Islands | Sulawesi | Minanga Sipakko, Unit M3, 155-170 | Wk-14651 | UC | 3446 | 54 | - | A |  | red slip, incised, impressed | [36:60-67] |
| Greater Sunda Islands | Sulawesi | Minanga Sipakko, Unit M3, 220-240 | Wk-14652 | UC | 3082 | 50 | - | A |  | red slip, incised, impressed | [36:60-67] |
| Greater Sunda Islands | Sulawesi | Minanga Sipakko, Unit M4, 200-210 | Wk-14653 | UC | 2881 | 46 | - | C |  | red slip, incised, impressed | [36:60-67] |
| Greater Sunda Islands | Sulawesi | Minanga Sipakko, Unit M4, 250-260 | Wk-14654 | UC | 2996 | 41 | - | C |  | red slip, incised, impressed | [36:60-67] |
| Greater Sunda Islands | Sulawesi | Minanga Sipakko, Unit M5, 170-180 | P3G-05 | UC | 3690 | 160 | - | C | Same lab reference of P3G-05 given for two samples; | red slip, incised, impressed | [36:60-67] |
| Greater Sunda Islands | Sulawesi | Minanga Sipakko, Unit M5, 260-270 | P3G-05 | UC | 4950 | 180 | - | C | Same lab reference of P3G-05 given for two samples; | red slip, incised, impressed | [36:60-67] |
| Greater Sunda Islands | Sulawesi | Minanga Sipakko, Test Pit 2, 155-160 | P3G-97 | UC | 2570 | 110 | - | C | Reported to date “approximate age of uppermost pottery”, but do not consider TAQ, exclude from early pottery models. | red slip, incised, impressed | [36:60-67] |
| Greater Sunda Islands | Sulawesi | Malawa, Spit 6-7, Layer 2 | P3G-06 | UC | 3580 | 130 | - |  | Same lab reference of P3G-06 given for two samples; depth provenience is based on reading of Mahmud 2008, although no specific information is given. | red slip, incised, impressed | [34:125, 35:125] |
| Greater Sunda Islands | Sulawesi | Malawa, Spit 6-7, Layer 2 | P3G-06 | UC | 2710 | 170 | - |  | Same lab reference of P3G-06 given for two samples; depth provenience is based on reading of Mahmud 2008, although no specific information is given. | red slip, incised, impressed | [34:125, 35:125] |
| Greater Sunda Islands | Sulawesi | Mansiri, Trench D, Layer 4, 143 cmbd | Wk-44610 | UC | 2469 | 20 | - |  | Date is from charcoal spatially associated with ceramic. Two other 14C dates from site are from upper pottery layer. | red-slip, incised, circle-stamp, dentate | [38:Table 12.3] |
| Greater Sunda Islands | Sulawesi | Kamassi, K1-K3 trench, Layer 3, 230-240 cm | ANU-35126 | Fresh-water gastropod, *Melanoides* sp. | 1620 | 30 | - |  | Date inverted for depth; unclear if other decorative techniques, e.g., incising, are associated with these lower spits and dates. | red-slip, circle-stamped | [32, 33:Table 1] |
| Greater Sunda Islands | Sulawesi | Kamassi, K1-K3 trench, Layer 3, 240-250 cm | ANU-35127 | Fresh-water gastropod, *Melanoides* sp. | 3225 | 30 | - |  | See ANU-35126. | red-slip, circle-stamped | [32, 33:Table 1] |
| Greater Sunda Islands | Sulawesi | Kamassi, K1-K3 trench, Layer 3, 210-220 cm | ANU-35128 | Fresh-water gastropod, *Melanoides* sp. | 3140 | 30 | - |  | See ANU-35126. | red-slip, circle-stamped | [32, 33:Table 1] |
| Greater Sunda Islands | Sulawesi | Kamassi, K1-K3 trench, Layer 3, 230-240 cm | ANU-36406 | estuarineine shell *Geloina* sp. | 3345 | 40 | - |  | See ANU-35126. | red-slip, circle-stamped | [32, 33:Table 1] |
| Lesser Sunda Islands | Flores Island | Pain Haka Site, Burial 21a, Zone 4 | Wk-36560 | human bone | 2246 | 25 | -15.3 | A | Bone collagen was ultrafiltered with Millipore Amicon Ultra-4 centrifugal filters; all of the analysed individuals reached standard quality control indicators indicative of well-preserved collagen for AMS dating: a wt%N between 11 and 16, a wt%C between 30 and 45, and a C:N ratio range of 3.1–3.5, with a collagen yield ≥0.5% [43:SI). A deltaR of 0+/-0 was used in the calibration. | red-slip, incised, applique | [43:Table 1,] |
| Lesser Sunda Islands | Flores Island | Pain Haka Site, Burial 23, Zone 5, Area ST3 | Wk-36557 | human bone | 2570 | 25 | -14.2 | A | Bone collagen was ultrafiltered with Millipore Amicon Ultra-4 centrifugal filters; all of the analysed individuals reached standard quality control indicators indicative of well-preserved collagen for AMS dating: a wt%N between 11 and 16, a wt%C between 30 and 45, and a C:N ratio range of 3.1–3.5, with a collagen yield ≥0.5% [43:SI). A deltaR of 0+/-0 was used in the calibration. | red-slip, incised, applique | [43:Table 1,] |
| Lesser Sunda Islands | Flores Island | Pain Haka Site, Burial 22, Zone 6, Area ST25 | Wk-36556 | human bone | 2831 | 25 | -16.4 | A | Bone collagen was ultrafiltered with Millipore Amicon Ultra-4 centrifugal filters; all of the analysed individuals reached standard quality control indicators indicative of well-preserved collagen for AMS dating: a wt%N between 11 and 16, a wt%C between 30 and 45, and a C:N ratio range of 3.1–3.5, with a collagen yield ≥0.5% [43:SI). A deltaR of 0+/-0 was used in the calibration. | red-slip, incised, applique | [43:Table 1,] |
| Lesser Sunda Islands | Flores Island | Pain Haka Site, Burial 26, Zone 6, Area XY9 | Wk-36558 | human bone | 2588 | 25 | -13.7 | A | Bone collagen was ultrafiltered with Millipore Amicon Ultra-4 centrifugal filters; all of the analysed individuals reached standard quality control indicators indicative of well-preserved collagen for AMS dating: a wt%N between 11 and 16, a wt%C between 30 and 45, and a C:N ratio range of 3.1–3.5, with a collagen yield ≥0.5% [43:SI). A deltaR of 0+/-0 was used in the calibration. | red-slip, incised, applique | [43:Table 1,] |
| Lesser Sunda Islands | Flores Island | Pain Haka Site, Burial 45, Zone 6, Area V10 | Wk-36559 | human bone | 2548 | 25 | -17.1 | A | Bone collagen was ultrafiltered with Millipore Amicon Ultra-4 centrifugal filters; all of the analysed individuals reached standard quality control indicators indicative of well-preserved collagen for AMS dating: a wt%N between 11 and 16, a wt%C between 30 and 45, and a C:N ratio range of 3.1–3.5, with a collagen yield ≥0.5% [43:SI). A deltaR of 0+/-0 was used in the calibration. | red-slip, incised, applique | [43:Table 1,] |
| Lesser Sunda Islands | Flores Island | Pain Haka Site, Burial 48, Zone 6 | Wk-41599 | human bone | 2532 | 25 | -15.8 | A | Bone collagen was ultrafiltered with Millipore Amicon Ultra-4 centrifugal filters; all of the analysed individuals reached standard quality control indicators indicative of well-preserved collagen for AMS dating: a wt%N between 11 and 16, a wt%C between 30 and 45, and a C:N ratio range of 3.1–3.5, with a collagen yield ≥0.5% [43:SI). A deltaR of 0+/-0 was used in the calibration. | red-slip, incised, applique | [43:Table 1,] |
| Lesser Sunda Islands | Flores Island | Pain Haka Site, Zone 2 | Wk-28995 | UC | 2509 | 25 |  | A | Sample number FLO_7_20011. | red-slip, incised, applique | [43:Table 1,] |
| Lesser Sunda Islands | Flores Island | Pain Haka Site, Zone 4 | Wk-28996 | UC | 2535 | 25 |  | A | Sample number FLO_7_20012. | red-slip, incised, applique | [43:Table 1,] |
| Lesser Sunda Islands | Flores Island | Pain Haka Site, Zone 4 | Wk-28997 | UC | 2725 | 25 |  | A | Sample number FLO_7_20015. | red-slip, incised, applique | [43:Table 1,] |
| Lesser Sunda Islands | Flores Island | Pain Haka Site, Zone 5 | Wk-36711 | UC | 221 | 25 |  | A | Sample number Z5-1_X10. | red-slip, incised, applique | [43:Table 1,] |
| Lesser Sunda Islands | Flores Island | Pain Haka Site, Zone 6 | Wk-36712 | UC | 2784 | 25 |  | A | Sample number Z6-Y10. | red-slip, incised, applique | [43:Table 1,] |
| Moluccas | Morotai | Tanjung Pinang, Layer 1, 5-10 cm | ANU-7778 | marine shell | 3390 | 70 | - |  | Authors label deposit “preceramic?” and note disturbance from burials, but burials date 1000 years more recent than shell and shell dates by depth in perfect order down layer. Bellwood et al. [47:237]: “The pottery belongs to an incised style widespread during the Indonesian Metal Age, dated to as recently as 700 BP (ANU 7784) at the nearby open site of Sambiki Tua (see Fig. 3). Its overall date range at Tanjung Pinang could thus be between c. 2000 and 500 BP.” Claim of 2000 BP for pottery on Morotai is repeated throughout the text, but there is no 14C date associated with this period. | incised, impressed | [47, 57:Table 1] |
| Moluccas | Morotai | Tanjung Pinang, Layer 1, 25-30cm | ANU-7779 | marine shell | 4090 | 70 | - |  | Authors label deposit “preceramic”, sherds at this depth, but none below 30 cm. | incised, impressed | [47, 57:Table 1] |
| Moluccas | Morotai | Tanjung Pinang, Layer 1,30-35cm | ANU-7780 | marine shell | 4720 | 70 | - |  |  | no pottery | [47, 57:Table 1] |
| Moluccas | Morotai | Tanjung Pinang, Layer 1, 50-55cm | ANU-7781 | marine shell | 5390 | 70 | - |  |  | no pottery | [47, 57:Table 1] |
| Moluccas | Gebe | Um Kapat Papo (UKP) Layer 2, 15-20 cm (depth measured from zero at start of Layer 2) | ANU-9316 | marine shell | 2030 | 60 | - |  |  | incised | [47] |
| Moluccas | Gebe | Um Kapat Papo (UKP) Layer 3, 5-15 cm (depth measured from zero at start of Layer 3) | ANU-9317 | marine shell | 4830 | 70 | - |  | Authors label this pre-ceramic, but there are sherds in Layer 3 (67, 0-10 cm; 7, 10-20 cm) [47:Table 4]. | incised | [47] |
| Moluccas | Gebe | Golo, | ANU-9448 | UC | 3230 | 180 | - |  |  | no pottery | [47] |
| Moluccas | Halmahera | Siti Nafisah, Layer A, spit 1, 0-4 cmbs | ANU-7785 | estuarine shell | 2540 | 70 | - |  | One hundred thirty-foursherds in top seven cm. Authors state: “Layer A at the surface of the site produced incised and red-slipped pottery dated to c. 2000-1500 BP” but no dates return this range. Date at very top is 2540 +/- 70 , while a date at c. 10 cm is 3410 +/- 70. Excluded from analysis as unclear which determinations relate to pottery-bearing deposits | incised, red-slip | [47] |
| Moluccas | Halmahera | Siti Nafisah, Layer B, spit 1, 7-13 cmbs | ANU-7786 | estuarine shell | 3410 | 70 | - |  | Nine sherds at this depth; excluded from analysis as unclear which determinations relate to pottery-bearing deposits. | incised, red-slip | [47] |
| Moluccas | Halmahera | Siti Nafisah, Layer C, spit 3, 30-35 cmbs | ANU-7787 | estuarine shell | 4690 | 120 | - |  | One sherd at this depth; excluded from analysis as unclear which determinations relate to pottery-bearing deposits. | incised, red-slip | [47] |
| Moluccas | Kayoa | Uattamdi, Layer D, 0-15 cm from top of layer | ANU-9323 | marine shell | 3260 | 70 | - |  |  | incised, red-slip | [47] |
| Moluccas | Kayoa | Uattamdi, Layer D, 15-20 cm from top of layer | ANU-7776 | marine shell | 3440 | 110 | - |  |  | incised, red-slip | [47] |
| Moluccas | Kayoa | Uattamdi, Layer E | ANU-3530 | marine shell | 3530 | 70 | - |  |  | incised, red-slip | [47] |
| Moluccas | Pulau Ay | PA1 Unit 3, Layer 6B, 236 cm | Beta-235453 | UC | 3010 | 40 | -24.8 | A |  | incised, red-slip. | [46:Table 1] |
| Moluccas | Pulau Ay | PA1 Unit 3, Layer 6A, 250 cm | Beta-240739 | UC | 3190 | 40 | -28.5 | A |  | incised, red-slip. | [46:Table 1] |
| Moluccas | Pulau Ay | PA1 Unit 3, Layer 4B, 195 cm | Beta-240738 | UC | 3010 | 40 | -24.5 | A |  | red slip | [46:Table 1] |
| Moluccas | Pulau Ay | PA1 Unit 3, Layer 3I 175-185 cm | Beta-304478 | UC | 2940 | 40 | -26.4 | A |  | red slip | [46:Table 1] |
| Moluccas | Pulau Ay | PA1 Unit 3, Layer 3I, 164 cm | Beta-302405 | UC | 2880 | 30 | -24.1 | A |  | red slip | [46:Table 1] |
| Moluccas | Pulau Ay | PA1 Unit 3, Layer 3E, 124 cm | Beta-302404 | UC | 2780 | 30 | -23.7 | A |  | red slip | [46:Table 1] |
| Moluccas | Pulau Ay | PA1 Unit 3, Layer 3C, 105 cm | Beta-235454 | UC | 2460 | 40 | -23.5 | A |  | red slip, circle-stamp | [46:Table 1] |
| Moluccas | Talaud Islands | LTM site, PQ sector, base Layer 4 | ANU-1515 | UC | 4030 | 80 | - |  |  | red-slip, incised, impressed | [48:261] |
| Moluccas | Talaud Islands | LTM site, trench H9, top Layer 3 | ANU-1717 | *Turbo* sp. | 4860 | 130 | - |  |  | no pottery | [48:261] |
| Lesser Sundas | East Timor | Lena Hara Cave, square F, spit 16, 61-68 cm | ANU-12029 | charcoal on sherd | 3200 | 240 | -24 |  | Sample is described as “charred convex surface of one sherd”. | red-slip, plain | [53:Table 2] |
| Lesser Sundas | East Timor | Lena Hara Cave, square F, spit 16, 61-68 cm | ANU-12041 | *T. niloticusus* | 3850 | 70 | - |  |  | red-slip, plain | [53:Table 2] |
| Greater Sunda Islands | Java | Keplek Cave, B5, 18-30 cm | P3G-1998 | UC | 3260 | 110 | - |  |  | earthenware | [37] |
| Greater Sunda Islands | Java | Braholo Cave, L8, 64-80 cm | P3G-1999 | UC | 3050 | 100 | - |  |  | earthenware | [37] |
| Greater Sunda Islands | Sulawesi | Topogaro 2, TA-1, spit 11 | TKA-17404 | human tooth | 1900 | 20 | 91.9 |  | Taxonomic identification of the dating sample and laboratory results are unreported (e.g., specific dating material, sample processing, isotopic results). | red-slip, incised, dentate | [39:Table 1] |
| Greater Sunda Islands | Sulawesi | Topogaro 2, TB-1, spit 6 | TKA-17035 | UC | 2274 | 19 | - |  |  | red-slip, incised, dentate | [39:Table 1] |
| Moluccas | Talaud Islands | Leang Tahuna, Square I5, spit 8 | OZD-771 | *Tridacna* sp. adze | 4310 | 50 | - |  | Author states citing personal communication from Bellwood that this may be “old shell”. | red-slip | [58:440] |
| Moluccas | Talaud Islands | Leang Tuwo Mane‘e, Square K17, spit14 | ANU-10209 | *Turbo* sp. | 3690 | 70 | - |  | Sherds continue for five spits below this date, although author suggests date is still related to start of ceramic deposition. | red-slip | [58:440] |
| Mussau | Eloaua | ECA | GX-5498 | UC | 3030 | 180 | - |  | From Egloff's original excavations at the site. | red-slip, incised, circle-stamp, and dentate | [59:Table 1] |
| Mussau | Eloaua | ECA, Area A; 1985 Unit W229N100, Level 9 | ANU-5085 | *Hyotissa* sp. | 3130 | 80 | 0.0 (estimated) |  | Not included in Denham et al.[59]. Calibrated here with a ΔR of -434±179, the Marine20-adjusted value from Petchey & Ulm [61]. | red-slip, incised, circle-stamp, and dentate | [60:224] |
| Mussau | Eloaua | ECA, Area A; 1985 Unit W228N102, Level 3 | ANU-5084 | *Tridacna* sp. | 3190 | 80 | 0.0 (estimated) |  | Not included in Denham et al. [59]. Calibrated here with a ΔR of -434±179, the Marine20-adjusted value from Petchey & Ulm [61]. | red-slip, incised, circle-stamp, and dentate | [60:223] |
| Mussau | Eloaua | ECA, Airfield Transects; 1985  TP-9, Unit W400N72, Level 6, 40-70 cm bs | ANU-5080 | UC | 3260 | 90 | -24.0 (estimated) |  |  | red-slip, incised, circle-stamp, and dentate | [60:223] |
| Mussau | Eloaua | ECA, W250 Transect; Unit W250N90, Level 2 | Beta-30676 | *Turbo marmoratus* | 3590 | 110 | 1.9 |  | Not included in Denham et al. [59]; Kirch argues that *Turbo*'s habitat preference along the outer reef warrants an "open ocean" ΔR of 0; cf. Petchey & Ulm's [61] ΔR value. Calibrated here with a ΔR of -434±179, the Marine20-adjusted value from Petchey & Ulm [61]. | red-slip, incised, circle-stamp, and dentate | [60:227] |
| Mussau | Eloaua | ECA, Area B; 1985 Unit W199N151, Post B2 | ANU-5791 | *Intsia bijuga* (outer 2 cm, including bark) | 2930 | 80 | -24.0 (estimated) |  | Same stilt house as ANU-5790, different post. | red-slip, incised, circle-stamp, and dentate | [60:226] |
| Mussau | Eloaua | ECA, Area B; 1985 combined sample from Unit W200N150, Levels 12 & 13, Zone C1 | ANU-5079 | UC | 2840 | 115 | -24.0 (estimated) |  | Post-dates the stilt house. | red-slip, incised, circle-stamp, and dentate | [60:225] |
| Mussau | Eloaua | ECA, Area B; Unit W198N145, Post B30, Level 7, Zone C3 | Beta-20452 | Unid. wood (outer 2 cm, including bark) | 3050 | 70 | -24.0 (estimated) |  |  | red-slip, incised, circle-stamp, and dentate | [60:227] |
| Mussau | Eloaua | ECA, Unit W200 Transect (TP18), Layer III, Level 9, ~130 cm bs | Beta-20451 | *Cocos nucifera* (endocarp) | 2950 | 70 | -24.0 (estimated) |  |  | red-slip, incised, circle-stamp, and dentate | [60:224] |
| Mussau | Eloaua | ECA, Area B; Unit W200N150, Post B1 | ANU-5790 | *Intsia bijuga* (outer 2 cm, including bark) | 2950 | 80 | -24.0 (estimated) |  | Same stilt house as ANU-5791, different post. | red-slip, incised, circle-stamp, and dentate | [60:226] |
| Mussau | Eloaua | ECA, Area B; Unit W200N151, Level 11, Zone C3 | ANU-5081 | *Tridacna gigas* | 3010 | 80 | 0.0 (estimated) |  | Representing deposition around stilt house; Not included in Denham et al. [59]. Calibrated here with a ΔR of -434±179, the Marine20-adjusted value from Petchey & Ulm [61]. | red-slip, incised, circle-stamp, and dentate | [60:225] |
| Mussau | Eloaua | ECA, Area B; Unit W201N149, Level 12, Zone C3 | ANU-5082 | *Hyotissa hyotis* | 2950 | 80 | 0.0 (estimated) |  | Representing deposition around stilt house; Not included in Denham et al. [59]. Calibrated here with a ΔR of -434±179, the Marine20-adjusted value from Petchey & Ulm [61]. | red-slip, incised, circle-stamp, and dentate | [60:226] |
| Mussau | Eloaua | ECA, Area C; Unit W249N188, Level 2 | Beta-30674 | *Hippopus hippopus* | 3110 | 70 | 1.0 |  | Kirch notes that this date and Beta-30675 are anomalously early for Area C and may represent midden discarded in the area previously; Not included in Denham et al. [59]. Calibrated here with a ΔR of -434±179, the Marine20-adjusted value from Petchey & Ulm [61]. | red-slip, incised, circle-stamp, and dentate | [60:229] |
| Mussau | Eloaua | ECA, Area C; Unit W249N188, Level 4 | Beta-30675 | *Tridacna derasa* | 3110 | 80 | 1.9 |  | Kirch notes that this date and Beta-30674 are anomalously early for Area C and may represent midden discarded in the area previously; Not included in Denham et al. [59]. Calibrated here with a ΔR of -434±179, the Marine20-adjusted value from Petchey & Ulm [61]. | red-slip, incised, circle-stamp, and dentate | [60:230] |
| Mussau | Eloaua | ECA, Area C; Unit W250N188, Post C3 | Beta-30686 | *Diospyros* sp. | 2850 | 70 | -24.5 |  | Based on ceramics and geomorphology, Kirch argues occupation of this area post-dates the earliest occupation of Areas A & B. | red-slip, incised, circle-stamp, and dentate | [60:230] |
| Mussau | Eloaua | ECA, W250 transect; Unit W250N100, Level 2 | Beta-30677 | *Spondylus* sp. | 3170 | 70 | 1.2 |  | Not included in Denham et al. [59]. Calibrated here with a ΔR of -434±179, the Marine20-adjusted value from Petchey & Ulm [61]. | red-slip, incised, circle-stamp, and dentate | [60:227] |
| Mussau | Eloaua | ECA, W250 transect; Unit W250N110, Level 15 | Beta-30679 | *Tridacna gigas* | 3080 | 70 | 2.3 |  | Not included in Denham et al. [59]. Calibrated here with a ΔR of -434±179, the Marine20-adjusted value from Petchey & Ulm [61]. | red-slip, incised, circle-stamp, and dentate | [60:228] |
| Mussau | Eloaua | ECA, W250 transect; Unit W250N110, Level 4 | Beta-30678 | *Chama* sp. shell | 3190 | 80 | 2.1 |  | Not included in Denham et al. [59]. Calibrated here with a ΔR of -434±179, the Marine20-adjusted value from Petchey & Ulm [61]. | red-slip, incised, circle-stamp, and dentate | [60:228] |
| Mussau | Eloaua | ECA, W250 transect; Unit W250N120, Level 6 | Beta-30680 | *Chama* sp. shell | 3320 | 80 | 2.8 |  | Kirch flags as a "rogue" date; Not included in Denham et al. [59]. Calibrated here with a ΔR of -434±179, the Marine20-adjusted value from Petchey & Ulm [61] | red-slip, incised, circle-stamp, and dentate | [60:228] |
| Mussau | Eloaua | ECA, W250 transect; Unit W250N120, Level 9, wooden post | Beta-30681 | Unid. wood | 2860 | 60 | -30.5 |  |  | red-slip, incised, circle-stamp, and dentate | [60:228] |
| Mussau | Eloaua | ECA, W250 transect; Unit W250N140, Level 6, wooden post | Beta-30682 | Unid. wood | 2970 | 50 | -28.1 |  |  | red-slip, incised, circle-stamp, and dentate | [60:229] |
| Mussau | Eloaua | ECA, W250 transect; Unit W250N150, Level 7 | Beta-30683 | *Hippopus hippopus* | 3140 | 80 | 2.7 |  | Not included in Denham et al. [59]. Calibrated here with a ΔR of -434±179, the Marine20-adjusted value from Petchey & Ulm [61]. | red-slip, incised, circle-stamp, and dentate | [60:229] |
| Mussau | Eloaua | ECA, W250 transect; Unit W250N170, Level 3, wooden stake | Beta-30684 | Unid. wood | 3100 | 110 | -28.6 |  |  | red-slip, incised, circle-stamp, and dentate | [60:229] |
| Mussau | Eloaua | ECB, Etakosarai; 1985 transect Unit 1, Level 1 | ANU-5086 | *Hyotissa hyotis* | 3120 | 80 | 0.0 (estimated) |  | Three determinations from ECB believed to relate to a single, short-duration Lapita occupation; not included in Denham et al. [59]. Calibrated here with a ΔR of -434±179. | red-slip, incised, circle-stamp, and dentate | [60:231] |
| Mussau | Eloaua | ECB, Etakosarai; 1985 transect Unit 1, Level 2 | ANU-5087 | *Hyotissa hyotis* | 3150 | 80 | 0.0 (estimated) |  | Three determinations from ECB believed to relate to a single, short-duration Lapita occupation; not included in Denham et al. [59]. Calibrated here with a ΔR of -434±179. | red-slip, incised, circle-stamp, and dentate | [60:231] |
| Mussau | Eloaua | ECB, Etakosarai; 1986 transect Unit 9, Level 5, 71 cm bs | Beta-20453 | UC | 3200 | 70 | -24.0 (estimated |  | Three determinations from ECB believed to relate to a single, short-duration Lapita occupation. | red-slip, incised, circle-stamp, and dentate | [60:231] |
| Mussau | Emirau | EQS, Tamuarawai; Test Pit 1, Layer 4 | Wk-21349 | UC | 3044 | 31 | -23.4±0.2 |  | Depositional context interpreted as having formed in a low energy lagoonal environment. | red-slip, incised, circle-stamp, and dentate | [49:Table 1] |
| Mussau | Emirau | EQS, Tamuarawai; Test Pit 2, Layer 4 | Wk-21345 | UC | 2917 | 31 | -27.4±0.2 |  | Depositional context interpreted as initial build-up of calcareous beach, post-dating lagoonal occupation. | red-slip, incised, circle-stamp, and dentate | [49:Table1] |

*: UC = unidentified charcoal

†: A = accelerator mass spectrometry, C = conventional, blank cell = unstated
