# Supplementary material for: The first quantitative assessment of radiocarbon chronologies for initial pottery in Island Southeast Asia supports multi-directional Neolithic dispersal
Source: PLoS One. 2021 Jun 2;16(6):e0251407. doi: 10.1371/journal.pone.0251407 (PMC8171956; doi:10.1371/journal.pone.0251407)
Supplement: Attachment — Submitted filename: Cochrane_etal_ceramics_SI_Plos1 comments.docx [file pone.0251407.s002.docx]

Supporting Information for:

Bayesian radiocarbon chronologies for initial pottery in Island Southeast Asia support multi-directional Neolithic dispersal

Ethan E. Cochrane^1¶^*, Timothy M. Rieth^2¶^, Darby Filimoehala^2^

^1^Anthropology, The University of Auckland, Auckland, New Zealand

^2^International Archaeological Research Institute Inc., Honolulu, USA

* Corresponding author

E-mail: e.cochrane@auckland.ac.nz (EC)

^¶^These authors contributed equally to the work.

**Model descriptions and Oxcal code**

The model descriptions, results, and code are presented in the same order as the results discussed in the main text. For many site deposits two or more models were created and the codes for these are presented below. It is important to note that most of the models are hindered by one or more issues: too few radiocarbon determinations, unclear stratigraphic information presented in publications, uncertain localized correction values, and the possibility of inbuilt age. Within the constraints of these limitations, we justify our preferred models with the caveat that additional dates and other information (e.g., correction values) should improve the chronological estimates. The results of our non-preferred models do not significantly change our conclusions. The inclusion of models with determinations on residue

**Batanes Islands**

Eleven single- or multi-phase models were created for site deposits on Batan [1-5], Itbayat [6], and Sabtang Islands [7] (S1 Fig). Determinations from these sites and three other site deposits or excavation areas (S3 Table that produced individual radiocarbon ages (Mahatao Town Septic Tank [3, 4] and Tayid [2]) were included in single-phase models for each respective island.

Two island-scale models were created for Batan Island: one model includes determinations from unidentified charcoal and residue on sherds, while another model excludes the determinations from the sherd residue. The second model evaluates the possibility that sherd residue determinations are older than stratigraphically associated charcoal ages and are potentially problematic [8]. For those sites that produced radiocarbon determinations from stratified deposits, multi-phase models were created.

Dating samples were unidentified wood charcoal, residue on sherds, and one sample of “resin” coating a sherd. Four Batanes radiocarbon determinations were excluded from the models: ANU-11709 (unidentified charcoal; 200±360 BP) is anomalously young and must be an intrusive specimen [2: 71, Table 5.1]; OZH-776 (resin; 5790±150 BP) was identified as being anomalously old by Bellwood and Dizon [2:70] and there is a question about what comprises the resin and how suitable it is for dating of the associated ceramic; and Gif-6575 (unidentified charcoal; 2310±80 BP) from the Basco site on Batan Island [9:13] was excluded because it cannot be clearly associated with a ceramic deposit.

**Naidi Site, Batan Island**

The initial appearance of ceramics at the Naidi site on Batan Island is estimated to have occurred sometime between *5916-1609 cal BP (95.4%) or 5906-1940 cal BP (68.2%)*. The single-phase model contains three radiocarbon determinations obtained from unidentified charcoal. Such a small number of determinations results in an estimate with poor precision.

Plot()

{

Outlier_Model("Charcoal",Exp(1,-10,0),U(0,3),"t");

Sequence("Batan Naidi")

{

Boundary("start");

Phase("Naidi")

{

R_Date("ANU-11695",2620,30)

{

Outlier(1);

};

R_Date("ANU-11708",2240,140)

{

Outlier(1);

};

R_Date("ANU-11694",1590,210)

{

Outlier(1);

};

};

Boundary("end");

};

};

**Payaman Site, Batan Island**

The initial appearance of ceramics at the Payaman site on Batan Island is estimated to have occurred sometime between *4928-1542 cal BP (95.4%) or 4928-1769 cal BP (68.2%)*. The single-phase model contains two radiocarbon determinations obtained from unidentified charcoal. Such a small number of determinations results in an estimate with poor precision.

Plot()

{

Outlier_Model("Charcoal",Exp(1,-10,0),U(0,3),"t");

Sequence("Batan Payaman")

{

Boundary("start");

Phase("Payaman")

{

R_Date("Wk-13092",1988,47)

{

Outlier(1);

};

R_Date("ANU-12068",1486,185)

{

Outlier(1);

};

};

Boundary("end");

};

};

**Sunget Site, Batan Island**

A multi-phase model with contiguous ordering based on stratigraphy for an excavation in the main terrace at the Sunget site, Batan Island, failed as the order could not be resolved; the determinations are inverted. A single-phase model including determinations from the main and top terrace excavations results in an estimate for the appearance of ceramics at Sunget sometime between *4451-2897 cal BP (95.4%) or 3505-3010 cal BP (68.2%)*. The model contains five radiocarbon determinations, three of which were obtained from unidentified charcoal (ANU-11693, Wk-15649, and ANU-11707) with the other two from charred residue on ceramic sherds (Wk-14640 and ANU-11817).

Plot()

{

Outlier_Model("General",T(5),U(0,4),"t");

Outlier_Model("Charcoal",Exp(1,-10,0),U(0,3),"t");

Sequence("Batan Sunget")

{

Boundary("start");

Phase("Sunget")

{

R_Date("Wk-14640",2915,49)

{

Outlier("General",0.05);

};

R_Date("ANU-11817",2910,190)

{

Outlier("General",0.05);

};

R_Date("ANU-11693",2630,30)

{

Outlier("Charcoal",1);

};

R_Date("Wk-15649",2383,35)

{

Outlier("Charcoal",1);

};

R_Date("ANU-11707",2000,140)

{

Outlier("Charcoal",1);

};

};

Boundary("end");

};

};

**Batan Island**

The determinations from the Naidi, Payaman, and Sunget models were combined with individual ages from the Mahatao and Tayid sites in a single-phase, island-scale model for Batan. At this geographic scale, the appearance of pottery is estimated to have occurred sometime between *3681-2899 cal BP (95.4%) or 3321-3004 cal BP (68.2%)*. This estimate is significantly younger and more precise than the site-scale estimates due to the increased number of determinations included in the model. The dated Batan Island pottery assemblages include red-slip and circle-stamp surface treatments [10: Figure 6.13].

Plot()

{

Outlier_Model("General",T(5),U(0,4),"t");

Outlier_Model("Charcoal", Exp(1,-10,0), U(0,3), "t");

Sequence("Batan Island")

{

Boundary("start");

Phase("Batan")

{

R_Date("Wk-14640",2915,49)

{

Outlier("General",0.05);

};

R_Date("ANU-11817",2910,190)

{

Outlier("General",0.05);

};

R_Date("ANU-11693",2630,30)

{

Outlier("Charcoal",1);

};

R_Date("ANU-11695",2620,30)

{

Outlier("Charcoal",1);

};

R_Date("Wk-15649",2383,35)

{

Outlier("Charcoal",1);

};

R_Date("ANU-11708",2240,140)

{

Outlier("Charcoal",1);

};

R_Date("ANU-11710",2090,60)

{

Outlier("Charcoal",1);

};

R_Date("ANU-11707",2000,140)

{

Outlier("Charcoal",1);

};

R_Date("Wk-13092",1988,47)

{

Outlier("Charcoal",1);

};

R_Date("ANU-12069",1842,215)

{

Outlier("General",0.05);

};

R_Date("ANU-11694",1590,210)

{

Outlier("Charcoal",1);

};

R_Date("ANU-12068",1486,185)

{

Outlier("Charcoal",1);

};

};

Boundary("end");

};

};

**Batan Island without residue determinations**

The determinations from unidentified charcoal from the Naidi, Payaman, and Sunget models were combined with individual ages from the Mahatao and Tayid sites in a single-phase, island-scale model for Batan; this model excludes three determinations obtained from residue on sherds (Wk-14640, ANU-11817, and ANU-12069). The appearance of pottery is estimated to have occurred sometime between *3410-2575 cal BP (95.4%) or 2978-2734 cal BP (68.2%)*. This estimate is younger than the island-scale model that includes the sherd residue determinations, and at 95.4% probability the estimate is significantly less precise. It is apparent that the determinations from sherd residue return older calibrated dates, which affect the modeled results.

Plot()

{

Outlier_Model("Charcoal", Exp(1,-10,0), U(0,3), "t");

Sequence("Batan Island")

{

Boundary("start");

Phase("Batan")

{

R_Date("ANU-11693",2630,30)

{

Outlier(1);

};

R_Date("ANU-11695",2620,30)

{

Outlier(1);

};

R_Date("Wk-15649",2383,35)

{

Outlier(1);

};

R_Date("ANU-11708",2240,140)

{

Outlier(1);

};

R_Date("ANU-11710",2090,60)

{

Outlier(1);

};

R_Date("ANU-11707",2000,140)

{

Outlier(1);

};

R_Date("Wk-13092",1988,47)

{

Outlier(1);

};

R_Date("ANU-11694",1590,210)

{

Outlier(1);

};

R_Date("ANU-12068",1486,185)

{

Outlier(1);

};

};

Boundary("end");

};

};

**Anaro Hilltop, Itbayat Island**

Four single-phase models were created for the Anaro Hilltop site on Itbayat Island. The initial Area 3B model failed because of one inverted date (OZH-695); a second iteration of the model with OZH-695 removed was successful. Estimates for the initial occurrence of ceramics at the site are spatially variable: *5448-2879 cal BP (95.4%) or 5448-2889 cal BP (68.2%)* at Area 3; *3908-2410 cal BP (95.4%) or 3908-2437 cal BP (68.2%)* at Area 3A; and *2145-1277 cal BP (95.4%) or 1545-1309 cal BP (68.2%)* at Area 3B. The determinations used in the Area 3, Area 3A, and Area 3B models were combined with OZH-695 and a single radiocarbon determination from Area 2A in a single-phase model for the Anaro Hilltop site as a whole (nine determinations, all obtained from charred residue on ceramic sherds). The site-scale model estimates the onset of ceramics sometime between *3822-2751 cal BP (95.4%) or 3139-2808 cal BP (68.2%)*. This is our preferred model since it has the largest number of radiocarbon determinations, which should positively affect accuracy and precision. However, as noted above for Batan Island, dating charred residue may require a correction value or otherwise produce inaccurate dates. The pottery associated with the Anaro Hilltop age estimate includes circle-stamp surface treatment [10].

Area 3

Plot()

{

Outlier_Model("General",T(5),U(0,4),"t");

Sequence("Itbayat_Anaro_Area3")

{

Boundary("start Area 3");

Phase("Anaro_Area3")

{

R_Date("OZH-774",2770,50)

{

Outlier("General",0.05);

};

R_Date("Wk-14645",1360,39)

{

Outlier("General",0.05);

};

};

Boundary("end");

};

};

Area 3A

Plot()

{

Outlier_Model("General",T(5),U(0,4),"t");

Sequence("Itbayat_Anaro_Area3A")

{

Boundary("start");

Phase("Anaro_Area3A")

{

R_Date("OZI-693",2475,45)

{

Outlier("General",0.05);

};

R_Date("OZI-692",2095,45)

{

Outlier("General",0.05);

};

};

Boundary("end");

};

};

Area 3B

Plot()

{

Outlier_Model("General",T(5),U(0,4),"t");

Sequence("Itbayat_Anaro_Area3B")

{

Boundary("start");

Phase("Anaro_Area3B")

{

R_Date("OZI-697",1510,60)

{

Outlier("General",0.05);

};

R_Date("OZI-696",1375,45)

{

Outlier("General",0.05);

};

R_Date("OZI-694",1280,45)

{

Outlier("General",0.05);

};

};

Boundary("end");

};

};

Anaro Hilltop Site

Plot()

{

Outlier_Model("General",T(5),U(0,4),"t");

Sequence("Itbayat_Anaro_Hilltop")

{

Boundary("start");

Phase("Anaro_Hilltop")

{

R_Date("OZH-774",2770,50)

{

Outlier("General",0.05);

};

R_Date("OZH-693",2475,45)

{

Outlier("General",0.05);

};

R_Date("OZH-692",2095,45)

{

Outlier("General",0.05);

};

R_Date("OZH-695",2080,45)

{

Outlier("General",0.05);

};

R_Date("Wk-14643",1876,41)

{

Outlier("General",0.05);

};

R_Date("OZH-697",1510,60)

{

Outlier("General",0.05);

};

R_Date("OZH-696",1375,35)

{

Outlier("General",0.05);

};

R_Date("Wk-14645",1360,39)

{

Outlier("General",0.05);

};

R_Date("OZH-694",1280,45)

{

Outlier("General",0.05);

};

};

Boundary("end");

};

};

**Torongan Cave, Itbayat Island**

The initial appearance of ceramics at the Torongan Cave site on Itbayat Island is estimated to have occurred sometime between *6368-4163 cal BP (95.4%) or 6368-4181 cal BP (68.2%)*. The single-phase model contains two radiocarbon determinations obtained from charred residue on ceramic sherds. Such a small number of determinations results in an estimate with poor precision. Red-slipped pottery is associated with these determinations at the base of the cultural layer [6: Table 2.1]

Plot()

{

Outlier_Model("General",T(5),U(0,4),"t");

Sequence("Itbayat_Torongan_Cave")

{

Boundary("start");

Phase("Torongan")

{

R_Date("OZH-771",3860,70)

{

Outlier("General",0.05);

};

R_Date("Wk-14642",3320,40)

{

Outlier("General",0.05);

};

};

Boundary("end");

};

};

**Itbayat Island**

The determinations from the Anaro Hilltop and Torongan Cave models were combined in a single-phase, island-scale model for Itbayat Island. At this scale, the appearance of pottery is estimated to have occurred sometime between *5352-3506 cal BP (95.4%) or 4697-4159 cal BP (68.2%)*. The two Torongan Cave determinations seem to be driving the estimate.

Plot()

{

Outlier_Model("General",T(5),U(0,4),"t");

Sequence("Itbayat_Island")

{

Boundary("start");

Phase("Itbayat")

{

R_Date("OZH-771",3860,70)

{

Outlier("General",0.05);

};

R_Date("Wk-14642",3320,40)

{

Outlier("General",0.05);

};

R_Date("OZH-774",2770,50)

{

Outlier("General",0.05);

};

R_Date("OZH-693",2475,45)

{

Outlier("General",0.05);

};

R_Date("OZH-692",2095,45)

{

Outlier("General",0.05);

};

R_Date("OZH-695",2080,45)

{

Outlier("General",0.05);

};

R_Date("Wk-14643",1876,41)

{

Outlier("General",0.05);

};

R_Date("OZH-697",1510,60)

{

Outlier("General",0.05);

};

R_Date("OZH-696",1375,35)

{

Outlier("General",0.05);

};

R_Date("Wk-14645",1360,39)

{

Outlier("General",0.05);

};

R_Date("OZH-694",1280,45)

{

Outlier("General",0.05);

};

};

Boundary("end");

};

};

**Savidug Dune, Sabtang Island**

The Savidug Dune site is the sole dated deposit for Sabtang Island, and thus the site and island models are the same. Bellwood and Dizon’s [7] presentation of the contexts for the radiocarbon determinations contains discrepancies in places (compare text on pages 55 and 57 with Table 5.1 and Figure 4.13). Regardless of the discrepancies in the layer designations and depths for the samples, they appear to relate to the basal ceramic deposit. Therefore, they were included in a single-phase model. Based on the four determinations from Savidug, the initial appearance of pottery is estimated to have occurred sometime between *4568-2893 cal BP (95.4%) or 3343-2947 cal BP (68.2%)*. The determinations are from unidentified charcoal (Wk-19711, Wk-21808, and Wk-21809) and charred residue on a ceramic sherd (Wk-21810). The Savidug date estimates are associated with red-slip, circle-stamp, and applique pottery [see e.g., 10: Figure 6.16]

Plot()

{

Outlier_Model("General",T(5),U(0,4),"t");

Outlier_Model("Charcoal", Exp(1,-10,0), U(0,3), "t");

Sequence("Sabtang_Savidug")

{

Boundary("start");

Phase("Basal ceramic layer")

{

R_Date("Wk-21810",2870,30)

{

Outlier("General",0.05);

};

R_Date("Wk-19711",2828,37)

{

Outlier("Charcoal",1);

};

R_Date("Wk-21809",2416,30)

{

Outlier("Charcoal",1);

};

R_Date("Wk-21808",2146,30)

{

Outlier("Charcoal",1);

};

};

Boundary("end");

};

};

**Philippines**

Ceramic-bearing archaeological deposits on the Philippine islands of Luzon, Masbate, and Negros have reported radiocarbon determinations, though the majority of this corpus is from excavations on Luzon. Seven single- or multi-phase models were created for site deposits from Andarayan [11], Irigayen [12], and Nagsabaran [5, 13-15] on Luzon Island (S2 Fig). Determinations from these sites and two other site deposits that produced individual radiocarbon ages (Callao Cave and Dalan Serkot [16]) were included in two single-phase models for the island. For Nagsabaran, the only site with multiple radiocarbon determinations reported by strata or depth, multi-phase models were created for individual excavation units; a single-phase model for the site as a whole was also created.

Philippine dating samples are unidentified wood charcoal, unidentified animal bone, unidentified shell, freshwater shell (*Batissa childreni*), pig (*Sus scrofa/verrucosus*), rice husk and stem fragments, and unreported material [11:5, 12:30, 13, 14, 16-19, 20:691, 21:78, 22]. The determinations obtained from unidentified animal bone (Wk-19712, 2504±35; NUTA2-7938, 3810±30 BP; NUTA2-7939, 3485±30 BP; NUTA2-7940, 3665±35 BP) [5:159, Table 7.1, 12:30], unidentified shell (Gak-7048, 3680±110 BP) [23:80], and pig (Wk-23397, 3940±40 BP) [20:691, 21:78] were excluded. Isotopic data for the bone samples is not reported and therefore it is unknown whether a mixed atmospheric and marine calibration is warranted (likely for the pig specimen). It is unreported whether the unidentified shell sample is from a terrestrial, freshwater, or marine invertebrate. In addition, two determinations obtained from contexts where the associations with ceramics are ambiguous (Gak-17967, 3390±100 BP; Gak-17968, 3810±200 BP) [24:132, 25:67] were excluded.

One iteration, each, of the Nagsabaran site-scale and Luzon Island models was run with a single freshwater shell-derived determination using IntCal13; other iterations excluded the shell determination. Though the possibility of a freshwater reservoir effect has not been thoroughly evaluated [contra 14], the freshwater shell determination was included to evaluate its effect on model results.

Individual determinations on unidentified charcoal from the Edjeck Site, Negros Island [19] and Cave 2 on Masbate Island [22] were not modeled since they represent the sole dates for each island. The two determinations from the Bagumbayan Site, Masbate Island [17] are on unreported material and are excluded.

**Andarayan Site, Luzon Island**

The initial appearance of ceramics at the Andarayan site on Luzon is estimated to have occurred sometime between *7148-3388 cal BP (95.4%)* or *4414-3450 cal BP (68.2%).* The single-phase model contains two radiocarbon determinations obtained from unidentified charcoal (SFU-86) and rice husk and stem (unreported laboratory number). Such a small number of determinations results in an estimate with poor precision. Red-slip pottery is associated with this date estimate [26].

Plot()

{

Outlier_Model("General",T(5),U(0,4),"t");

Outlier_Model("Charcoal", Exp(1,-10,0), U(0,3), "t");

Sequence("Luzon_Andarayan")

{

Boundary("start");

Phase("Andarayan")

{

R_Date("Unreported",3400,125)

{

Outlier("General",0.05);

};

R_Date("SFU-86",3240,160)

{

Outlier("Charcoal",1);

};

};

Boundary("end");

};

};

**Irigayen Site, Luzon Island**

The initial appearance of ceramics at the Irigayen site on Luzon is estimated to have occurred sometime between *4046-2995 cal BP (95.4%)* or *3681-3333 cal BP (68.2%).* The single-phase model contains four radiocarbon determinations obtained from unidentified charcoal. Such a small number of determinations results in an estimate with poor precision. This assemblage comprises red-slip pottery with a small number of punctate sherds [12].

Plot()

{

Outlier_Model("Charcoal", Exp(1,-10,0), U(0,3), "t");

Sequence("Luzon_Irigayen")

{

Boundary("start");

Phase("Irigayen")

{

R_Date("NUTA2-917",3185,25)

{

Outlier(1);

};

R_Date("NUTA2-913",3165,25)

{

Outlier(1);

};

R_Date("NUTA2-914",3025,20)

{

Outlier(1);

};

R_Date("NUTA2-912",2925,30)

{

Outlier(1);

};

};

Boundary("end");

};

};

**Nagsabaran Site, Luzon Island**

Five models were created for the Nagsabaran site on Luzon using the accepted radiocarbon determinations from the lower silt layers. A single-phase model was created for Test Pit (TP) 2. A two-phase model with sequential ordering was created for adjoining excavation units TP 7 and TP 9 with the phases based on published sample depths. A single-phase model was created for TP 14 using the two reported determinations from 240 cm depth. The determinations used in the TP 2, TP 7/TP 9, and TP 14 models were combined with individual determinations from TP 4 (GX-26711-A, 2520±20 BP), TP 11 (ANU-13016, 3510±30 BP), and an additional date reported by Carson and Hung [15] (Beta-436818, 3760±30 BP, provenience unreported) in a single-phase model for the site. Another iteration of the single-phase, site-scale model included a single freshwater shell-derived determination from TP 1 (NTU-3799, 3450±40 BP). Early Nagsabaran ceramics comprise red-slip, impressed, circle-stamp, and dentate surface treatments [18, 27]

TP 2 Nagsabaran

The TP 2 model estimates the appearance of pottery sometime between *16283-7323 cal BP (95.4%)* or *16283-7277 cal BP (68.2%*). The single-phase model contains two radiocarbon determinations obtained from unidentified charcoal. GX-26705 (6610±290 BP) is anomalously old and is driving the results. Therefore, it was excluded from the site- and island-scale models. Such a small number of determinations results in an estimate with poor precision.

Plot()

{

Outlier_Model("Charcoal", Exp(1,-10,0), U(0,3), "t");

Sequence("Luzon_Nagsabaran_TP2")

{

Boundary("start");

Phase("Nagsabaran TP2")

{

R_Date("GX-26705",6610,290)

{

Outlier(1);

};

R_Date("GX-26704-A",2620,40)

{

Outlier(1);

};

};

Boundary("end");

};

};

TP 7/TP 9 Nagsabaran

The TP 7/TP 9 model estimates the appearance of pottery sometime between *4054-1551 cal BP (95.4%)* or *2917-1864 cal BP (68.2%).* The two-phase model contains six radiocarbon determinations obtained from unidentified charcoal.

Plot()

{

Outlier_Model("Charcoal", Exp(1,-10,0), U(0,3), "t");

Sequence("Luzon_Nagsabaran_TP7-TP9")

{

Boundary("start");

Phase("190-180 cm")

{

R_Date("GX-28381",3390,130)

{

Outlier(1);

};

R_Date("Wk-17756",2528,31)

{

Outlier(1);

};

};

Boundary("end 190-180 cm");

Boundary("start 160-150 cm");

Phase("160-150 cm")

{

R_Date("GX-28379",3050,70)

{

Outlier(1);

};

R_Date("NTU-3798",2670,40)

{

Outlier(1);

};

R_Date("Wk-18059",1946,30)

{

Outlier(1);

};

R_Date("WK-19713",4450,39)

{

Outlier(1);

};

};

Boundary("end");

};

};

TP 14 Nagsabaran

The TP 14 estimate for this event is *3593-2605 cal BP (95.4%)* or *3587-2671 cal BP (68.2%).* The single-phase model contains two radiocarbon determinations obtained from unidentified charcoal. Such a small number of determinations results in an estimate with poor precision.

Plot()

{

Outlier_Model("Charcoal", Exp(1,-10,0), U(0,3), "t");

Sequence("Luzon_Nagsabaran_TP14")

{

Boundary("start");

Phase("Nagsabaran TP14")

{

R_Date("ANU-13014",2660,30)

{

Outlier(1);

};

R_Date("ANU-13013",2540,30)

{

Outlier(1);

};

};

Boundary("end");

};

};

Nagsabaran Site without shell determinations

A combined Nagsabaran model was created with 12 radiocarbon determinations from the individual excavation areas (excluding GX-26705). The estimate for the beginning of ceramic deposition is sometime no earlier than *5874-2970 cal BP (95.4%)* or *5680-2976 cal BP (68.2%).* The larger sample size of the site-scale model presumably results in accuracy, but imprecision is introduced by the substantial discrepancy between the oldest (Wk-19713, 4450±39 BP) and youngest (Wk-18059, 1946±30 BP) determinations. This is our preferred model for Nagsabaran.

Plot()

{

Outlier_Model("Charcoal", Exp(1,-10,0), U(0,3), "t");

Sequence("Luzon_Nagsabaran")

{

Boundary("start");

Phase("Nagsabaran")

{

R_Date("Wk-19713",4450,39)

{

Outlier(1);

};

R_Date("Beta-436818",3760,30)

{

Outlier(1);

};

R_Date("ANU-13016",3510,30)

{

Outlier(1);

};

R_Date("GX-28381",3390,130)

{

Outlier(1);

};

R_Date("GX-28379",3050,70)

{

Outlier(1);

};

R_Date("NTU-3798",2670,40)

{

Outlier(1);

};

R_Date("ANU-13014",2660,30)

{

Outlier(1);

};

R_Date("GX-26704-A",2620,40)

{

Outlier(1);

};

R_Date("ANU-13013",2540,30)

{

Outlier(1);

};

R_Date("Wk-17756",2528,31)

{

Outlier(1);

};

R_Date("GX-26711-A",2520,50)

{

Outlier(1);

};

R_Date("Wk-18059",1946,30)

{

Outlier(1);

};

};

Boundary("end");

};

};

Nagsabaran Site with shell determination

The Nagsabaran site model was modified with the addition of one freshwater shell-derived determination (*Batissa childreni*) from TP 1, which was calibrated using IntCal13. The shell-inclusive model provides an estimate for the onset of ceramic deposition sometime between *6079-3717 cal BP (95.4%)* or *5762-3999 cal BP (68.2%).* The non-shell and shell model results differ by about a century. Because there remains uncertainty as to whether a localized correction factor is required for freshwater shellfish from this area, we prefer the non-shell site-scale model for Nagsabaran.

Plot()

{

Outlier_Model("General",T(5),U(0,4),"t");

Outlier_Model("Charcoal", Exp(1,-10,0), U(0,3), "t");

Sequence("Luzon_Nagsabaran")

{

Boundary("start");

Phase("Nagsabaran")

{

R_Date("Wk-19713",4450,39)

{

Outlier("Charcoal",1);

};

R_Date("Beta-436818",3760,30)

{

Outlier("Charcoal",1);

};

R_Date("ANU-13016",3510,30)

{

Outlier("Charcoal",1);

};

R_Date("NTU-3799",3450,40)

{

Outlier("General",0.05);

};

R_Date("GX-28381",3390,130)

{

Outlier("Charcoal",1);

};

R_Date("GX-28379",3050,70)

{

Outlier("Charcoal",1);

};

R_Date("NTU-3798",2670,40)

{

Outlier("Charcoal",1);

};

R_Date("ANU-13014",2660,30)

{

Outlier("Charcoal",1);

};

R_Date("GX-26704-A",2620,40)

{

Outlier("Charcoal",1);

};

R_Date("ANU-13013",2540,30)

{

Outlier("Charcoal",1);

};

R_Date("Wk-17756",2528,31)

{

Outlier("Charcoal",1);

};

R_Date("GX-26711-A",2520,50)

{

Outlier("Charcoal",1);

};

R_Date("Wk-18059",1946,30)

{

Outlier(1);

};

};

Boundary("end");

};

};

**Luzon Island**

The determinations from the Andarayan, Irigayen, and Nagsabaran models were combined with individual ages from Callao and Dalan Serkot in two single-phase island-wide models for Luzon: one model included the Nagsabaran determination derived from *B. childreni* (NTU-3799, 3450±40 BP) for a total of 21 determinations in the model; the other model excluded that determination for a total of 20 determinations. As stated for the combined Nagsabaran model, GX-26705 was manually identified as an outlier and was excluded from both of the Luzon models. At this scale, the appearance of pottery is estimated to have occurred no earlier than *5447-3650 cal BP (95.4%)* or *5163-3688 cal BP (68.2%)* (shell-inclusive model) or no earlier than *5322-3410 cal BP (95.4%)* or *4151-3557 cal BP (68.2%)* (non-shell model). Because of the uncertainty regarding the need for a localized correction factor for freshwater shells at Nagsabaran, we prefer the non-shell model.

Luzon Island with shell determination

Plot()

{

Outlier_Model("General",T(5),U(0,4),"t");

Outlier_Model("Charcoal", Exp(1,-10,0), U(0,3), "t");

Sequence("Luzon")

{

Boundary("start");

Phase("Luzon")

{

R_Date("Wk-19713",4450,39)

{

Outlier("Charcoal",1);

};

R_Date("Beta-436818",3760,30)

{

Outlier("Charcoal",1);

};

R_Date("Wk-15648",3530,34)

{

Outlier("Charcoal",1);

};

R_Date("ANU-13016",3510,30)

{

Outlier("Charcoal",1);

};

R_Date("NTU-3799",3450,40)

{

Outlier("General",0.05);

};

R_Date("Unreported",3400,125)

{

Outlier("General",0.05);

};

R_Date("GX-28381",3390,130)

{

Outlier("Charcoal",1);

};

R_Date("Wk-17010",3335,34)

{

Outlier("Charcoal",1);

};

R_Date("SFU-86",3240,160)

{

Outlier("Charcoal",1);

};

R_Date("NUTA2-917",3185,25)

{

Outlier("Charcoal",1);

};

R_Date("NUTA2-913",3165,25)

{

Outlier("Charcoal",1);

};

R_Date("GX-28379",3050,70)

{

Outlier("Charcoal",1);

};

R_Date("NUTA2-914",3025,20)

{

Outlier("Charcoal",1);

};

R_Date("NUTA2-912",2925,30)

{

Outlier("Charcoal",1);

};

R_Date("NTU-3798",2670,40)

{

Outlier("Charcoal",1);

};

R_Date("ANU-13014",2660,30)

{

Outlier("Charcoal",1);

};

R_Date("GX-26704-A",2620,40)

{

Outlier("Charcoal",1);

};

R_Date("ANU-13013",2540,30)

{

Outlier("Charcoal",1);

};

R_Date("Wk-17756",2528,31)

{

Outlier("Charcoal",1);

};

R_Date("GX-26711-A",2520,50)

{

Outlier("Charcoal",1);

};

R_Date("Wk-18059",1946,30)

{

Outlier("Charcoal",1);

};

};

Boundary("end");

};

};

Luzon Island without shell determination

Plot()

{

Outlier_Model("General",T(5),U(0,4),"t");

Outlier_Model("Charcoal", Exp(1,-10,0), U(0,3), "t");

Sequence("Luzon")

{

Boundary("start");

Phase("Luzon")

{

R_Date("Wk-19713",4450,39)

{

Outlier("Charcoal",1);

};

R_Date("Beta-436818",3760,30)

{

Outlier("Charcoal",1);

};

R_Date("Wk-15648",3530,34)

{

Outlier("Charcoal",1);

};

R_Date("ANU-13016",3510,30)

{

Outlier("Charcoal",1);

};

R_Date("Unreported",3400,125)

{

Outlier("General",0.05);

};

R_Date("GX-28381",3390,130)

{

Outlier("Charcoal",1);

};

R_Date("Wk-17010",3335,34)

{

Outlier("Charcoal",1);

};

R_Date("SFU-86",3240,160)

{

Outlier("Charcoal",1);

};

R_Date("NUTA2-917",3185,25)

{

Outlier("Charcoal",1);

};

R_Date("NUTA2-913",3165,25)

{

Outlier("Charcoal",1);

};

R_Date("GX-28379",3050,70)

{

Outlier("Charcoal",1);

};

R_Date("NUTA2-914",3025,20)

{

Outlier("Charcoal",1);

};

R_Date("NUTA2-912",2925,30)

{

Outlier("Charcoal",1);

};

R_Date("NTU-3798",2670,40)

{

Outlier("Charcoal",1);

};

R_Date("ANU-13014",2660,30)

{

Outlier("Charcoal",1);

};

R_Date("GX-26704-A",2620,40)

{

Outlier("Charcoal",1);

};

R_Date("ANU-13013",2540,30)

{

Outlier("Charcoal",1);

};

R_Date("Wk-17756",2528,31)

{

Outlier("Charcoal",1);

};

R_Date("GX-26711-A",2520,50)

{

Outlier("Charcoal",1);

};

R_Date("Wk-18059",1946,30)

{

Outlier("Charcoal",1);

};

};

Boundary("end");

};

};

**Greater Sunda Islands**

Twelve single- or multi-phase models were created for site deposits and islands within the Greater Sunda Islands: Borneo[28-31], Java [32], and Sulawesi [33-37]. For those sites that produced radiocarbon determinations from stratified deposits with more than one determination per layer, multi-phase models were created. Determinations from these sites and four other site deposits or excavation areas that produced individual radiocarbon ages (Braholo Cave [32], Keplek Cave [32], Mansiri [38], and Minango Sipakko, Test Pit 2 [37]) were included in single-phase models for each respective island. Two determinations from Topogaro 2 Cave in eastern Sulawesi [39] were not used as it is unclear which determination, or if both, are most clearly associated with the ceramics. A chronology of Kamassi site deposits on Sulawesi must rely on four radiocarbon determinations, all from freshwater (*Melanoides* sp.) and estuarine shells (*Geloina* sp.). We produced two models of the Kamassi site following procedures in Materials and Methods for marine calibration, but there is no systematic way to choose one model over another. Thus the Kamassi site results were not used in our analysis, but the radiocarbon data is included in S3 Table. The exclusion of the Kamassi site does not alter our overall conclusions.

In consideration of the large size of Borneo and the likelihood that the initial appearance of ceramics varied spatially through time, a single-phase model was also run for the northern portion of the island (other areas of the island are only represented by individual sites) (S3 Fig).

Dating samples were unidentified wood charcoal, freshwater shell, human bone, and a rice husk. For the one site that has a determination from freshwater shell, models were created with and without this age. Lacking reservoir correction values, this was done to evaluate the effect that the shell-derived determinations have on date estimates. The age obtained from the human bone specimen at Niah Cave (unreported laboratory number, 4990±90 BP) [40] is excluded since isotopic data is not reported and therefore it is unknown whether a mixed atmospheric and marine calibration is warranted. A single determination from Jambu Hilir (Wk-22009, 2922±45 BP) was not used in the models as pottery appears in contexts up to 90 cm below the dated sample [41]. It is not clear, therefore, that the dated sample is associated with earliest ceramic deposition.

**Liang Abu Site, Borneo**

The initial appearance of ceramics at the Liang Abu site on Borneo Island is estimated to have occurred sometime between *2178-1386 cal BP (95.4%) or 2178-1477 cal BP (68.2%)*. The single-phase model contains two radiocarbon determinations obtained from unidentified charcoal. Such a small number of determinations results in an estimate with poor precision.

Plot()

{

Outlier_Model("Charcoal", Exp(1,-10,0), U(0,3), "t");

Sequence("Borneo_Liang Abu")

{

Boundary("start");

Phase("Liang Abu")

{

R_Date("UBA-20839",1672,21)

{

Outlier(1);

};

R_Date("UBA-20840",1524,22)

{

Outlier(1);

};

};

Boundary("end");

};

};

**MAD1 Site, Borneo**

Two multi-phase models were created for the MAD1 site. One contains five radiocarbon determinations obtained from unidentified charcoal, which are organized in a contiguous sequence based on excavation level. The oldest determinations are from a pre-ceramic level (Level 12), with Level 11a being the deepest ceramic deposit, which is capped by another ceramic-bearing deposit (Layer 10). The boundary between the phases for Levels 12 and 11a (“transition Layer 12/Layer 11a”) is the event of interest. The other model has the same structure but includes a single freshwater shell determination (ANU-2943, 2700±70 BP) in the post-initial ceramic phase.

The first model estimates that the initial appearance of ceramics at the MAD1 site occurred sometime between *8493-2662 cal BP (95.4%) or 8458-2709 cal BP (68.2%)*. The second model estimates that this event occurred sometime between *8531-2730 cal BP (95.4%) or 8447-2748 cal BP (68.2%)*. The difference between these estimates is minor. However, due to the uncertainty introduced by the freshwater shell determination, our preferred iteration is the charcoal-only model.

MAD1 Site without shell determination

Plot()

{

Outlier_Model("Charcoal", Exp(1,-10,0), U(0,3), "t");

Sequence("Sabah MAD1")

{

Boundary("start");

Phase("Layer 12, preceramic")

{

R_Date("ANU-2397",7920,370)

{

Outlier(1);

};

R_Date("ANU-2398",7390,270)

{

Outlier(1);

};

};

Boundary("transition Layer 12/Layer 11a");

Phase("Layer 11a")

{

R_Date("ANU-2396",2650,80)

{

Outlier(1);

};

};

Boundary("transition Layer 11a/Layer 10");

Phase("Layer 10")

{

R_Date("ANU-2945",2020,90)

{

Outlier(1);

};

R_Date("ANU-2395",1590,150)

{

Outlier(1);

};

};

Boundary("end");

};

};

MAD1 Site with shell determination

Plot()

{

Outlier_Model("General",T(5),U(0,4),"t");

Outlier_Model("Charcoal", Exp(1,-10,0), U(0,3), "t");

Sequence("Sabah MAD1")

{

Boundary("start");

Phase("Layer 12, preceramic")

{

R_Date("ANU-2397",7920,370)

{

Outlier("Charcoal",1);

};

R_Date("ANU-2398",7390,270)

{

Outlier("Charcoal",1);

};

};

Boundary("transition Layer 12/Layer 11a");

Phase("Layer 11a")

{

R_Date("ANU-2396",2650,80)

{

Outlier("Charcoal",1);

};

};

Boundary("transition Layer 11a/Layer 10");

Phase("Layer 10")

{

R_Date("ANU-2945",2020,90)

{

Outlier("Charcoal",1);

};

R_Date("ANU-2395",1590,150)

{

Outlier("Charcoal",1);

};

R_Date("ANU-2943",2700,70)

{

Outlier("General",0.05);

};

};

Boundary("end");

};

};

**Gua Sireh Site, Borneo**

The initial appearance of ceramics at the Gua Sireh site on Borneo is estimated to have occurred sometime between *9663-3873 cal BP (95.4%) or 5542-4072 cal BP (68.2%)*. The single-phase model contains two radiocarbon determinations obtained from unidentified charcoal and rice husk embedded in a ceramic sherd. Such a small number of determinations results in an estimate with poor precision.

Plot()

{

Outlier_Model("General",T(5),U(0,4),"t");

Outlier_Model("Charcoal", Exp(1,-10,0), U(0,3), "t");

Sequence("Gua Sireh")

{

Boundary("start");

Phase("Gua Sireh")

{

R_Date("CA-725",3850,260)

{

Outlier("General",0.05);

};

R_Date("ANU-7049",3990,230)

{

Outlier("Charcoal",1);

};

};

Boundary("end");

};

};

**Bukit Tengkorak Shelter Site, Borneo**

The initial appearance of ceramics at the Bukit Tengkorak Shelter site on Borneo Island is estimated to have occurred sometime between *5798-2347 cal BP (95.4%) or 5795-2429 cal BP (68.2%)*. The single-phase model contains two radiocarbon determinations obtained from unidentified charcoal. Such a small number of determinations results in an estimate with poor precision. The radiocarbon determinations from Trench G17 fronting the shelter were not modeled as the basal spit containing ceramics contained no determination. Only one determination was obtained from the penultimate spit containing ceramics.

Plot()

{

Outlier_Model("Charcoal", Exp(1,-10,0), U(0,3), "t");

Sequence("Bukit Tengkorak Shelter")

{

Boundary("start");

Phase("Bukit Tengkorak Shelter")

{

R_Date("ANU-5769",2700,110)

{

Outlier(1);

};

R_Date("ANU-5770",2330,170)

{

Outlier(1);

};

};

Boundary("end");

};

};

**Borneo Island**

The charcoal and rice husk determinations from the model phases of the basal ceramic-bearing deposits at Liang Abu, Bukit Tengkorak, MAD1, and Gua Sireh were combined in a single-phase island-wide model for Borneo. The freshwater shell determination was excluded since they did not significantly affect the site-scale models but also still introduce a degree of uncertainty regarding the need for localized correction factors. At this geographic scale, the appearance of pottery is estimated to have occurred sometime between *6173-3749 cal BP (95.4%) or 5117-4129 cal BP (68.2%)*. These date estimates are associated with red-slip pottery, along with cord-mark, incised, impressed, and circle-stamp surface treatments at some sites [28-31]

Plot()

{

Outlier_Model("General",T(5),U(0,4),"t");

Outlier_Model("Charcoal", Exp(1,-10,0), U(0,3), "t");

Sequence("Borneo_Island")

{

Boundary("start");

Phase("Borneo")

{

R_Date("ANU-7049",3990,230)

{

Outlier("Charcoal"1);

};

R_Date("CA-725",3850,260)

{

Outlier("General",0.05);

};

R_Date("ANU-5769",2700,110)

{

Outlier("Charcoal"1);

};

R_Date("ANU-2396",2650,80)

{

Outlier("Charcoal"1);

};

R_Date("ANU-5770",2330,170)

{

Outlier("Charcoal"1);

};

R_Date("UBA-20839",1672,21)

{

Outlier("Charcoal"1);

};

R_Date("UBA-20840",1524,22)

{

Outlier("Charcoal"1);

};

};

Boundary("end");

};

};

**Northern Borneo**

Since there may have been spatial variation in the timing of the adoption of pottery across Borneo, a single-phase model was created for northern Borneo that consists of the determinations from Liang Abu, Bukit Tengkorak, and MAD1. For this portion of the island, the appearance of pottery is estimated to have occurred sometime between *4580-1862 cal BP (95.4%) or 3460-2665 cal BP (68.2%)*.

Plot()

{

Outlier_Model("Charcoal", Exp(1,-10,0), U(0,3), "t");

Sequence("Borneo_Island")

{

Boundary("start");

Phase("North Borneo")

{

R_Date("ANU-5769",2700,110)

{

Outlier(1);

};

R_Date("ANU-2396",2650,80)

{

Outlier(1);

};

R_Date("ANU-5770",2330,170)

{

Outlier(1);

};

R_Date("UBA-20839",1672,21)

{

Outlier(1);

};

R_Date("UBA-20840",1524,22)

{

Outlier(1);

};

};

Boundary("end");

};

};

**Malawa Site, Sulawesi Island**

The initial appearance of ceramics at the Malawa site on Sulawesi Island is estimated to have occurred sometime between *7928-3077 cal BP (95.4%) or 7928-3516 cal BP (68.2%)*. The single-phase model contains two radiocarbon determinations obtained from unidentified charcoal. Such a small number of determinations results in an estimate with poor precision.

Plot()

{

Outlier_Model("Charcoal", Exp(1,-10,0), U(0,3), "t");

Sequence("Sulawesi Malawa")

{

Boundary("start");

Phase("Malawa")

{

R_Date("P3G-06",3580,130)

{

Outlier(1);

};

R_Date("P3G-06",2710,170)

{

Outlier(1);

};

};

Boundary("end");

};

};

**Minango Sipakko Site, Sulawesi Island**

Two models provided results for the Minango Sipakko site on Sulawesi Island. A single-phase model was created for Layer 2, the basal ceramic deposit sampled by Units M3, M4, and M5 (these excavation units were contiguous forming a larger trench excavation). Another single-phase model was created using determinations from Test Pit 2, Unit M1, and Units M3 to M5. Additionally, a multi-phase model was created for Layer 2 based on excavation depths within the approximately 1-m-thick stratum; however, this model failed due to inversions in the ages. All determinations are from unidentified charcoal.

Based on the single-phase model for Layer 2, the initial occurrence of ceramics at the site occurred sometime between *7659-… cal BP (95.4%) or 6459-2789 cal BP (68.2%)*; at 95.4%, the model failed to converge on a younger date as represented by “…” in the calibrated range. The single-phase model for the site as a whole produces an estimate for the onset of ceramics sometime between *6824-2732 cal BP (95.4%) or 6052-2813 cal BP (68.2%)*. The oldest determination from Unit M5 (which also has a large error range) is strongly influencing the older limits of the estimate. We prefer the site-scale model since it has the largest number of determinations.

Minango Sipakko Site, Layer 2, Units M3, M4, and M5

Plot()

{

Outlier_Model("Charcoal", Exp(1,-10,0), U(0,3), "t");

Sequence("Minango Sipakko")

{

Boundary("start");

Phase("Layer 2")

{

R_Date("P3G-05",4950,180)

{

Outlier(1);

};

R_Date("P3G-05",3690,160)

{

Outlier(1);

};

R_Date("Wk-14651",3446,54)

{

Outlier(1);

};

R_Date("Wk-14652",3082,50)

{

Outlier(1);

};

R_Date("Wk-14654",2996,41)

{

Outlier(1);

};

R_Date("Wk-14653",2881,46)

{

Outlier(1);

};

};

Boundary("end");

};

};

Minango Sipakko Site

Plot()

{

Outlier_Model("Charcoal", Exp(1,-10,0), U(0,3), "t");

Sequence("Sulawesi Minanga Sipakko")

{

Boundary("start");

Phase("Minanga Sipakko")

{

R_Date("P3G-05",4950,180)

{

Outlier(1);

};

R_Date("P3G-05",3690,160)

{

Outlier(1);

};

R_Date("Wk-14651",3446,54)

{

Outlier(1);

};

R_Date("Wk-17981",3343,46)

{

Outlier(1);

};

R_Date("Wk-14652",3082,50)

{

Outlier(1);

};

R_Date("Wk-14654",2996,41)

{

Outlier(1);

};

R_Date("Wk-14653",2881,46)

{

Outlier(1);

};

};

Boundary("end");

};

};

**Sulawesi Island**

The determinations from the Malawa and Minango Sipakko models were combined with an individual age from Mansiri in a single-phase, island-wide model for Sulawesi. At this geographic scale, the appearance of pottery is estimated to have occurred sometime between *6358-2428 cal BP (95.4%) or 5813-2552 cal BP (68.2%)*. The early Sulawesi ceramic deposits contain red-slip, impressed, incised, or circle-stamped sherds [33, 35, 37, 42]

Plot()

{

Outlier_Model("Charcoal", Exp(1,-10,0), U(0,3), "t");

Sequence("Sulawesi Island")

{

Boundary("start");

Phase("Sulawesi")

{

R_Date("P3G-05",4950,180)

{

Outlier(1);

};

R_Date("P3G-05",3690,160)

{

Outlier(1);

};

R_Date("P3G-06",3580,130)

{

Outlier(1);

};

R_Date("Wk-14651",3446,54)

{

Outlier(1);

};

R_Date("Wk-17981",3343,46)

{

Outlier(1);

};

R_Date("Wk-14652",3082,50)

{

Outlier(1);

};

R_Date("Wk-14654",2996,41)

{

Outlier(1);

};

R_Date("Wk-14653",2881,46)

{

Outlier(1);

};

R_Date("P3G-06",2710,170)

{

Outlier(1);

};

R_Date("Wk-44610",2469,20)

{

Outlier(1);

};

};

Boundary("end");

};

};

**Java Island**

Single radiocarbon determinations from Braholo and Keplek Caves [32] were combined in a single-phase island-wide model for Java. Both determinations are from unidentified charcoal. At this geographic scale, the appearance of pottery is estimated to have occurred sometime between *5893-3107 cal BP (95.4%) or 5888-3177 cal BP (68.2%)*. These date estimates are associated with pottery described as earthenware [32].

Plot()

{

Outlier_Model("Charcoal", Exp(1,-10,0), U(0,3), "t");

Sequence("Java")

{

Boundary("start");

Phase("Java")

{

R_Date("P3G-1998",3260,110)

{

Outlier(1);

};

R_Date("P3G-1999",3050,100)

{

Outlier(1);

};

};

Boundary("end");

};

};

**Lesser Sunda Islands**

Five single-phase models were created for site deposits in the Lesser Sunda Islands (S4 Fig). This includes Timor [43] and Flores Island [44]. Dating samples were unidentified charcoal, marine shell, and human bone. Marine shell determinations were calibrated using Marine13 with either a ΔR of 0±0 or a uniform ΔR calculated from the range of values for the region (-57 to 92). Human bone determinations were calibrated using either IntCal13 or a mixed marine-atmospheric curve.

**Lena Hara Site, Timor**

Two models were created for Lena Hara Cave, Timor; these are also the island-scale models. Two determinations from Square F, spit 16 (61-68 cm bs) were included in a single-phase model. One determination is from unidentified charcoal adhering to the exterior surface of a sherd and the other determination is from a marine gastropod (*Trochus niloticus*). Both models use Marine13 with a ΔR of 0±0 applied to one model and a uniform ΔR calculated from the range of values for the region (-57 to 92) applied to the other.

With the first model, the initial appearance of pottery is estimated to have occurred sometime between *8668-3603 cal BP (95.4%) or 4921-3653 cal BP (68.2%)*. With the second model, the initial appearance of pottery is estimated to have occurred sometime between *8665-3524 cal BP (95.4%) or 4495-3620 cal BP (68.2%)*. Differences between the results are negligible. However, because a localized correction value remains uncertain, we prefer the first model acknowledging that it may be inaccurate. Such a small number of determinations results in an estimate with poor precision. The age estimates are associated with red-slipped and plain pottery [43].

Lena Hara Site, Timor, 0 ΔR

Plot()

{

Outlier_Model("General",T(5),U(0,4),"t");

Outlier_Model("Charcoal", Exp(1,-10,0), U(0,3), "t");

Sequence("Lena Hara Cave")

{

Boundary("start");

Phase("Square F, Spit 16")

{

R_Date("ANU-12029",3200,240)

{

Outlier("Charcoal",1);

};

Curve("Marine13","Marine13.14c");

Delta_R("LocalMarine", 0,0);

R_Date("ANU-12030",3850,70)

{

Outlier("General",0.05);

};

};

Boundary("end");

};

};

Lena Hara, Timor, uniform ΔR

Plot()

{

Outlier_Model("General",T(5),U(0,4),"t");

Outlier_Model("Charcoal", Exp(1,-10,0), U(0,3), "t");

Sequence("Lena Hara Cave")

{

Boundary("start");

Phase("Square F, Spit 16")

{

R_Date("ANU-12029",3200,240)

{

Outlier("Charcoal",1);

};

Curve("Marine13","Marine13.14c");

Delta_R("Uniform", U(-57,92));

R_Date("ANU-12030",3850,70)

{

Outlier("General",0.05);

};

};

Boundary("end");

};

};

**Pain Haka Site, Flores**

Three single-phase models were created for Pain Haka, Flores Island; these are also the island-scale models. Dating samples were unidentified charcoal and human bone from burials associated with ceramic vessels. The contexts for the charcoal, with one exception (Wk-36712), are unreported, but presumably originate from the archaeological deposit(s) associated with the burials. Galipaud et al. ([44, supplementary material]) note that the nitrogen and carbon stable isotope ratios of the human bone samples possibly indicate the consumption of C_4_ plants or low trophic level marine foods. Because of this ambiguity in past diet, they calibrate the bone-derived determinations with the northern hemisphere atmospheric curve. Following Galipaud et al., we created one model that uses the atmospheric curve for the human bone and charcoal determinations. We ran two other models that assign a mixed marine-atmospheric curve (25:75) with different ΔR values (0±0 and a uniform ΔR calculated from the range of values for the region [-57 to 92]) to evaluate the effect of a marine diet.

The initial appearance of ceramics at the Pain Haka site on Flores Island is estimated to have occurred sometime between *3147-2717 cal BP (95.4%) or 3006-2724 cal BP (68.2%)* based on the model that applies the atmospheric curve. The timing of this event is estimated as *3095-2785 cal BP (95.4%) or 2945-2820 cal BP (68.2%)* based on the model that applies the mixed marine-atmospheric curve and a ΔR of 0±0. Using the uniform ΔR command, this event is estimated as *3166-2781 cal BP (95.4%) or 2963-2824 cal BP (68.2%)*. Considering the site’s shoreline proximity, modeling with a mixed marine-atmospheric curve is our preference, and until a localized ΔR value is calculated we prefer the model with a ΔR of 0±0. The Pain Haka ceramics include red-slip, incised, and applique surface treatments [44].

Pain Haka Site, Flores, atmospheric curve

Plot()

{

Outlier_Model("General",T(5),U(0,4),"t");

Outlier_Model("Charcoal", Exp(1,-10,0), U(0,3), "t");

Sequence("Pain Haka")

{

Boundary("start");

Phase("Pain Haka")

{

R_Date("Wk-36712",2784,25)

{

Outlier("Charcoal",1);

};

R_Date("Wk-28997",2725,25)

{

Outlier("Charcoal",1);

};

R_Date("Wk-28996",2535,25)

{

Outlier("Charcoal",1);

};

R_Date("Wk-28995",2509,25)

{

Outlier("Charcoal",1);

};

R_Date("Wk-36556",2831,25)

{

Outlier("General",0.05);

};

R_Date("Wk-36558",2588,25)

{

Outlier("General",0.05);

};

R_Date("Wk-36557",2570,25)

{

Outlier("General",0.05);

};

R_Date("Wk-36559",2548,25)

{

Outlier("General",0.05);

};

R_Date("Wk-41599",2532,20)

{

Outlier("General",0.05);

};

R_Date("Wk-36560",2246,25)

{

Outlier("General",0.05);

};

};

Boundary("end");

};

};

Pain Haka Site, Flores, mixed curve, 0 ΔR

Plot()

{

Outlier_Model("General",T(5),U(0,4),"t");

Outlier_Model("Charcoal", Exp(1,-10,0), U(0,3), "t");

Sequence("Pain Haka")

{

Boundary("start");

Phase("Pain Haka")

{

R_Date("Wk-36712",2784,25)

{

Outlier("Charcoal",1);

};

R_Date("Wk-28997",2725,25)

{

Outlier("Charcoal",1);

};

R_Date("Wk-28996",2535,25)

{

Outlier("Charcoal",1);

};

R_Date("Wk-28995",2509,25)

{

Outlier("Charcoal",1);

};

Curve("IntCal13","IntCal13.14c");

Curve("Marine13","Marine13.14c");

Delta_R("LocalMarine", 0,0);

Mix_Curve("Mixed","IntCal13","Marine13",25,0);

R_Date("Wk-36556",2831,25)

{

Outlier("General",0.05);

};

R_Date("Wk-36558",2588,25)

{

Outlier("General",0.05);

};

R_Date("Wk-36557",2570,25)

{

Outlier("General",0.05);

};

R_Date("Wk-36559",2548,25)

{

Outlier("General",0.05);

};

R_Date("Wk-41599",2532,20)

{

Outlier("General",0.05);

};

R_Date("Wk-36560",2246,25)

{

Outlier("General",0.05);

};

};

Boundary("end");

};

};

Pain Haka Site, Flores, mixed curve, uniform ΔR

Plot()

{

Outlier_Model("General",T(5),U(0,4),"t");

Outlier_Model("Charcoal", Exp(1,-10,0), U(0,3), "t");

Sequence("Pain Haka")

{

Boundary("start");

Phase("Pain Haka")

{

R_Date("Wk-36712",2784,25)

{

Outlier("Charcoal",1);

};

R_Date("Wk-28997",2725,25)

{

Outlier("Charcoal",1);

};

R_Date("Wk-28996",2535,25)

{

Outlier("Charcoal",1);

};

R_Date("Wk-28995",2509,25)

{

Outlier("Charcoal",1);

};

Curve("IntCal13","IntCal13.14c");

Curve("Marine13","Marine13.14c");

Delta_R("Uniform", U(-57,92));

Mix_Curve("Mixed","IntCal13","Marine13",25,0);

R_Date("Wk-36556",2831,25)

{

Outlier("General",0.05);

};

R_Date("Wk-36558",2588,25)

{

Outlier("General",0.05);

};

R_Date("Wk-36557",2570,25)

{

Outlier("General",0.05);

};

R_Date("Wk-36559",2548,25)

{

Outlier("General",0.05);

};

R_Date("Wk-41599",2532,20)

{

Outlier("General",0.05);

};

R_Date("Wk-36560",2246,25)

{

Outlier("General",0.05);

};

};

Boundary("end");

};

};

**Molucca Islands**

A single multi-phase model was created for site deposits at PA1, Pulau Ay [45] (S5 Fig). The site model is also the island-scale model. Um Kapat Papo on Gebe Island and Uattamdi on Kayoa Island [46] were excluded from analysis as these sites contain only shell-derived radiocarbon determinations and, like Kamassi in the Greater Sundas, it is not possible to systematically choose the most valid model. Multiple models applying different calibration parameters were generated for each site as exploratory analyses. While the Uattamdi results do not alter our overall conclusions, the large HPD for Um Kapat Papo does raise the possibility for early ceramic production on Gebe Island; this must be addressed by a reliable suite of radiocarbon determinations. Tanjung Pinang on Morotai and Siti Nafisah on Halmahera are excluded from analysis as it is unclear which radiocarbon determinations relate to the pottery-bearing portions of the archaeological deposits [46:237-242, 253-254]. Two determinations from the LTM site in the Talaud Islands [47] are also excluded from modeling because they are from different excavation units and are not stratigraphically correlated. All of these determinations, while not used in our analyses, appear in S3 Table.

Dating samples were unidentified charcoal, unidentified estuarine shell, and unidentified marine shell. Marine shell determinations were calibrated using Marine13 with a ΔR of 0±0 applied to one model and a uniform ΔR calculated from the range of values for the region (-57 to 92) applied to a second model iteration. Estuarine shell determinations were calibrated with IntCal13.

**PA1, Pulau Ay**

The initial appearance of ceramics at the PA1 site on Pulau Ay is estimated to have occurred sometime between *3967-2775 cal BP (95.4%) or 3551-3151 cal BP (68.2%)*. The two-phase model contains seven radiocarbon determinations obtained from unidentified charcoal. Two radiocarbon determinations are from the basal ceramic-bearing deposit (Layer 6) with five determinations from two overlying strata (Layers 3 and 4), which are separated from the basal deposit by a culturally sterile pumice layer. The assemblage contains red-slip and incised sherds, with circle-stamp appearing in more recent deposits [45].

Plot()

{

Outlier_Model("Charcoal", Exp(1,-10,0), U(0,3), "t");

Sequence("Pulau Ay PA1")

{

Boundary("start");

Phase("Layer 6")

{

R_Date("240739",3190,40)

{

Outlier(1);

};

R_Date("Beta-235453",3010,40)

{

Outlier(1);

};

};

Boundary("end Layer 6");

Boundary("start post-Layer 6 strata");

Phase("Layers 3 & 4")

{

R_Date("Beta-240738",3010,40)

{

Outlier(1);

};

R_Date("Beta-304478",2940,40)

{

Outlier(1);

};

R_Date("Beta-302405",2880,30)

{

Outlier(1);

};

R_Date("Beta-302404",2780,30)

{

Outlier(1);

};

R_Date("Beta-235454",2460,40)

{

Outlier(1);

};

};

Boundary("end post-Layer 6 strata");

};

};

**Mussau Islands**

Four single-phase models were created for site deposits on Eloaua and Emirau Islands (S6 Fig). Determinations from sites ECA and ECB were combined in a single-phase, island-scale model for Eloaua. Dating samples were identified and unidentified wood, charcoal, and bark; coconut (*Cocos nucifera*) endocarp; and identified marine shell. The early ceramics from the Mussau islands comprise red-slip, incised, circle-stamp, and dentate surface treatments [48, 49].

**ECA Site, Eloaua Island**

The initial appearance of ceramics at ECA is estimated as having occurred sometime between *3379-3055 cal BP (95.4%) or 3378-3152 cal BP (68.2%)*. The single-phase model contains 20 radiocarbon determinations obtained from unidentified charcoal (n = 6), identified wood or charcoal (n = 3), coconut endocarp (n =1) and marine shell of various taxa (n = 10). Two determinations are flagged as having low individual agreement values (ANU-5080, unidentified charcoal, 3260±90 BP; Beta-30678, *Chama* sp. marine shell, 3190±80 BP), but the A_model_ value remains high (above 90 for all runs). Therefore, these determinations were retained. (Removal of these determinations produces a cascading effect where additional determinations are identified as having low individual agreement values, the model agreement value drops [though stays above 60], and the start estimate date ranges become ~100+ years younger.)

Plot()

{

Outlier_Model("General",T(5),U(0,4),"t");

Outlier_Model("Charcoal", Exp(1,-10,0), U(0,3), "t");

Sequence("Mussau")

{

Boundary("start Mussau");

Phase("Mussau")

{

R_Date("ANU-5080", 3260, 90)

{

Outlier(1);

};

R_Date("Beta-30684", 3100, 110)

{

Outlier(1);

};

R_Date("Beta-20452", 3050, 70)

{

Outlier(1);

};

R_Date("Beta-30682", 2970, 50)

{

Outlier(1);

};

R_Date("ANU-5790", 2950, 80)

{

Outlier(1);

};

R_Date("Beta-20451", 2950, 70)

{

Outlier("General",0.05);

};

R_Date("ANU-5791", 2930, 80)

{

Outlier(1);

};

R_Date("Beta-30681", 2860, 60)

{

Outlier(1);

};

R_Date("Beta-30686", 2850, 70)

{

Outlier(1);

};

Curve("Marine13","Marine13.14c");

Delta_R("LocalMarine", -293,92);

R_Date("Beta-30680", 3320, 80)

{

Outlier("General",0.05);

};

R_Date("ANU-5084", 3190, 80)

{

Outlier("General",0.05);

};

R_Date("Beta-30678", 3190, 80)

{

Outlier("General",0.05);

};

R_Date("Beta-30677", 3170, 70)

{

Outlier("General",0.05);

};

R_Date("Beta-30683", 3140, 80)

{

Outlier("General",0.05);

};

R_Date("ANU-5085", 3130, 80)

{

Outlier("General",0.05);

};

R_Date("Beta-30674", 3110, 70)

{

Outlier("General",0.05);

};

R_Date("Beta-30675", 3110, 80)

{

Outlier("General",0.05);

};

R_Date("Beta-30679", 3080, 70)

{

Outlier("General",0.05);

};

R_Date("ANU-5081", 3010, 80)

{

Outlier("General",0.05);

};

R_Date("ANU-5082", 2950, 80)

{

Outlier("General",0.05);

};

};

Boundary("end Mussau");

};

};

**ECB Site, Eloaua Island**

The initial appearance of ceramics at the ECB site on Eloaua Island is estimated to have occurred sometime between *4221-3084 cal BP (95.4%) or 3612-3280 cal BP (68.2%)*. The single-phase model contains one radiocarbon determination obtained from unidentified charcoal and two radiocarbon determinations from marine shell. Such a small number of determinations results in an estimate with poor precision.

Plot()

{

Outlier_Model("General",T(5),U(0,4),"t");

Outlier_Model("Charcoal",Exp(1,-10,0),U(0,3),"t");

Sequence("ECB")

{

Boundary("start");

Phase("ECB")

{

R_Date("Beta-20453",3200,70)

{

Outlier(1);

};

Curve("Marine13","Marine13.14c");

Delta_R("LocalMarine", -293,92);

R_Date("ANU-5086",3120,80)

{

Outlier("General",0.05);

};

R_Date("ANU-5087",3150,80)

{

Outlier("General",0.05);

};

};

Boundary("end");

};

};

**Eloaua Island**

The determinations from the ECA and ECB models were combined as a single-phase, island-wide model for Eloaua. At this geographic scale, the appearance of pottery is estimated to have occurred sometime between *3513-3053 cal BP (95.4%) or 3453-3199 cal BP (68.2%)*.

Plot()

{

Outlier_Model("General",T(5),U(0,4),"t");

Outlier_Model("Charcoal", Exp(1,-10,0), U(0,3), "t");

Sequence("Mussau")

{

Boundary("start Mussau");

Phase("Mussau")

{

R_Date("ANU-5080", 3260, 90)

{

Outlier(1);

};

R_Date("Beta-20453",3200,70)

{

Outlier(1);

};

R_Date("Beta-30684", 3100, 110)

{

Outlier(1);

};

R_Date("Beta-20452", 3050, 70)

{

Outlier(1);

};

R_Date("Beta-30682", 2970, 50)

{

Outlier(1);

};

R_Date("ANU-5790", 2950, 80)

{

Outlier(1);

};

R_Date("Beta-20451", 2950, 70)

{

Outlier("General",0.05);

};

R_Date("ANU-5791", 2930, 80)

{

Outlier(1);

};

R_Date("Beta-30681", 2860, 60)

{

Outlier(1);

};

R_Date("Beta-30686", 2850, 70)

{

Outlier(1);

};

Curve("Marine13","Marine13.14c");

Delta_R("LocalMarine", -293,92);

R_Date("Beta-30680", 3320, 80)

{

Outlier("General",0.05);

};

R_Date("ANU-5084", 3190, 80)

{

Outlier("General",0.05);

};

R_Date("Beta-30678", 3190, 80)

{

Outlier("General",0.05);

};

R_Date("Beta-30677", 3170, 70)

{

Outlier("General",0.05);

};

R_Date("ANU-5087",3150,80)

{

Outlier("General",0.05);

};

R_Date("Beta-30683", 3140, 80)

{

Outlier("General",0.05);

};

R_Date("ANU-5085", 3130, 80)

{

Outlier("General",0.05);

};

R_Date("ANU-5086",3120,80)

{

Outlier("General",0.05);

};

R_Date("Beta-30674", 3110, 70)

{

Outlier("General",0.05);

};

R_Date("Beta-30675", 3110, 80)

{

Outlier("General",0.05);

};

R_Date("Beta-30679", 3080, 70)

{

Outlier("General",0.05);

};

R_Date("ANU-5081", 3010, 80)

{

Outlier("General",0.05);

};

R_Date("ANU-5082", 2950, 80)

{

Outlier("General",0.05);

};

};

Boundary("end Mussau");

};

};

**EQS Site, Emirau Island**

The initial appearance of ceramics at the EQS site on Emirau Island is estimated to have occurred sometime between *4088-3030 cal BP (95.4%) or 4088-3093 cal BP (68.2%)*. The single-phase model contains two radiocarbon determinations obtained from unidentified charcoal. Such a small number of determinations results in an estimate with poor precision. As the sole dated early ceramic site for Emirau, EQS is also the island-scale model.

Plot()

{

Outlier_Model("Charcoal", Exp(1,-10,0), U(0,3), "t");

Sequence("EQS")

{

Boundary("start");

Phase("EQS")

{

R_Date("Wk-21349",3044,31)

{

Outlier(1);

};

R_Date("Wk-21345",2917,31)

{

Outlier(1);

};

};

Boundary("end");

};

};

**References**

1.	Bellwood P, Dizon E. The Batanes Archaeological Project and the "Out of Taiwan" Hypothesis for Austronesian Dispersal. Journal of Austronesian Studies. 2005;1(1):1-33.

2.	Bellwood P, Dizon E. The Chronology of Batanes Prehistory. In: Bellwood P, Dizon E, editors. 4000 Years of Migration and Cultural Exchange. Canberra: The Australian National University; 2013. p. 68-75.

3.	Bellwood P, Anderson A, Dizon E. Archaeological Excavations on Batan Island. In: Bellwood P, Dizon E, editors. 4000 Years of Migration and Cultural Exchange. terra australis. 40. Canberra: The Australian National University; 2013. p. 31-45.

4.	Bellwood P, Stevenson J, Anderson A, Dizon E. Archaeological and palaeoenvironmental research in Batanes and Ilocos Norte Provinces, northern Philippines. Bulletin of the Indo-Pacific Prehistory Association. 2003;23:141-62.

5.	Hung H-c. Migration and Cultural Interaction in Southern Coastal China, Taiwan and the Northern Philippines, 3000 BC to AD 100: The Early History of the Austronesian speaking Populations [Thesis]: The Australian National University; 2008.

6.	Bellwood P, Dizon E, Mijares A. Archaeological Excavations on Itbayat and Siayan Islands. In: Bellwood P, Dizon E, editors. 4000 Years of Migration and Cultural Exchange: The Archaeology of the Batanes Islands, Northern Philippines2013. p. 9-29.

7.	Bellwood P, Dizon E. Archaeological Excavations at Savidug, Sabtang Island. In: Bellwood P, Dizon E, editors. 4000 Years of Migration and Cultural Exchange. terra australis. 40. Canberra: The Australian National University; 2013. p. 47-65.

8.	Casanova E, Knowles TDJ, Bayliss A, Dunne J, Barański MZ, Denaire A, et al. Accurate compound-specific 14C dating of archaeological pottery vessels. Nature. 2020;580(7804):506-10. doi: 10.1038/s41586-020-2178-z.

9.	Richard M, Maury R, Bellon H, Stephan J, Boirat J, Caldero A. Geology of Mt Iraya and Batan Island, northern Philippines. Philippine Journal of Volcanology. 1986;3(1):1-27.

10.	Bellwood P, Dizon E, De Leon A. The Batanes Pottery Sequence, 2500 BC to Recent. In: Bellwood P, Dizon E, editors. 4000 Years of Migration and Cultural Exchange: The Archaeology of the Batanes Islands, Northern Philippines. Terra Australis. 40. Canberra: The Australia National University; 2013. p. 77-113.

11.	Snow BE, Shutler R, Nelson DE, Vogel JS, Southon JR. Evidence of Early Rice Cultivation in the Philippines. Philippine Quarterly of Culture and Society. 1986;14(1):3-11.

12.	Ogawa H. Typological Crhonology of Pottery Assemblages from Lal-lo Shell Middens in Northern Luzon, Philippines. Journal of Southeast Asian Archaeology. 2005;25:1-29.

13.	Amano N. The faunal remains of Nagsabaran in Cagayan, Northern Philippines: Subsistence strategies in the Late Holocene [Thesis]: University of the Philippines; 2011.

14.	Hung H-C, Carson MT, Bellwood P, Campos FZ, Piper PJ, Dizon E, et al. The first settlement of Remote Oceania: the Philippines to the Marianas: supplementary information on the radiocarbon dating of the Nagsabaran site. Antiquity. 2011;85:909-26.

15.	Carson MT, Hung H-c. Learning from Paleo-Landscapes: Defining the Land-Use Systems of the Ancient Malayo-Polynesian Homeland. Current Anthropology. 2018;59:790-813. doi: 10.1086/700757.

16.	Mijares ASB. The Archaeology of Peñablanca Cave Sits, Northern Luzon, Philippines. Journal of Austronesian Studies. 2005;1(2):65-93.

17.	Bay-Petersen J. Excavations at Bagumbayan, Masbate, central Philippines: an economic analysis. Asian Perspectives. 1982;25(2):67-98.

18.	Hung H-C, Carson MT, Bellwood P, Campos FZ, Piper PJ, Dizon E, et al. The first settlement of Remote Oceania: the Philippines to the Marianas. Antiquity. 2011;85:909-26.

19.	Hutterer K, L. Test Excavations at the Edjek Site (TV161A) Tanjay, Negors Oriental. In: Hutterer K, L, Macdonald W, K, editors. Houses Built on Scattered Poles: Prehistory and Ecology in Negros, Oriental, Philippines. Cebu City: University of San Carlos; 1982. p. 209-26.

20.	Piper PJ, Campos FZ, Hung H-c. A study of the animal bone recovered from Pits 9 and 10 at the site of Nagsabaran in northern Luzon, Philippines. Hukay. 2009;14.

21.	Piper PJ, Hung H-c, Campos FZ, Bellwood P, Santiago R. A 4000 year-old introduction of domestic pigs into the Philippine Archipelago: implications for understanding routes of human migration through Island Southeast Asia and Wallacea. Antiquity. 2009;83(321):687-95.

22.	Solheim WG. The Batungan cave sites, Masbate, Philippines. Asian and Pacific Archaeology Series. 1968;2:21-62.

23.	Thiel B. Excavations at the Lal-lo shellmiddens, northeast Luzon, Philippines. Asian Perspectives. 1986;27(1):71-94.

24.	Tanaka K, Orogo AB. The archaeological excavation at the Pamittan site, Barangay Lanna, Solana, Cagayan Province, Philippines. 環境情報研究. 2000;(8):113-41.

25.	Spriggs M. Chronology of the Neolithic transition in Island Southeast Asia and the Western Pacific: a view from 2003. The Review of Archaeology. 2003;24(2):57-80.

26.	Snow B, Shutler R, Nelson D, Vogel J, Southon J. Evidence of Early Rice Cultivation in the Philippines. Philippine Quarterly of Culture and Society. 1986;14(1):3-11.

27.	Carson MT, Hung H-c, Summerhayes GR, Bellwood P. The Pottery Trail From Southeast Asia to Remote Oceania. The Journal of Island and Coastal Archaeology. 2013;8(1):17-36. doi: 10.1080/15564894.2012.726941.

28.	Bellwood P, editor. Archaeological Research in South-Eastern Sabah. Kota Kinabalu, Indonesia: Sabah Museum & Archives; 1988.

29.	Bellwood P. Archaeological Investigations at Bukit Tengkorak and Segarong, Southeastern Sabah. IPPA Bulletin. 1989;9:122-62.

30.	Datan I, Bellwood P. Recent Research at Gua Sireh (Serian) and Lubang Angin (Gunung Mulu National Park), Sarawak. Indo-Pacific Prehistory Association Bulletin. 1991;10:386-405.

31.	Plutniak S, Oktaviana AA, Sugiyanto B, Chazine JM, Ricaut FX. New ceramic data from East Kalimantan: Pottery chronology and the cord-marked and red-slipped sherds of Liang Abu’s layer 2. Journal of Pacific Archaeology. 2014;5(1):90-9.

32.	Simanjuntak T. New insight on the prehistoric chronology of Gunung Sewu, Java, Indonesia. Modern quaternary research in Southeast Asia. 2004;18:9-30.

33.	Anggraeni. The Austronesian Migration Hypothesis as Seen from Prehistoric Settlements on the Karama River, Mamuju, West Sulawesi [Thesis]: The Australian National University; 2012.

34.	Anggraeni, Simanjuntak T, Bellwood P, Piper P. Neolithic foundations in the Karama valley, West Sulawesi, Indonesia. Antiquity. 2014;88(341):740-56. Epub 2014/08/26. doi: 10.1017/S0003598X00050663.

35.	Mahmud I. The Neolithic Site of Mallawa. In: Simanjuntak T, editor. Austronesian in Sulawesi. Yogyakarta, Indonesia: Center for Prehistoric and Austronesian Studies; 2008. p. 119-28.

36.	Simanjuntak T, editor. Austronesian in Sulawesi. Indonesia: Center for Prehistoric and Austronesian Studies; 2008.

37.	Simanjuntak T, Morwood MJ, Intan FS, Machmud I, Grant K, Somba N, et al. Minanga Sipakko and the Neolithic of the Karama River. In: Simanjuntak T, editor. Austronesian in Sulawesi. Indonesia: Center for Prehistoric and Austronesian Studies; 2008. p. 57-76.

38.	Azis N, Reepmeyer C, Clark G, Sriwigati, Tanudirjo DA. Mansiri in North Sulawesi: A new dentate-stamped pottery site in Island Southeast Asia. In: O'Connor S, Bulbeck D, Meyer J, editors. The Archaeology of Sulawesi: Current Research on the Pleistocene to the Historic Period. Acton, Australia: The Australian National University; 2018. p. 191-205.

39.	Ono R, Octavianus Sofian H, Aziz N, Sriwigati, Oktaviana AA, Alamsyah N, et al. Traces of Early Austronesian Expansion to East Indonesia? New Discovery of Dentate-Stamped and Lime-Infilled Pottery from Central Sulawesi. The Journal of Island and Coastal Archaeology. 2019;14(1):123-9. doi: 10.1080/15564894.2018.1481897.

40.	Brooks ST, Heglar R, Brooks RH. Radiocarbon Dating and Palaeoserology of a Selected Burial Series from the Great Cave of Niah, Sarawak, Malaysia. Asian Perspectives. 1977;20(1):21-31.

41.	Anggraeni, Sunarningsih. The Prehistoric Settlement at Jambu Hilir, South Kalimantan Province, Indonesia. IPPA Bulletin. 2008;28:120-6.

42.	Azis N, Reepmeyer C, Clark G, Sriwigati, Tanudirjo D. Mansiri in North Sulawesi: A new dentate-stamped pottery site in Island Southeast Asia. In: S OC, D B, J M, editors. The Archaeology of Sulawesi: Current Research on the Pleistocene to the Historic Period. Canberra: Australian National University; 2018. p. 191-205.

43.	O’Connor S, Barham A, Spriggs M, Veth P, Aplin K, St Pierre E. Cave Archaeology and Sampling Issues in the Tropics: A Case Study from Lene Hara Cave, a 42,000 Year Old Occupation Site in East Timor, Island Southeast Asia. Australian Archaeology. 2010;71(1):29-40. doi: 10.1080/03122417.2010.11689382.

44.	Galipaud J-C, Kinaston R, Halcrow S, Foster A, Harris N, Simanjuntak T, et al. The Pain Haka burial ground on Flores: Indonesian evidence for a shared Neolithic belief system in Southeast Asia. Antiquity. 2016;90(354):1505-21. Epub 2016/11/21. doi: 10.15184/aqy.2016.185.

45.	Lape P, Peterson E, Tanudirjo D, Shiung C-C, Lee G-A, Field J, et al. New Data from an Open Neolithic Site in Eastern Indonesia. Asian Perspectives. 2018;57(2):222-43.

46.	Bellwood P, Nitihaminoto G, Irwin G, Gunadi, Waluyo A, Tanudirjo D. 35,000 years of prehistory in the northern Moluccas. In: Bartstra G-J, editor. Bird's Head Approaches; Irian Jaya Studies - A Programme for Interdisciplinary Research. Rotterdam: A. A. Balkema; 1998. p. 233-75.

47.	Bellwood P. Archaeological Research in Minahasa and the Talaud Islands, Northeastern Indonesia. Asian Perspectives. 1976;19(2):240-88.

48.	Summerhayes G, Matisoo-Smith E, Mandui H, Allen J, Specht J, Hogg N, et al. Tamuarawai (EQS): An Early Lapita Site on Emirau, New Ireland, PNG. Journal of Pacific Archaeology. 2010;1(1):62-75.

49.	Kirch PV, editor. Lapita and its Transformations in Near Oceania: Archaeological Investigations in the Mussau Islands, Papua New Guinea, 1985-88, Volume I, Introduction, Stratigraphy, Chronology. Berkeley: University of California; 2001.

50.	Simanjuntak T. The Western Route Migration: A Second Probable Neolithic Diffusion to Indonesia. In: Piper PJ, Matsumae H, Bulbeck D, editors. New Perspectives in Southeast Asian and Pacific Prehistory. Canberra: Australian National University Press; 2017. p. 201-11.

51.	Spriggs M. Archaeology and the Austronesian expansion: where are we now? . Antiquity. 2011;85(328):510-28.

52.	Chia S. The Prehistory of Bukit Tengkorak, Sabah, Malaysia. Journal of Southeast Asian Archaeology. 2001;21:146-59.

53.	Bellwood P, Waluyo A, Gunadi, Nitihaminoto G, irwin G. Archaeological Research in the Northern Moluccas; Interim Resultss, 1991 Field Season. Bulletin of the Indo-Pacific Prehistory Association. 1993;13:19-33.

54.	Tanudirjo DA. Islands in between: prehistory of the northeastern Indonesian archipelago [Thesis]: The Australian National University; 2001.

55.	Denham TP, Ramsey CB, Specht J. Dating the appearance of Lapita pottery in the Bismarck Archipelago and its dispersal to Remote Oceania. Archaeology in Oceania. 2012;47(1):39-46. PubMed PMID: 73820639.

56.	Kirch PV. A Radiocarbon Chronology for the Mussau Islands. In: Kirch PV, editor. Lapita and its Transformations in Near Oceania: Archaeological Investigations in the Mussau Islands, Papua New Guinea, 1985-88, Volume I, Introduction, Stratigraphy, Chronology. Berkeley: University of California; 2001. p. 196-222.

57.	Petchey F, Ulm S. Marine Reservoir Variation in the Bismarck Region: an Evaluation of the Spatial and Temporal Change in ­­Delta R Over the Last 3000 Years. Radiocarbon. 2012;54(1):45-58. doi: DOI 10.2458/azu_js_rc.v54i1.13050. PubMed PMID: WOS:000307006800006.

**Supporting Information Figures and Tables**

S1 Fig. Modeled Date Range Distributions for the Appearance of Pottery in the Batanes Islands. Lines beneath each distribution delimit 95.4% and 68.2% HPD.

**S2 Fig.** **Modeled Date Range Distributions for the Appearance of Pottery on Luzon Island, northern Philippines.** Lines beneath each distribution delimit 95.4% and 68.2% HPD.

**S3 Fig.** **Modeled Date Range Distributions for the Appearance of Pottery on Borneo, Sulawesi, and Java islands.** Note that the x axis differs from the other figures. Lines beneath each distribution delimit 95.4% and 68.2% HPD.

**S4 Fig.** Modeled Date Range Distributions for the Appearance of Pottery in the Lesser Sunda Islands. Lines beneath each distribution delimit 95.4% and 68.2% HPD.

**S5 Fig.** Modeled Date Range Distributions for the Appearance of Pottery in the Molucca Islands. Lines beneath each distribution delimit 95.4% and 68.2% HPD.

**S6 Fig.** Modeled Date Range Distributions for the Appearance of Pottery in the Mussau islands. Lines beneath each distribution delimit 95.4% and 68.2% HPD.

S1 Table. Order analysis of the island-scale models for the initial deposition of pottery. Probability that t1 is older than t2; >0.5 then older, <0.5 then younger

| **Probability *t*_1_ < *t*_2_** |  |  |  |  |  |  |  |  |  |  |  |  |  |  |
| --- | --- | --- | --- | --- | --- | --- | --- | --- | --- | --- | --- | --- | --- | --- |
| ***t*_1_** | ***t*_2_** | | | | | | | | | | | | | |
|  | **Borneo** | **Itbayat** | **Timor** | **Luzon** | **Java** | **Sulawesi** | **Emirau** | **Pulau Ay** | **Eloaua** | **Batan (w/ residue)** | **Sabtang** | **North Borneo** | **Flores** | **Batan (no residue)** |
| **Borneo** | 0 | 0.679 | 0.6281 | 0.868 | 0.8046 | 0.7679 | 0.9947 | 0.9962 | 1 | 0.9984 | 0.9868 | 0.959 | 1 | 0.9984 |
| **Itbayat** | 0.321 | 0 | 0.5433 | 0.803 | 0.7414 | 0.7274 | 0.9836 | 0.9898 | 0.9997 | 0.9957 | 0.9744 | 0.9426 | 1 | 0.9971 |
| **Timor** | 0.3719 | 0.4567 | 0 | 0.7389 | 0.7414 | 0.7409 | 0.9463 | 0.9803 | 0.9992 | 0.9922 | 0.9573 | 0.9374 | 0.9998 | 0.9958 |
| **Luzon** | 0.13204 | 0.19704 | 0.26111 | 0 | 0.589 | 0.62 | 0.8589 | 0.9448 | 0.9957 | 0.977 | 0.914 | 0.8945 | 0.9997 | 0.9905 |
| **Java** | 0.19541 | 0.25858 | 0.25862 | 0.411 | 0 | 0.561 | 0.7411 | 0.8104 | 0.8846 | 0.9012 | 0.8486 | 0.8511 | 0.9873 | 0.9705 |
| **Sulawesi** | 0.23208 | 0.27264 | 0.25912 | 0.38 | 0.439 | 0 | 0.5997 | 0.6463 | 0.6865 | 0.7217 | 0.694 | 0.7438 | 0.8726 | 0.8703 |
| **Emirau** | 0.005343 | 0.016361 | 0.05372 | 0.14109 | 0.25886 | 0.4003 | 0 | 0.5678 | 0.652 | 0.7468 | 0.699 | 0.7482 | 0.9773 | 0.9405 |
| **Pulau Ay** | 0.00381 | 0.010248 | 0.019722 | 0.05517 | 0.18962 | 0.3537 | 0.4322 | 0 | 0.6115 | 0.7121 | 0.6597 | 0.7164 | 0.9585 | 0.922 |
| **Eloaua** | 0 | 0.000251 | 0.000816 | 0.004341 | 0.1154 | 0.31348 | 0.348 | 0.3885 | 0 | 0.6703 | 0.6332 | 0.7016 | 0.9885 | 0.9328 |
| **Batan (w/ residue)** | 0.001576 | 0.004303 | 0.007781 | 0.023014 | 0.09882 | 0.2783 | 0.25318 | 0.28789 | 0.3297 | 0 | 0.5064 | 0.6379 | 0.96 | 0.8942 |
| **Sabtang** | 0.013185 | 0.025602 | 0.04266 | 0.086 | 0.15143 | 0.30596 | 0.30103 | 0.3403 | 0.3668 | 0.4936 | 0 | 0.6446 | 0.9549 | 0.8901 |
| **North Borneo** | 0.04103 | 0.05737 | 0.06262 | 0.10548 | 0.14892 | 0.25618 | 0.25176 | 0.28363 | 0.29837 | 0.3621 | 0.3554 | 0 | 0.6706 | 0.696 |
| **Flores** | 0.000003 | 0.000039 | 0.000151 | 0.000271 | 0.012739 | 0.12741 | 0.022699 | 0.04149 | 0.011541 | 0.04001 | 0.04506 | 0.3294 | 0 | 0.6256 |
| **Batan (no residue)** | 0.001638 | 0.002929 | 0.004189 | 0.009452 | 0.029511 | 0.12966 | 0.05953 | 0.07804 | 0.0672 | 0.10578 | 0.10993 | 0.304 | 0.3744 | 0 |

S2 Table. Order analysis of the site-scale models for the initial deposition of pottery. Probability that t1 is older than t2; >0.5 then older, <0.5 then younger.

| **Probability *t*_1_ < *t*_2_** |  |  |  |  |  |  |  |  |  |  |  |  |  |  |  |  |  |  |  |  |  |
| --- | --- | --- | --- | --- | --- | --- | --- | --- | --- | --- | --- | --- | --- | --- | --- | --- | --- | --- | --- | --- | --- |
| ***t*_1_** | ***t*_2_** | | | | | | | | | | | | | | | | | | | | |
|  | **MAD1** | **Gua Sireh** | **Torongan Cave** | **Malawa** | **Nagsabaran** | **Lena Hara** | **Andarayan** | **Minango Sipakko** | **Irigayen** | **ECB** | **EQS** | **PA1** | **Sunget** | **ECA** | **Savidug** | **Bukit Tengkorak** | **Naidi** | **Anaro Hilltop** | **Pain Haka** | **Payaman** | **Liang Abu** |
| **MAD1** | 0 | 0.5933 | 0.656 | 0.6429 | 0.6895 | 0.6587 | 0.7291 | 0.7444 | 0.8275 | 0.8226 | 0.8349 | 0.8471 | 0.8398 | 0.8615 | 0.8615 | 0.8419 | 0.8583 | 0.9003 | 0.9294 | 0.9086 | 0.9991 |
| **Gua Sireh** | 0.4067 | 0 | 0.5334 | 0.5958 | 0.5792 | 0.6986 | 0.8023 | 0.736 | 0.9965 | 0.9816 | 0.9971 | 0.9976 | 0.9721 | 1 | 0.9914 | 0.8807 | 0.9066 | 0.9973 | 1 | 0.9534 | 1 |
| **Torongan Cave** | 0.344 | 0.4666 | 0 | 0.5585 | 0.5689 | 0.6751 | 0.7846 | 0.7122 | 0.9964 | 0.9804 | 0.997 | 0.9972 | 0.9701 | 1 | 0.9909 | 0.8772 | 0.9035 | 0.9972 | 1 | 0.9512 | 1 |
| **Malawa** | 0.3571 | 0.4042 | 0.4415 | 0 | 0.5244 | 0.5606 | 0.6772 | 0.6879 | 0.9067 | 0.8951 | 0.9049 | 0.9264 | 0.8976 | 0.9456 | 0.9235 | 0.8228 | 0.8522 | 0.9572 | 0.9753 | 0.9015 | 1 |
| **Nagsabaran** | 0.31049 | 0.4208 | 0.4311 | 0.4756 | 0 | 0.5547 | 0.6581 | 0.6377 | 0.891 | 0.8789 | 0.8959 | 0.9169 | 0.8947 | 0.9405 | 0.9304 | 0.8233 | 0.8529 | 0.9703 | 0.9965 | 0.9193 | 1 |
| **Lena Hara** | 0.3413 | 0.30138 | 0.3249 | 0.4394 | 0.4453 | 0 | 0.6541 | 0.681 | 0.9633 | 0.9501 | 0.946 | 0.9803 | 0.935 | 0.9994 | 0.9572 | 0.8134 | 0.8491 | 0.99 | 0.9999 | 0.89 | 1 |
| **Andarayan** | 0.27095 | 0.19772 | 0.21541 | 0.3228 | 0.3419 | 0.3459 | 0 | 0.6171 | 0.9015 | 0.8908 | 0.8729 | 0.9461 | 0.8903 | 0.9968 | 0.9243 | 0.761 | 0.802 | 0.9794 | 0.9997 | 0.8489 | 1 |
| **Minango Sipakko** | 0.25565 | 0.26395 | 0.28779 | 0.31214 | 0.3623 | 0.319 | 0.3829 | 0 | 0.5703 | 0.5588 | 0.6017 | 0.6378 | 0.6392 | 0.6873 | 0.7124 | 0.6819 | 0.7167 | 0.8401 | 0.944 | 0.8039 | 1 |
| **Irigayen** | 0.17246 | 0.003496 | 0.003592 | 0.09329 | 0.10896 | 0.03671 | 0.0985 | 0.4297 | 0 | 0.5005 | 0.569 | 0.6757 | 0.6646 | 0.8568 | 0.7646 | 0.6045 | 0.6558 | 0.9087 | 0.9948 | 0.732 | 1 |
| **ECB** | 0.17745 | 0.018449 | 0.019617 | 0.10489 | 0.12109 | 0.04988 | 0.10916 | 0.4412 | 0.4995 | 0 | 0.5843 | 0.6845 | 0.6836 | 0.8834 | 0.7843 | 0.6146 | 0.6648 | 0.92 | 0.9972 | 0.7388 | 1 |
| **EQS** | 0.16507 | 0.002921 | 0.002974 | 0.09506 | 0.10413 | 0.05395 | 0.1271 | 0.3983 | 0.431 | 0.4157 | 0 | 0.5681 | 0.5868 | 0.6999 | 0.6999 | 0.5816 | 0.6323 | 0.8643 | 0.9773 | 0.7229 | 1 |
| **PA1** | 0.1529 | 0.002432 | 0.002765 | 0.07361 | 0.08309 | 0.019683 | 0.05388 | 0.3622 | 0.3243 | 0.31552 | 0.4319 | 0 | 0.536 | 0.6732 | 0.6611 | 0.5533 | 0.6039 | 0.8365 | 0.9587 | 0.7052 | 1 |
| **Sunget** | 0.16018 | 0.027943 | 0.029868 | 0.10239 | 0.10526 | 0.06503 | 0.10967 | 0.3608 | 0.3354 | 0.3164 | 0.4132 | 0.464 | 0 | 0.5711 | 0.6348 | 0.5611 | 0.6098 | 0.8299 | 0.98 | 0.7143 | 1 |
| **ECA** | 0.13854 | 0.000002 | 0.000016 | 0.05435 | 0.05947 | 0.000596 | 0.00322 | 0.31274 | 0.14316 | 0.11657 | 0.30005 | 0.3268 | 0.4289 | 0 | 0.6062 | 0.5235 | 0.573 | 0.823 | 0.9874 | 0.6852 | 1 |
| **Savidug** | 0.13849 | 0.008637 | 0.009144 | 0.07647 | 0.06962 | 0.0428 | 0.07569 | 0.28759 | 0.23539 | 0.21568 | 0.30014 | 0.3389 | 0.3652 | 0.3938 | 0 | 0.5089 | 0.5581 | 0.7464 | 0.9555 | 0.687 | 1 |
| **Bukit Tengkorak** | 0.15808 | 0.11927 | 0.12276 | 0.17719 | 0.17673 | 0.18659 | 0.23902 | 0.3181 | 0.3955 | 0.3854 | 0.4184 | 0.4467 | 0.4389 | 0.4765 | 0.4911 | 0 | 0.5429 | 0.6034 | 0.6932 | 0.7059 | 0.9947 |
| **Naidi** | 0.14172 | 0.0934 | 0.09646 | 0.14777 | 0.14713 | 0.15091 | 0.19798 | 0.28326 | 0.3442 | 0.3352 | 0.3677 | 0.3961 | 0.3902 | 0.427 | 0.4419 | 0.4571 | 0 | 0.554 | 0.6433 | 0.6591 | 0.9747 |
| **Anaro Hilltop** | 0.09968 | 0.002737 | 0.002805 | 0.04277 | 0.029723 | 0.009966 | 0.020604 | 0.15988 | 0.09129 | 0.07997 | 0.13567 | 0.16349 | 0.17014 | 0.17696 | 0.25359 | 0.3966 | 0.446 | 0 | 0.733 | 0.6369 | 0.9999 |
| **Pain Haka** | 0.07055 | 0.000008 | 0.000009 | 0.024743 | 0.00346 | 0.000115 | 0.000297 | 0.05604 | 0.005157 | 0.002837 | 0.022674 | 0.04134 | 0.020041 | 0.012594 | 0.04453 | 0.30676 | 0.3567 | 0.26701 | 0 | 0.6028 | 1 |
| **Payaman** | 0.09142 | 0.04659 | 0.04883 | 0.09848 | 0.08074 | 0.11004 | 0.15111 | 0.19608 | 0.26801 | 0.26115 | 0.2771 | 0.2948 | 0.28569 | 0.31484 | 0.31302 | 0.29414 | 0.3409 | 0.3631 | 0.3972 | 0 | 0.8971 |
| **Liang Abu** | 0.000864 | 0 | 0 | 0 | 0 | 0 | 0 | 0 | 0 | 0 | 0 | 0.000032 | 0 | 0 | 0 | 0.00533 | 0.025345 | 0.000143 | 0 | 0.10285 | 0 |

**S3 Table.** **Radiocarbon sample data**.

| **Island Group** | **Island/region** | **Provenience** | **Laboratory number** | **Sample details*** | **Radiocarbon age (BP)** | **Error** | **δ13C (‰)** | **Dating Method**† | **Notes** | **Ceramic**  **Surface Modifications** | **Reference** |
| --- | --- | --- | --- | --- | --- | --- | --- | --- | --- | --- | --- |
| Batanes | Batan | Mahatao Town Septic Tank, Auger M3, cultural palaeosol at 250 cmbs below volcanic ash (with sherds) | ANU-11710 | UC | 2090 | 60 | - | A | Sample recovered from auger not controlled excavation. | red slip | [3:45, 4:149] |
| Batanes | Batan | Naidi (Basco-Songsong Road), 0-10 cm, charcoal in north road section | ANU-11695 | UC | 2620 | 30 | - | C | "Excluding the presumably out of context ANU 11709, the remaining dates give a calibrated age range of between 835 BC to AD 900 - in actuality a much wider range that can be accommodated by such a shallow archaeological deposit. The main concentration of dates points to the late first millennium BC for the Naidi assemblage" [4:149]. "The closest parallels to these Naidi Phase assemblages discussed so far lie in the lower cultural layer at Savidug Dune Site and at Anaro, but the absence of stamped circles clearly places the Naidi pottery later than the Phase 2 material from these two sites." [2:111]. | red slip | [2:71, Table 5.1, 4] |
| Batanes | Batan | Naidi (Basco-Songsong Road), 0-10 cm, charcoal in south road section | ANU-11694 | UC | 1590 | 210 | - | C | See ANU-11695 | red slip | [2:71, Table 5.1, 4] |
| Batanes | Batan | Naidi (Basco-Songsong Road), Pit A1, 0-10 cm (within layer) | ANU-11709 | UC | 200 | 360 | - | C | This date is anomalous. The paleosol is capped by volcanic ash that was deposited between 1500-1000 BP. | red slip | [2:71, Table 5.1, 4] |
| Batanes | Batan | Naidi (Basco-Songsong Road), Pit A2, 0-10 cm | ANU-11708 | UC | 2240 | 140 | - | C | See ANU-11695 | red slip | [2:71, Table 5.1, 4] |
| Batanes | Batan | Payaman, north square, Layer III, 10-25 cm within layer | Wk-13092 | UC | 1988 | 47 | - | C |  | red slip | [2: 71, Table 5.1, 4:150] |
| Batanes | Batan | Payaman, south square, Layer III, 20-25 cm within layer (based on profile photograph, this should be almost at the base of the unit) | ANU-12068 | UC | 1486 | 185 | - | C |  | red slip | [2:71, Table 5.1, 4:150] |
| Batanes | Batan | Sunget Main Terrace, Layer V, 15-20 cm (within layer) | OZH-776 | Resin coating on sherd exterior | 5790 | 150 | - | A | Author suggests date might not date sherds and be from "fossil resin" on sherd. | red slip, circle-stamped | [2: 71, Table 5.1, 4:150] |
| Batanes | Batan | Sunget Main Terrace, Layer V, 15-20 cm (within layer) | Wk-14640 | Residue on sherd | 2915 | 49 | -26 | A |  | red slip, circle-stamped | [1:14, 2:70, Table 5.1] |
| Batanes | Batan | Sunget Main Terrace, Layer V, 20-30 cm (within layer) | ANU-11817 | Residue on sherd | 2910 | 190 | - | A |  | red slip, circle-stamped | *68:70, Table 5.1, 69:14*) |
| Batanes | Batan | Sunget Main Terrace, Layer V, 30-35 cm (within layer, scattered charcoal fragments) | Wk-15649 | UC | 2383 | 35 | -25.9 | A | Author suggests charcoal dates from more recent use of site, possibly from digging sticks and charcoal. | red slip, circle-stamped | *68:70, Table 5.1, 69:14*) |
| Batanes | Batan | Sunget Top Terrace, Layer V, 20-30 cm, charcoal concentration (possible hearth) | ANU-11693 | UC | 2630 | 30 | -27.0 | C | Author suggests may date from later activity at site | red slip, circle-stamped | *68:70, Table 5.1, 69:14*) |
| Batanes | Batan | Sunget Top Terrace, Squares A/D, Layer V, 20-30 cm (within layer) | ANU-11707 | UC | 2000 | 140 | -27.6 | A | Author suggests charcoal dates from more recent use of site, possibly from digging sticks and charcoal. | red slip, circle-stamped | [2:70, Table 5.1, 5:131] |
| Batanes | Batan | Tayid, beneath main ash deposit | ANU-12069 | Residue on sherd | 1842 | 215 | - | A |  | red slip | [2: 70, Table 5.1] |
| Batanes | Itbayat | Anaro Hilltop Site, Area 2A, 15-20 cm | Wk-14643 | Residue on sherd | 1876 | 41 | -26 | A | Bellwood & Dizon [2: Table 5.3] indicate Anaro ceramic period is 1200 BC - 600 AD | circle-stamped | [2: 70, Table 5.1] |
| Batanes | Itbayat | Anaro Hilltop Site, Area 3, 90-95 cm | Wk-14645 | Residue on sherd | 1360 | 39 | -23.6 | A | See Wk-14643 | circle-stamped | [2: 70, Table 5.1] |
| Batanes | Itbayat | Anaro Hilltop Site, Area 3, 95-105 cm | OZH-774 | Residue on sherd | 2770 | 50 | -23.6 | A | See Wk-14643 | circle-stamped | [2: 70, Table 5.1] |
| Batanes | Itbayat | Anaro Hilltop Site, Area 3A (inside rockshelter), 100-110 cm | OZJ-693 | Residue on sherd | 2475 | 45 | -24 | C | See Wk-14643 | circle-stamped | [2: 70, Table 5.1] |
| Batanes | Itbayat | Anaro Hilltop Site, Area 3A (inside rockshelter), 80-90 cm | OZJ-692 | Residue on sherd | 2095 | 45 | -25.2 | C | See Wk-14643 | circle-stamped | [2: 70, Table 5.1] |
| Batanes | Itbayat | Anaro Hilltop Site, Area 3B, 70-75 cm | OZJ-695 | Residue on sherd | 2080 | 45 | -26.3 | C | See Wk-14643 | circle-stamped | [2: 70, Table 5.1] |
| Batanes | Itbayat | Anaro Hilltop Site, Area 3B, 85-90 cm | OZJ-696 | Residue on sherd | 1375 | 45 | -25.9 | C | Bellwood et al. [10:83] argue that these young dates are the result of the movement of younger material traveling downwards into the profile. | circle-stamped | [2: 70, Table 5.1] |
| Batanes | Itbayat | Anaro Hilltop Site, Area 3B, 90-95 cm | OZJ-697 | Residue on sherd | 1510 | 60 | -24.4 | C | See OZJ-696 | circle-stamped | [2: 70, Table 5.1] |
| Batanes | Itbayat | Anaro Hiltop Site, Area 3B, 65-70 cm | OZJ-694 | Residue on sherd | 1280 | 45 | -20.7 | C | See Wk-14643 | circle-stamped | [2: 70, Table 5.1] |
| Batanes | Batan | Basco | Gif-6575 | UC | 2310 | 80 | - |  | Cannot be clearly used for determining ceramic chronology; included in dataset for information | - | [9:13] |
| Batanes | Itbayat | Torongan cave, Squares A-D, 55-60 cm (base of cultural layer) | OZH771 | Residue on sherd | 3860 | 70 | - |  | Shell dates from deposit not used as could be washed into cave from above | red-slip | [3: chapters 3, 5] |
| Batanes | Itbayat | Torongan cave, Squares A-D, 55-60 cm (base of cultural layer) | Wk14642 | Residue on sherd | 3320 | 40 | -25.3 |  |  | red-slip | [3: chapters 3, 5] |
| Batanes | Sabtang | Savidug dune, trench R7-9, layer 5, 220 cmbs | Wk21810 | Residue on sherd | 2870 | 30 | -25.8 |  |  | red-slip | [3: chapters 3, 5] |
| Batanes | Sabtang | Savidug dune, trench R7-9, layer 5, 210 cmbs | Wk21808 | UC | 2146 | 30 | -26.9 |  |  | red-slip | [3: chapters 3, 5] |
| Batanes | Sabtang | Savidug dune, 2006 unit 5, layer 5, 180 cmbs | Wk19711 | UC | 2828 | 37 | -26.7 |  | Exact association between charcoal and sherds unclear. Bellwood & Dizon [10:100]state sherds "were stratified just above the radiocarbon date of c.1000 BC (Wk 19711, Table 5.1)". Date comes from Layer 5, but sherds, apparently, in Layer 4. | red-slip, circle-stamped | [3: chapters 3, 5] |
| Northern Philippines | Luzon | Nagsabaran, TP7, 160 cm | NTU3798 | UC | 2670 | 40 | - |  | Hung et al. [18] argue that some determinations are too recent and thus must be out of context. | red-slip, incised, impressed, dentate | [13, 14] |
| Northern Philippines | Luzon | Nagsabaran, TP7, 160 cm | GX28379 | UC | 3050 | 70 | - |  | See NTU3798 | red-slip, incised, impressed, dentate | [13, 14] |
| Northern Philippines | Luzon | Nagsabaran, TP7, 190 cm | GX28381 | UC | 3390 | 130 | - |  | See NTU3798 | red-slip, incised, impressed, dentate | [13, 14] |
| Northern Philippines | Luzon | Nagsabaran, TP9, 150 cm | Wk-19712 | Animal bone | 2504 | 35 | - | C | Transition between basal and middle silt layer; WK-19712 is stratigraphically superior to WK-18059; date seems anomalous. | red-slip, incised, impressed, dentate | [5:159, Table 7.1] |
| Northern Philippines | Luzon | Nagsabaran, TP9, 150 cm | Wk-19713 | UC | 4450 | 39 | - | C | Transition between basal and middle silt layer; WK-19713 and 19712 should agree (same context); WK-19713 is stratigraphically superior to WK-17756 and WK-18059; date seems anomalous. | red slip, incised, impressed, dentate | [5:159, Table 7.1] |
| Northern Philippines | Luzon | Nagsabaran, TP9, 160 cm | Wk-18059 | UC | 1946 | 30 | - | C | Upper margin of basal silt layer (based on profile) | red slip, incised, impressed, dentate | [5:159, Table 7.1] |
| Northern Philippines | Luzon | Nagsabaran, TP9, 180 cm | Wk-17756 | UC | 2528 | 31 | - | C | Lower margin of basal silt layer (based on profile) | red slip, incised, impressed | [5:159, Table 7.1] |
| Northern Philippines | Luzon | Nagsabaran, TP9, Level 14, 130-140 cm | Wk-23397 | *Sus scrofa/ verrucosus* lower fourth premolar (Cat. 2004-12-16) | 3940 | 40 | - | C | Piper et al. [20:78] state tooth originates from TP 9, level 14 (130-140 bmg [below modern ground level]; this sample is stratigraphically superior to all other samples in TP 9 yet has the oldest date in the sequence. | red slip, incised, impressed, circle-stamped, dentate | [20:78, 21:691] |
| Northern Philippines | Luzon | Nagsabaran, TP11, 170 cm | ANU13016 | UC | 3510 | 30 | -26.5 |  | See NTU3798 | red slip, incised, impressed | [13, 14] |
| Northern Philippines | Luzon | Nagsabaran, TP14, 240 cm | ANU13014 | UC | 2660 | 30 | -27.4 |  | See NTU3798 | red slip, incised, impressed, circle-stamped, dentate | [13, 14] |
| Northern Philippines | Luzon | Nagsabaran, TP14, 240 cm | ANU13013 | UC | 2540 | 30 | -31.6 |  | See NTU3798 | red slip, incised, impressed, circle-stamped, dentate | [13, 14] |
| Northern Philippines | Luzon | Andarayan Site | SFU-86 | UC | 3240 | 160 | - | C |  | red slip | [11:5] |
| Northern Philippines | Luzon | Andarayan Site |  | Rice husk and stem fragments | 3400 | 125 | -24 | A | Extracted from earthenware jar that contained rice husk and stem portion inclusions in the temper. | red slip | [11:5] |
| Northern Philippines | Luzon | Gaerlan Shell Midden | NUTA2-7938 | Animal bone | 3810 | 30 | - | A |  | red slip | [12:30] |
| Northern Philippines | Luzon | Gaerlan Shell Midden | NUTA2-7939 | Animal bone | 3485 | 30 | - | A |  | red slip | [12:30] |
| Northern Philippines | Luzon | Gaerlan Shell Midden | NUTA2-7940 | Animal bone | 3665 | 35 | - | A |  | red slip | [12:30] |
| Northern Philippines | Luzon | Irigayen | NUTA2-914 | UC | 3025 | 20 | - | C |  | red slip, punctate | [12:30] |
| Northern Philippines | Luzon | Irigayen | NUTA2-912 | UC | 2925 | 20 | - | C |  | red slip, punctate | [12:30] |
| Northern Philippines | Luzon | Irigayen | NUTA2-913 | UC | 3165 | 25 | - | C |  | red slip, punctate | [12:30] |
| Northern Philippines | Luzon | Irigayen | NUTA2-917 | UC | 3185 | 25 | - | C |  | red slip, punctate | [12:30] |
| Northern Philippines | Luzon | Lal-lo Shell Midden Site, TU 12S/46E, Level 6, 85 cm | Gak-7048 | Shell (not indicated whether marine or terrestrial) | 3680 | 110 | - | C |  | red slip, incised, impressed | [23:80] |
| Northern Philippines | Luzon | Callao cave, Layer 4, 40 cmbs | Wk17010 | UC | 3335 | 34 | - | A |  | red-slip, incised, impressed | [16] |
| Northern Philippines | Luzon | Dalan Serkot cave, 44 cmbs | Wk15648 | UC | 3530 | 34 | - | A |  | red-slip, black with incising | [16] |
| Northern Philippines | Luzon | Dalan Serkot cave, 70 cmbs | Wk14879 | UC | 6124 | 48 | - | A |  | preceramic | [16] |
| Northern Philippines | Luzon | Pamittan, Square N59E6, Layer II (lower level) | Gak-17967 | UC | 3390 | 100 | - | C | association of charcoal and ceramics is unknown given farming field context in valley | earthenware | [24:132, 25:67] |
| Northern Philippines | Luzon | Pamittan, SquareN59E6, Layer III (71 cm bd) | Gak-17968 | UC | 3810 | 200 | - | C | association of charcoal and ceramics is unknown given farming field context in valley | earthenware | [24:132, 25:67] |
| Central Philippines | Negros | Edjek site, Layer IV-V interface, 117-120 cmbs | Beta1117 | UC | 3475 | 235 | - |  |  | incised, impressed, orange-slip | [19] |
| Central Philippines | Masbate | Bagumbayan Site, Layer IV (lowest spit) |  |  | 3510 | 90 | - | C | Dates from the Harwell Low Level Measurements Laboratory | plain | [17] |
| Central Philippines | Masbate | Bagumbayan Site, Layer V (top spit) |  |  | 3620 | 100 | - | C | Dates from the Harwell Low Level Measurements Laboratory | plain | [17] |
| Central Philippines | Masbate | Cave 2, 12-18 inch level | L274 (Lamont laboratory) | UC | 2700 | 100 | - | C | Sherd assemblage also in excavation levels below this date; date is not earliest pottery deposit at site | incised, impressed | [22] |
| Greater Sunda Islands | Borneo/Kalimantan | Liang Abu, Layer 2 | UBA20839 | UC | 1672 | 21 | -32.5 |  | Site location from map in Simanjuntak [50] | red-slip, cordmarked | [31] |
| Greater Sunda Islands | Borneo/Kalimantan | Liang Abu, Layer 2 | UBA20840 | UC | 1524 | 22 | -41.2 |  | Site location from map in Simanjuntak [50] | red-slip, cordmarked | [31] |
| Greater Sunda Islands | Borneo/Sabah | Bukit Tengkorak, Shelter 1, Square 032, layer 3, 0-5 cm from top of layer | ANU-5769 | UC | 2700 | 110 | - |  |  | red slip, incised, circle-stamped | [29] |
| Greater Sunda Islands | Borneo/Sabah | Bukit Tengkorak, Shelter 1, Square 032, layer 3, 30-35 cm from top of layer | ANU-5770 | UC | 2330 | 170 | - |  |  | red slip, incised, circle-stamped | [29] |
| Greater Sunda Islands | Borneo/Sabah | Bukit Tengkorak, Trench G17, layer 8, 125-130 cm | Beta-83785 | UC | 5330 | 80 | - | C | Spriggs [51]states association with pottery contested, but cannot find reasoning for this. | red slip, incised, impressed | [52] |
| Greater Sunda Islands | Borneo/Sabah | Bukit Tengkorak, Trench G17, layer 5, 95-100 cm |  | UC | 2650 | 90 | - | C |  | red slip, incised, impressed | [52] |
| Greater Sunda Islands | Borneo/Sabah | Bukit Tengkorak, Trench G17, layer 4, 70-75 cm |  | UC | 2940 | 50 | - | A |  | red slip, incised, impressed | [52] |
| Greater Sunda Islands | Borneo/Sabah | Bukit Tengkorak, Trench G17, layer 3, 50-55 cm |  | *Anadara* sp. | 3190 | 60 | - | C |  | red slip, incised, impressed | [52] |
| Greater Sunda Islands | Borneo/Sabah | MAD1, layer 11a | ANU2396 | UC | 2650 | 80 | - | C |  | red-slip, impressed | [28] |
| Greater Sunda Islands | Borneo/Sabah | MAD1, layer 12 top | ANU2398 | UC | **7390** | **270** | - | C |  | preceramic | [28] |
| Greater Sunda Islands | Borneo/Sabah | MAD1, layer 12 top | ANU2397 | UC | **7920** | **370** | - | C |  | preceramic | [28] |
| Greater Sunda Islands | Borneo/Sabah | MAD1, layer 10 | ANU2943 | Fresh-water shell | 2700 | 70 | - | C |  | post initial ceramic assemblage | [28] |
| Greater Sunda Islands | Borneo/Sabah | MAD1, layer 10 base | ANU2945 | UC | 2020 | 90 | - | C |  | post initial ceramic assemblage | [28] |
| Greater Sunda Islands | Borneo/Sabah | MAD1, layer 10 | ANU2395 | UC | 1590 | 150 | - | C |  | post initial ceramic assemblage | [28] |
| Greater Sunda Islands | Borneo/Sarawak | Gua Sireh, Trench F8S, 20-25 cmbs | CA725 | rice husk in sherd | 3850 | 260 | - |  | Fresh water shell dates from site seem to be effected by limestone carbonate | impressed, incised, red-slip | [30] |
| Greater Sunda Islands | Borneo/Sarawak | Gua Sireh, Trench G8N, Layer 4/6 (approx. 23 cmbs) | ANU7049 | UC | 3990 | 230 | - |  | Fresh water shell dates from site seem to be effected by limestone carbonate | impressed, incised, red-slip | [30] |
| Greater Sunda Islands | Borneo/Sarawak | Niah cave, Burial 110 | Rikagaku Kenkyusho laboratory | human bone | 4990 | 90 | - |  | Sample considered "collagen rich" |  | [40] |
| Greater Sunda Islands | Borneo/Kalimantan | Jambu Hilir, test pit w16, 59 cmbs, charcoal concentration beneath sherds | Wk22009 | UC | 2922 | 45 | - |  | sherd descriptions not linked to the dated layer; sherds continue to 90 cmbs and not clear why excavation stopped, so deposit might continue deeper |  | [41] |
| Greater Sunda Islands | Sulawesi | Minanga Sipakko, Unit M1, 160-170 |  | UC | 3500 |  | - | C |  | red slip, incised, impressed | [37:60-67] |
| Greater Sunda Islands | Sulawesi | Minanga Sipakko, Unit M1, 170-180 | Wk-17981 | UC | 3343 | 46 | - | A |  | red slip, incised, impressed | [37:60-67] |
| Greater Sunda Islands | Sulawesi | Minanga Sipakko, Unit M3, 155-170 | Wk-14651 | UC | 3446 | 54 | - | A |  | red slip, incised, impressed | [37:60-67] |
| Greater Sunda Islands | Sulawesi | Minanga Sipakko, Unit M3, 220-240 | Wk-14652 | UC | 3082 | 50 | - | A |  | red slip, incised, impressed | [37:60-67] |
| Greater Sunda Islands | Sulawesi | Minanga Sipakko, Unit M4, 200-210 | Wk-14653 | UC | 2881 | 46 | - | C |  | red slip, incised, impressed | [37:60-67] |
| Greater Sunda Islands | Sulawesi | Minanga Sipakko, Unit M4, 250-260 | Wk-14654 | UC | 2996 | 41 | - | C |  | red slip, incised, impressed | [37:60-67] |
| Greater Sunda Islands | Sulawesi | Minanga Sipakko, Unit M5, 170-180 | P3G-05 | UC | 3690 | 160 | - | C |  | red slip, incised, impressed | [37:60-67] |
| Greater Sunda Islands | Sulawesi | Minanga Sipakko, Unit M5, 260-270 | P3G-05 | UC | 4950 | 180 | - | C |  | red slip, incised, impressed | [37:60-67] |
| Greater Sunda Islands | Sulawesi | Minanga Sipakko, Test Pit 2, 155-160 | P3G-97 | UC | 2570 | 110 | - | C | reported to date "approximate age of uppermost pottery", but do not consider TAQ, exclude from early pottery models | red slip, incised, impressed | [37:60-67] |
| Greater Sunda Islands | Sulawesi | Malawa, Spit 6-7, layer 2 | P3G-06 | UC | 3580 | 130 | - |  | Same lab ref of P3G-06 given for two samples; depth provenience is based on reading of Mahmud 2008, although no specific information is given | red slip, incised, impressed | [35, 36] |
| Greater Sunda Islands | Sulawesi | Malawa, Spit 6-7, layer 2 | P3G-06 | UC | 2710 | 170 | - |  | Same lab ref of P3G-06 given for two samples; depth provenience is based on reading of Mahmud 2008, although no specific information is given | red slip, incised, impressed | [35, 36] |
| Greater Sunda Islands | Sulawesi | Mansiri, Trench D, Layer 4, 143 cmbd | Wk44610 | UC | 2469 | 20 | - |  | date is from charcoal spatially associated with ceramic. Two other 14C dates from site are from upper pottery layer. | red-slip, incised, circle-stamp, dentate | [38] |
| Greater Sunda Islands | Sulawesi | Kamassi, K1-K3 trench, layer 3, 230-240 cm | ANU-35126 | Fresh-water gastro-pod, *Melanoides* sp. | 1620 | 30 | - |  | date inverted for depth; unclear if other decorative techniques, e.g., incising, are associated with these lower spits and dates | red-slip, circle-stamped | [33, 34] |
| Greater Sunda Islands | Sulawesi | Kamassi, K1-K3 trench, layer 3, 240-250 cm | ANU-35127 | Fresh-water gastro-pod, *Melanoides* sp. | 3225 | 30 | - |  | see ANU-35126 | red-slip, circle-stamped | [33, 34] |
| Greater Sunda Islands | Sulawesi | Kamassi, K1-K3 trench, layer 3, 210-220 cm | ANU-35128 | Fresh-water gastro-pod, *Melanoides* sp. | 3140 | 30 | - |  | see ANU-35126 | red-slip, circle-stamped | [33, 34] |
| Greater Sunda Islands | Sulawesi | Kamassi, K1-K3 trench, layer 3, 230-240 cm | ANU-36406 | estruine shell *Geloina* sp. | 3345 | 40 | - |  | see ANU-35126 | red-slip, circle-stamped | [33, 34] |
| Maluku | Morotai | Tanjung Pinang, Layer 1, 5-10 cm | ANU7778 | marine shell | 3390 | 70 | - |  | authors label deposit "preceramic?" and note disturbance from burials, but burials date 1000 years more recent than shell and shell dates by depth in perfect order down layer. Bellwood et al. [46:237]: "The pottery belongs to an incised style widespread during the Indonesian Metal Age, dated to as recently as 700 BP (ANU 7784) at the nearby open site of Sambiki Tua (see Fig. 3). Its overall date range at Tanjung Pinang could thus be between c. 2000 and 500 BP." Claim of 2000 BP for pottery on Morotai is repeated throughout the text, but there is no 14C date associated with this period. | incised, impressed | [46, 53] |
| Maluku | Morotai | Tanjung Pinang, Layer 1, 25-30cm | ANU7779 | marine shell | 4090 | 70 | - |  | authors label deposit "preceramic", sherds at this depth, but none below 30 cm. | incised, impressed | [46, 53] |
| Maluku | Morotai | Tanjung Pinang, Layer 1,30-35cm | ANU7780 | marine shell | 4720 | 70 | - |  |  | no pottery | [46, 53] |
| Maluku | Morotai | Tanjung Pinang, Layer 1, 50-55cm | ANU7781 | marine shell | 5390 | 70 | - |  |  | no pottery | [46, 53] |
| Maluku | Gebe | Um Kapat Papo (UKP) layer 2, 15-20 cm (depth measured from zero at start of layer 2) | ANU9316 | marine shell | 2030 | 60 | - |  |  | incised | [46] |
| Maluku | Gebe | Um Kapat Papo (UKP) layer 3, 5-15 cm (depth measured from zero at start of layer 3) | ANU9317 | marine shell | 4830 | 70 | - |  | authors label this preceramic, but there are sherds in layer 3 (67, 0-10 cm; 7, 10-20 cm) [46:Table 4] | incised | [46] |
| Maluku | Gebe | Golo, | ANU9448 | UC | 3230 | 180 | - |  |  | no pottery | [46] |
| Maluku | Halmahera | Siti Nafisah, Layer A, spit 1, 0-4 cmbs | ANU7785 | estruine shell | 2540 | 70 | - |  | 134 sherds in top 7 cm. Authors state: "Layer A at the surface of the site produced incised and red-slipped pottery dated to c. 2000-1500 BP" but no dates return this range. Date at very top is 2540 +/- 70 , while a date at c. 10 cm is 3410 +/- 70. Excluded from analysis as unclear which determinations relate to pottery-bearing deposits | incised, red-slip | [46] |
| Maluku | Halmahera | Siti Nafisah, Layer B, spit 1, 7-13 cmbs | ANU7786 | estruine shell | 3410 | 70 | - |  | 9 sherds at this depth; excluded from analysis as unclear which determinations relate to pottery-bearing deposits | incised, red-slip | [46] |
| Maluku | Halmahera | Siti Nafisah, Layer C, spit 3, 30-35 cmbs | ANU7787 | estruine shell | 4690 | 120 | - |  | 1 sherd at this depth; excluded from analysis as unclear which determinations relate to pottery-bearing deposits | incised, red-slip | [46] |
| Maluku | Kayoa | Uattamdi, Layer D, 0-15 cm from top of layer | ANU9323 | marine shell | 3260 | 70 | - |  |  | incised, red-slip | [46] |
| Maluku | Kayoa | Uattamdi, Layer D, 15-20 cm from top of layer | ANU7776 | marine shell | 3440 | 110 | - |  |  | incised, red-slip | [46] |
| Maluku | Kayoa | Uattamdi, Layer E | ANU3530 | marine shell | 3530 | 70 | - |  |  | incised, red-slip | [46] |
| Maluku | Pulau Ay | PA1 Unit 3, Layer 6B, 236 cm | Beta-235453 | UC | 3010 | 40 | -24.8 | A |  | incised, red-slip. | [45] |
| Maluku | Pulau Ay | PA1 Unit 3, Layer 6A, 250 cm | Beta-240739 | UC | 3190 | 40 | -28.5 | A |  | incised, red-slip. | [45] |
| Maluku | Pulau Ay | PA1 Unit 3, Layer 4B, 195 cm | Beta-240738 | UC | 3010 | 40 | -24.5 | A |  | red slip | [45] |
| Maluku | Pulau Ay | PA1 Unit 3, Layer 3I 175-185 cm | Beta-304478 | UC | 2940 | 40 | -26.4 | A |  | red slip |  |
| Maluku | Pulau Ay | PA1 Unit 3, Layer 3I, 164 cm | Beta-302405 | UC | 2880 | 30 | -24.1 | A |  | red slip |  |
| Maluku | Pulau Ay | PA1 Unit 3, Layer 3E, 124 cm | Beta-302404 | UC | 2780 | 30 | -23.7 | A |  | red slip |  |
| Maluku | Pulau Ay | PA1 Unit 3, Layer 3C, 105 cm | Beta-235454 | UC | 2460 | 40 | -23.5 | A |  | red slip, circle-stamp |  |
| Maluku | Talaud Islands | LTM site, PQ sector, base layer 4 | ANU-1515 | UC | 4030 | 80 | - |  |  | red-slip, incised, impressed | [47] |
| Maluku | Talaud Islands | LTM site, trench H9, top layer 3 | ANU-1717 | *Turbo* sp. | 4860 | 130 | - |  |  | no pottery | [47] |
| Lesser Sundas | East Timor | Lena Hara Cave, square F, spit 16, 61-68 cm | ANU12029 | charcoal on sherd | 3200 | 240 | -24 |  | sample is described as "charred convex surface of one sherd" | red-slip, plain | [43] |
| Lesser Sundas | East Timor | Lena Hara Cave, square F, spit 16, 61-68 cm | ANU12030 | *T. nilocitus* | 3850 | 70 | - |  |  | red-slip, plain | [43] |
| Greater Sunda Islands | Java | Keplek Cave, B5, 18-30 cm | P3G1998 | UC | 3260 | 110 | - |  |  | earthenware | [32] |
| Greater Sunda Islands | Java | Braholo Cave, L8, 64-80 cm | P3G1999 | UC | 3050 | 100 | - |  |  | earthenware | [32] |
| Greater Sunda Islands | Sulawesi | Topogaro 2, TA-1, spit 11 | TKA-17404 | human tooth | 1900 | 20 | 91.9 |  |  | red-slip, incised, dentate | [39] |
| Greater Sunda Islands | Sulawesi | Topogaro 2, TB-1, spit 6 | TKA-17035 | UC | 2274 | 19 | - |  |  | red-slip, incised, dentate | [39] |
| Maluku | Talaud Islands | Leang Tahuna, Square I5, spit 8 | OZD-771 | *Tridacna* adze | 4310 | 50 | - |  | Author states citing personal communication from Bellwood that this may be "old shell" | red-slip | [54] |
| Maluku | Talaud Islands | Leang Tuwo Mane'e, Square K17, spit14 | ANU-10209 | *Turbo* sp. | 3690 | 70 | - |  | Sherds continue for five spits below this date, although author suggests date is still related to start of ceramic deposition | red-slip | [54] |
| Mussau | Eloaua | ECA | GX-5498 | UC | 3030 | 180 | - |  | From Egloff's original excavations at the site | red-slip, incised, circle-stamp, and dentate | [55] |
| Mussau | Eloaua | ECA, Area A; 1985 Unit W200N149/7, Zone C | ANU-5085 | *Hyotissa* sp. | 3130 | 80 | 0.0 (estimated) |  | Not included in Denham et al.[55] | red-slip, incised, circle-stamp, and dentate | [56] |
| Mussau | Eloaua | ECA, Area A; 1985 Unit W228N102, Level 3 | ANU-5084 | *Tridacna* sp. | 3190 | 80 | 0.0 (estimated) |  | Not included in Denham et al. [55] | red-slip, incised, circle-stamp, and dentate | [56] |
| Mussau | Eloaua | ECA, Area A; TP-9 (Unit W400N72), Level 6, 40-70 cm bs | ANU-5080 | UC | 3260 | 90 | -24.0 (estimated) |  |  | red-slip, incised, circle-stamp, and dentate | [56] |
| Mussau | Eloaua | ECA, Area A; Unit W250N90, Level 2 | Beta-30676 | *Turbo marmoratus* | 3590 | 110 | 1.9 |  | Not included in Denham et al. [55]; Kirch argues that *Turbo*'s habitat preference along the outer reef warrants an "open ocean" delta R of 0; cf. Petchy & Ulm's [57] delta R value | red-slip, incised, circle-stamp, and dentate | [56] |
| Mussau | Eloaua | ECA, Area B; 1985 Unit W199N151, Post B2 | ANU-5791 | *Intsia bijuga* (outer 2 cm, including bark) | 2930 | 80 | -24.0 (estimated) |  | Same stilt house as ANU-5790, different post | red-slip, incised, circle-stamp, and dentate | [56] |
| Mussau | Eloaua | ECA, Area B; combined sample from Unit W200N150, Levels 12 & 13, Zone C1 | ANU-5079 | UC | 2840 | 115 | -24.0 (estimated) |  | Post-dates the stilt house | red-slip, incised, circle-stamp, and dentate | [56] |
| Mussau | Eloaua | ECA, Area B; Unit W198N145, Post B30, Level 7, Zone C3 | Beta-20452 | Unid. wood (outer 2 cm, including bark) | 3050 | 70 | -24.0 (estimated) |  |  | red-slip, incised, circle-stamp, and dentate | [56] |
| Mussau | Eloaua | ECA, Area B; Unit W200N120, ~130 cm bs | Beta-20451 | *Cocos nucifera* (endocarp) | 2950 | 70 | -24.0 (estimated) |  |  | red-slip, incised, circle-stamp, and dentate | [56] |
| Mussau | Eloaua | ECA, Area B; Unit W200N150, Post B1 | ANU-5790 | *Intsia bijuga* (outer 2 cm, including bark) | 2950 | 80 | 24.0 (estimated) |  | Same stilt house as ANU-5791, different post | red-slip, incised, circle-stamp, and dentate | [56] |
| Mussau | Eloaua | ECA, Area B; Unit W200N151, Level 11, Zone C3 | ANU-5081 | *Tridacna gigas* | 3010 | 80 | 0.0 (estimated) |  | Representing deposition around stilt house; Not included in Denham et al. [55] | red-slip, incised, circle-stamp, and dentate | [56] |
| Mussau | Eloaua | ECA, Area B; Unit W201N149, Level 12, Zone C3 | ANU-5082 | *Hyotissa hyotis* | 2950 | 80 | 0.0 (estimated) |  | Representing deposition around stilt house; Not included in Denham et al. [55] | red-slip, incised, circle-stamp, and dentate | [56] |
| Mussau | Eloaua | ECA, Area C; Unit W249N188, Level 2 | Beta-30674 | *Hippopus hippopus* | 3110 | 70 | 1.0 |  | Kirch notes that this date and Beta-30675 are anomalously early for Area C and may represent midden discarded in the area previously; Not included in Denham et al. [55] | red-slip, incised, circle-stamp, and dentate | [56] |
| Mussau | Eloaua | ECA, Area C; Unit W249N188, Level 4 | Beta-30675 | *Tridacna derasa* | 3110 | 80 | 1.9 |  | Kirch notes that this date and Beta-30674 are anomalously early for Area C and may represent midden discarded in the area previously; Not included in Denham et al. [55] | red-slip, incised, circle-stamp, and dentate | [56] |
| Mussau | Eloaua | ECA, Area C; Unit W250N188, Post C3 | Beta-30686 | *Diospyros* sp. | 2850 | 70 | -24.5 |  | Based on ceramics and geomorphology Kirch argues occupation of this area post-dates the earliest occupation of Areas A & B | red-slip, incised, circle-stamp, and dentate | [56] |
| Mussau | Eloaua | ECA, W250 transect; Unit W250N100, Level 2 | Beta-30677 | *Spondylus* sp. | 3170 | 70 | 1.2 |  | Not included in Denham et al. [55] | red-slip, incised, circle-stamp, and dentate | [56] |
| Mussau | Eloaua | ECA, W250 transect; Unit W250N110, Level 15 | Beta-30679 | *Tridacna gigas* | 3080 | 70 | 2.3 |  | Not included in Denham et al. [55] | red-slip, incised, circle-stamp, and dentate | [56] |
| Mussau | Eloaua | ECA, W250 transect; Unit W250N110, Level 4 | Beta-30678 | *Chama* sp. shell | 3190 | 80 | 2.1 |  | Not included in Denham et al. [55] | red-slip, incised, circle-stamp, and dentate | [56] |
| Mussau | Eloaua | ECA, W250 transect; Unit W250N120, Level 6 | Beta-30680 | *Chama* sp. shell | 3320 | 80 | 2.8 |  | Kirch flags as a "rogue" date, but cf. Petchey & Ulm's [57]delta R; Not included in Denham et al. [55] | red-slip, incised, circle-stamp, and dentate | [56] |
| Mussau | Eloaua | ECA, W250 transect; Unit W250N120, Level 9, wooden post | Beta-30681 | Unid. wood | 2860 | 60 | -30.5 |  |  | red-slip, incised, circle-stamp, and dentate | [56] |
| Mussau | Eloaua | ECA, W250 transect; Unit W250N140, Level 6, wooden post | Beta-30682 | Unid. wood | 2970 | 50 | -28.1 |  |  | red-slip, incised, circle-stamp, and dentate | [56] |
| Mussau | Eloaua | ECA, W250 transect; Unit W250N150, Level 7 | Beta-30683 | *Hippopus hippopus* | 3140 | 80 | 2.7 |  | Not included in Denham et al. [55] | red-slip, incised, circle-stamp, and dentate | [56] |
| Mussau | Eloaua | ECA, W250 transect; Unit W250N170, Level 3, wooden stake | Beta-30684 | Unid. wood | 3100 | 110 | -28.6 |  |  | red-slip, incised, circle-stamp, and dentate | [56] |
| Mussau | Eloaua | ECB, Etakosarai; 1985 transect Unit 1, Level 1 | ANU-5086 | *Hyotissa hyotis* | 3120 | 80 | 0.0 (estimated) |  | Three determinations from ECB believed to relate to a single, short-duration Lapita occupation; not included in Denham et al. [55] | red-slip, incised, circle-stamp, and dentate | [56] |
| Mussau | Eloaua | ECB, Etakosarai; 1985 transect Unit 1, Level 2 | ANU-5087 | *Hyotissa hyotis* | 3150 | 80 | 0.0 (estimated) |  | Three determinations from ECB believed to relate to a single, short-duration Lapita occupation; not included in Denham et al. [55] | red-slip, incised, circle-stamp, and dentate | [56] |
| Mussau | Eloaua | ECB, Etakosarai; 1986 transect Unit 9, Level 5, 71 cm bs | Beta-20453 | UC | 3200 | 70 | -24.0 (estimated |  | Three determinations from ECB believed to relate to a single, short-duration Lapita occupation | red-slip, incised, circle-stamp, and dentate | [56] |
| Mussau | Emirau | EQS, Tamuarawai; Test Pit 1, Layer 4 | Wk-21349 | UC | 3044 | 31 | -23.4±0.2 |  | Depositional context interpreted as having formed in a low energy lagoonal environment | red-slip, incised, circle-stamp, and dentate | [48] |
| Mussau | Emirau | EQS, Tamuarawai; Test Pit 2, Layer 4 | Wk-21345 | UC | 2917 | 31 | -27.4±0.2 |  | Depositional context interpreted as initial build-up of calcareous beach, post-dating lagoonal occupation | red-slip, incised, circle-stamp, and dentate | [48] |

*: UC = unidentified charcoal

†: A = accelerator mass spectrometry, C = conventional, blank cell = unstated
