# Supplementary material for: The first quantitative assessment of radiocarbon chronologies for initial pottery in Island Southeast Asia supports multi-directional Neolithic dispersal
Source: PLoS One. 2021 Jun 2;16(6):e0251407. doi: 10.1371/journal.pone.0251407 (PMC8171956; doi:10.1371/journal.pone.0251407)
Supplement: Attachment — Submitted filename: Cochrane_et_al_Response_to_reviewers.docx [file pone.0251407.s003.docx]

**Response to Reviewers**

Dear Editor,

Here we outline the changes made in our revised submission that address editor and reviewer comments. These changes are also visible in the track-changes version of our manuscript and Supporting Information. The changes are extensive and specific, so please pardon the lengthy list that follows.

Regarding the general editor comments about subjective treatment of the models, we have now provided consistent justification for the inclusion of particular dates and models, and accepted the advice of Reviewer 3 concerning models with fewer than four dates. Moreover, unused dates and models are now included in the SI for readers to evaluate.

The Funding Statement and Competing Interest Statement have been amended as requested and are included in our cover letter.

Figure 1 copyright issu*e*: Figure 1 uses an Esri basemap and was created with a licensed version of ESRI ArcGIS software. According to the Esri Master Agreement (a section of which is pasted below with relevant phrases highlighted) we can use this image, provided we include a statement in the caption stating Esri is the source of the basemap.

The plain language summary of the Esri Master Agreement also confirms this, a section of which is copied below:

Therefore we propose, to amend the figure 1 caption as required to the Esri agreement and have done so in our revision. If this is not acceptable, please let us know.

Reviewer 1 comments:

*Abstract*: we have addressed most of the reviewers noted typos and points of style.

*P. 4 discussion on dates*: We are referencing the 3450 date used by the primary proponent, Bellwood, of the OoT scenario. We have now added a citation that questions the 3450 dates. Regarding Palau this group is typically considered a few hundred years more recent than the Marianas and is not typically referenced by the Out of Taiwan model we are summarising here and thus have not included it in the discussion.

*Including or not including potentially problematic dates and how this effects results*: we have addressed this issue (similarly brought up by Reviewer 3) in the following ways:

- What we have done is not simply chronometric hygiene and we now describe more clearly our approach (paragraph beginning line 98) and distinguish it from chronometric hygiene approaches like Spriggs (1989) [now cited in paper].
- Our results include only models with five or more dates as Reviewer 3 makes the valid point that Bayesian models with four or fewer dates do not improve accuracy or precision. Models with fewer dates, although not used in our discussion or conclusions, are included in the SI in a separate section.
- We have now made our reasons for accepting or rejecting particular radiocarbon determinations more clear, although we have done this in the SI, not the main text. To do this in the main text in a systematic fashion across all models would be, we think, too much detail (see, e.g., our SI discussion of Nagsabaran). If the editor thinks otherwise, we can put the detail in the main text. Where relevant in the SI we note if samples have, or do not have, isotopic values, if there are adequate calibrations (e.g., for shell), and if the sample type, such as resin, may be introducing unknown error. If multiple models can be constructed for a deposit, we include all the models in the SI and state why we use our preferred model with the particular dates it includes.
- We chose not to alter figure 1 with population movement arrows as we believe these are too simplistic or, if done well (i.e., showing multiple various movement combinations), would clutter the image. If the editor would like us to make this change, however, we will do so.
- We think our revisions address the reviewer comment: “Overall, I would like to see some more effort on showing what would happen if certain problematic dates were excluded, and perhaps variations of these (e.g., what would the model look like if certain (or all) dates did not fit the criteria imposed…”

Reviewer 2 comments:

Reviewer 2’s comments are difficult to productively address as they are mostly innuendo, unsupported by data. However, we note the following:

- The reviewer suggests our argument is based on a straw man hypothesis. We dispute this as the OoT hypothesis is routinely used by name and numerous studies interpret their results as supporting it. A few such studies (some in very high ranked outlets) include:
  - 1.Bellwood P, Dizon E. The Batanes Archaeological Project and the "Out of Taiwan" Hypothesis for Austronesian Dispersal. *Journal of Austronesian Studies*. 2005;1(1):1-33.
  - 2.Ricaut F-X, Thomas T, Mormina M, Cox MP, Bellatti M, Foley RA, et al. Ancient Solomon Islands mtDNA: assessing Holocene settlement and the impact of European contact. *Journal of Archaeological Science*. 2010;37(6):1161-70. doi: DOI: 10.1016/j.jas.2009.12.014.
  - 3.Chang C-S, Liu H-L, Moncada X, Seelenfreund A, Seelenfreund D, Chung K-F. A holistic picture of Austronesian migrations revealed by phylogeography of Pacific paper mulberry. *Proceedings of the National Academy of Sciences.* 2015;112(44):13537-42. doi: 10.1073/pnas.1503205112.
  - 4.Buckley CD, Boudot E. The evolution of an ancient technology. *Royal Society Open Science*. 2017;4(5):1-21. doi: 10.1098/rsos.170208.
- The reviewer states that we are both ignorant of East Asian archaeology and do not evaluate assemblage integrity. It’s hard to address the first charge as the reviewer provides no evidence of the detrimental effects of our ‘ignorance’. Regarding the second charge, our paper is almost wholly focused on assessing radiocarbon date integrity in the context of assemblage depositional integrity. We can only assume that the reviewer uses a different, unstated, meaning than we do for “assessing the integrity of the assemblages”. If the reviewer means we should remove dates that appear anomalous by using ad hoc criteria or assumptions about dispersal hypotheses that is unfortunate as this is exactly the kind of reasoning we are trying to avoid in this paper for reasons we state in the introduction.
- We now cite examples of research that use ad hoc dating analyses to bolster their favoured hypotheses. The reference to Dye is not an example of this, but Dye makes the same argument for the Pacific Islands.
- The reviewer states that ours is only a summary presentation of the record. There is no other way to describe a record as extensive as this without a book-length treatment, so we disagree that this is a flaw.
- The reviewer has mistaken our estimates for onset of ceramic deposition at Emirau for calibrated age ranges of the radiocarbon determinations.
- We now use the 2020 calibration models and most recent delta R values.

Reviewer 3 comments:

This reviewer has provide many helpful comments that we address below. The reviewer’s comment about our title seems a bit strange. We use ‘Bayesian’ to describe the method, which on its own does not equal robusticity. Also we think our results support multi-directional movement more than either the WRM or OoT hypotheses, so we see no reason to change out title.

*Reviewer states we have not justified our methods:* The two paragraphs at line 98 and 113 address this and make a better case for our use of Bayesian methods.

*Reviewer states that we reject freshwater shells dates, but do not provide evidence of their problematical nature:* For the sites used in our analysis, Nagsabaran and MAD1, have freshwater shell-derived determinations and one site that we do not use has only freshwater or estuarine shell determinations (Kamassi). As described in the SI, we modelled Nagsabaran and MAD1 with and without the shell determinations and note that there are differences in the results. The fact that the five oldest determinations from the shell midden layer at Nagsabaran are on shell causes us to question the Hung et al evaluation of correction factors using modern shell that is millennia more recent as the carbon reservoir could have change over time (as pointed out by Petchey et al. 2018 *Geochronology*). For MAD1 the exclusion of shell dates has negligible effects on the results (10 years). Our conservative approach is to exclude the shell determinations. This does not obviate the possibility for a freshwater reservoir effect, as the weight of each individual freshwater shell-derived determination on the model results—particularly with the use of the outlier commands—is weak in relation to the other determinations. We agree with the reviewer that the potential for a freshwater reservoir effect should be evaluated for specific archaeological locales, but this is beyond the scope of our paper.

*Reviewer notes problems with unidentified wood charcoal (dates that are too old) and charcoal outlier command in Oxcal:* We agree with the reviewer. The alternative—creating a single-phase model without the Charcoal Outlier command—may result in the converse: an estimate that is too young. The root issue is a poor corpus of dates. However, as explained in our manuscript, we are demonstrating that a quantitative modelling approach does produce results different from visual inspection. Either approach requires better data.

*Reviewer notes issues with bone dates and reporting isotopic values:* Our text has been revised, clarifying that the human bone dates from Pain Haka were included and calibrated because isotopic values are reported. Some bone isotope data was obtained from the Waikato lab, allowing us to use additional determinations. Radiocarbon data in S3 Table has been corrected.

*Reviewer states we are inconsistent in our treatment of residues:* This is incorrect. We note that the inclusion of residue-derived determinations for Batan Island significantly increases the start date, and select the model that excludes these determinations. For Anaro Hilltop, all determinations are derived from pottery residue and we appropriately qualify the results. It appears the reviewer has misread our text when stating that we exclude older residue dates and accept younger reside dates--here we are describing what Bellwood and Dizon have done (lines 256-259).

*The reviewer argues that models with fewer than five determinations are no better than eyeballing results:* we have taken the reviewer’s advice and removed models with fewer than five determinations.

*The reviewer note additional model issues including convergence values, charcoal outlier u values, stratigraphy:* we have now calculated convergence values and these exceed 95% for all models, unless otherwise stated; regarding u values it is not clear what models in particular the reviewer is referencing, but we have run some models with different Charcoal Outlier u values (0,1 to 0,6) and find that this parameter has no or only a negligible effect on the start estimate results; we use stratigraphy in our models where possible and note this; we have rounded the dates in Table 1 to 10 years.

*The reviewer would like our discussion of Nagsabaran to be more detailed, summarizing the problems and potentials of dating the site*: we have now done this in the section beginning line 382 in the SI.

*The reviewer would like to see more discussion about the non-dating evidence that supports multi-directional movement*: we now provide this on lines 377-401; and while more could be written we feel that might be over-interpreting our results at this stage.

Reviewer 4 comments:

*The reviewer notes that our preference for the WRM hypothesis over OoT is not supported and that neither hypothesis is correct:* with our reanalysis using some methodolgocial advice from reviewer 3, we agree and have revised our text to reflect the ambiguity and propose that multi-directional movement is currently most supported (as also proposed by Reivewer 4).

*The reviewer notes issues with MAD1*: in part the reviewer is mistaken about our use of pre-ceramic dates; these are used to constrain date estimates in a Bayesian model and the procedures are detailed in the SI and main text at paragraph beginning line 459. Also for MAD-1, we agree with the reviewer that the imprecise estimate for the appearance of pottery (boundary between Layers 12 and 11a) is not terribly analytically meaningful; however, methodologically, it highlights the generally poor data quality for statistically robust chronology-building in the region.

*The reviewer notes issues with Gua Sireh:* Gua Sireh has been removed from our analysis because it does not meet our newly established sample size cut-off (>4 radiocarbon determinations). Again, we agree with the reviewer that the small number of determinations and the large error ranges for two of the determinations result in an imprecise estimate. Use of the boundary estimate, however, is preferable to selecting the HPD estimates for individual modelled dates: the former estimate considers the total corpus of modelled data to provide an estimate for the appearance of pottery as an event that is not directly dated. Relying on individual modelled dates assumes that those dated events coincide with the exact timing for the initial appearance of pottery at the deposit, which may or may not be correct. Text in the Materials and Methods section explains this, paragraph beginning line 480.

*The reviewer proposed that the arrival of ceramics in Luzon and Timor is contemporaneous:* this proposal seems to be based on individual or select dates. As our analysis reveals, larger, more robust data sets are required for Timor, Luzon, and other islands in the region to better differentiate the appearance of pottery at each location.

*The reviewer notes that use of the charcoal outlier with prior probability of 1 dominates all models and creates greater uncertainty (i.e., larger ranges):* The reviewer is correct, but this uncertainty is simply inherent in the dating material. With a large (~40+) data set that includes a majority, or at least equal number, of determinations from charcoal of identified short-lived taxa or plant parts, the effect of the charcoal outlier command on the unidentified charcoal determinations is better down-weighted. Until such data sets exist, however, we prefer a conservative approach that applies the current outlier probability. We have revised the outlier parameters, though, to limit the outlier distributions between 0-200 years (see code for specific models in the SI), which in general is probably a better estimation of potential inbuilt age. We will add that most other dating materials—charred residue on sherds, marine shell, freshwater shell, and animal or human bone—bring their own challenges as we discuss in the manuscript and SI.

*The reviewer agrees with the multi-directional interpretation and would like more discussion on the linguistic, biological and archaeological ramifications and literature*: this comment is echoed by Reviewer 3 and we have addressed it on lines 377-401.

Minor errors noted by Reviewer 4 have also been corrected.
