# Supplementary material for: The first quantitative assessment of radiocarbon chronologies for initial pottery in Island Southeast Asia supports multi-directional Neolithic dispersal
Source: PLoS One. 2021 Jun 2;16(6):e0251407. doi: 10.1371/journal.pone.0251407 (PMC8171956; doi:10.1371/journal.pone.0251407)
Supplement: Attachment — Submitted filename: Response_to_reviewer_final.docx [file pone.0251407.s004.docx]

Dear Dr Pinhasi

Thank you for the opportunity to present a revised version of our manuscript. This letter details the changes we have made in response to reviewer comments.

**Reviewer #1:**

We have changed the keywords in response to Reviewer 1’s comments.

Esri’s documentation does not capitalise the name, so we have not done this.

We have modified figure 1 to include the directional arrows requested. We did not put date ranges on the figure as we think this will be overly complicated and to be clear we would have to include departure time-frames and arrival time-frames. We think this description is best left in the text.

**Reviewer #3:**

We have changed our title to Reviewer 3’s suggestion.

We have replaced the term estimate throughout the document.

Reviewer comment: “…a certain level of negativity about the dates beyond fact creeps into the text. For example, the use of “sometimes” in Line 237. Suggest instead “dates to between 5440-4270”. Suggest careful evaluation of comments about age ranges.

- We use sometimes (with the ‘s’) once in the manuscript to note what researchers sometimes do. When we discuss date ranges the word is always ‘sometime’ (without the ‘s’), so we think this is not negative, just indicating that the date could be any date within the range.

We did not “highlight the regional/Island results” in table 1 as we think it makes the table messy, i.e., some contiguous rows would be highlighted and others not. If the editor would like however, we can make this change.

Reviewer comment: Note: in table the number of samples from Luzon is 24, but Nagsabaran is 27. The reason for this is discussed in the text, but a footnote to the table would clarify this to the reader.”

- We have done this.

Reviewer comment: Line 175: The residue-derived determinations do not add 250 years to the dates. The range shifts by ~250years but the new range overlaps include part of the old range.

- We have added a parenthetical statement clarifying this older shift in the date range.

Reviewer comment: Line 175: delta13C should be superscript (throughout text and tables).

- Done.

Reviewer comment: Throughout the paper: The use of a deltaR of 0 with Marine20 is problematic. Marine20 is offset relative to Marine13 (Reimer et al. 2013) by at least 100 years (Heaton et al. 2020), so a correction of at least this is required to keep the calibrations compatible with evaluations that have previously used Marine13….

- We have added an endnote, which states the offset.

Reviewer comment: Lines 3463 onwards: The model for Lena Hara uses Marine13 and a deltaR of 0. This is not compatible with the use of Marine20 elsewhere (see comment above). Also, Um Kaput Papo, Uatlandi and ECB.

- We have corrected this to Marine 20.

Reviewer comment: SI figures: Colour the regional plots a different shade or colour from the site distributions. S2 figure: Specify as footnote which model result for Nagsabaran has been used.

- We have done this.

Reviewer comments: S3 Table:delta13C should be superscript. Lab codes need to be standardized, e.g., ANU-001 or ANU001, not both. Bone material should be specified, i.e., dentine vs enamel, gelatin vs ultrafiltered. Stable isotope data for the bone dates should be included (they are discussed, but I cannot find the specific data anywhere in the table). These have an impact on the reliability of the result. References to bone interpretation should be given. Specify in Table S3 which deltaR values have been used. The references given in the table for the deltaR values used were all calculated pre-Marine20, therefore an updated value using the base data from those references should be given to ensure clarity.

- We have made all these changes and included the requested information when available (e.g., when bone material is specified). Regarding delta R values, this only applies to Pain Haka and ECA; the other line items relate to sites that we do not included in our final analysis.

Reviewer comment: Line 2153: Please define what poor convergence means. You cannot assume that the reader is familiar with these definitions.

- Explanation added after the first reference to convergence values (in the discussion of the Nagsabaran site)

With best wishes,

Ethan Cochrane
